# Capturing the Value of Vaccination within Health Technology Assessment and Health Economics—Practical Considerations for Expanding Valuation by Including Key Concepts

**DOI:** 10.3390/vaccines12070773

**Published:** 2024-07-15

**Authors:** Eliana Biundo, Mariia Dronova, Annie Chicoye, Richard Cookson, Nancy Devlin, T. Mark Doherty, Stephanie Garcia, Antonio J. Garcia-Ruiz, Louis P. Garrison, Terry Nolan, Maarten Postma, David Salisbury, Hiral Shah, Shazia Sheikh, Richard Smith, Mondher Toumi, Jurgen Wasem, Ekkehard Beck

**Affiliations:** 1 GSK, Building W23, 20 Avenue Fleming, 1300 Wavre, Belgiumstephanie.x.garcia@gsk.com (S.G.); hiral.a.shah@gsk.com (H.S.); sheikh.shaz1a@gmail.com (S.S.);; 2 Putnam PHMR, 30-701 Krakow, Poland; mariia.dronova@putassoc.com; 3 AC Health Consulting, Sciences Po, 75007 Paris, France; annie.chicoye@gmail.com; 4 Centre for Health Economics, University of York, York YO10 5DD, UK; richard.cookson@york.ac.uk; 5 Health Economics Unit, Centre for Health Policy, University of Melbourne, Melbourne 3010, Australia; nancy.devlin@unimelb.edu.au (N.D.); t.nolan@unimelb.edu.au (T.N.); 6 Department of Pharmacology and Clinical Therapeutics, Faculty of Medicine, University of Malaga, 29071 Malaga, Spain; ajgr@uma.es; 7 School of Pharmacy, University of Washington, Seattle, WA 98195, USA; lgarrisn@uw.edu; 8 Department of Health Sciences, University Medical Center Groningen, University of Groningen, 9700 AB Groningen, The Netherlands; m.j.postma@rug.nl; 9 Department of Economics, Econometrics & Finance, Faculty of Economics & Business, University of Groningen, 9713 AB Groningen, The Netherlands; 10 Center of Excellence in Higher Education for Pharmaceutical Care Innovation, Universitas Padjadjaran, Bandung 40132, Indonesia; 11 Programme for Global Health, Royal Institute of International Affairs, Chatham House, London SW1Y 4LE, UK; david.salisbury@btinternet.com; 12 College of Medicine and Health, University of Exeter, Exeter EX1 2HZ, UK; rich.smith@exeter.ac.uk; 13 Creativ-Ceutical, 1724 Luxembourg, Luxembourg; mondher.toumi@creativ-ceutical.com; 14 Institute for Health Care Management and Research, University of Duisburg-Essen, 45127 Essen, Germany; juergen.wasem@medman.uni-due.de

**Keywords:** economic evaluation, efficiency, health equity, macroeconomic, health systems strengthening, vaccination

## Abstract

Following the development of a value of vaccination (VoV) framework for health technology assessment/cost-effectiveness analysis (HTA/CEA), and identification of three vaccination benefits for near-term inclusion in HTA/CEA, this final paper provides decision makers with methods and examples to consider benefits of health systems strengthening (HSS), equity, and macroeconomic gains. Expert working groups, targeted literature reviews, and case studies were used. Opportunity cost methods were applied for HSS benefits of rotavirus vaccination. Vaccination, with HSS benefits included, reduced the incremental cost-effectiveness ratio (ICER) by 1.4–50.5% (to GBP 11,552–GBP 23,016) depending on alternative conditions considered. Distributional CEA was applied for health equity benefits of meningococcal vaccination. Nearly 80% of prevented cases were among the three most deprived groups. Vaccination, with equity benefits included, reduced the ICER by 22–56% (to GBP 7014–GBP 12,460), depending on equity parameters. Macroeconomic models may inform HTA deliberative processes (e.g., disease impact on the labour force and the wider economy), or macroeconomic outcomes may be assessed for individuals in CEAs (e.g., impact on non-health consumption, leisure time, and income). These case studies show how to assess broader vaccination benefits in current HTA/CEA, providing decision makers with more accurate and complete VoV assessments. More work is needed to refine inputs and methods, especially for macroeconomic gains.

## 1. Introduction

Vaccination programmes have proven highly effective at protecting against morbidity and mortality from infectious diseases, thereby reducing the burden on populations and health systems. The assessment of vaccines considers these benefits in the health technology assessment and cost-effectiveness analysis (HTA/CEA), which informs recommendations for inclusion in the National Immunization Programmes (NIPs). Developed countries using HTA that focusses on CEA, however, consider various concepts for the value of vaccination (VoV), which can result in differences in vaccination policies across, and even within, countries. In addition, assessments currently take a narrow value perspective [1,2], not considering many of the broad benefits that vaccination brings, for example, to society and the national economy. The COVID-19 pandemic highlighted many value concepts of vaccination, e.g., reducing fear and anxiety, preventing long-term disability, reducing health inequalities, relieving the burden on health systems, scientific spillovers, and economic productivity and activity [3,4,5,6,7].

In the first part of this research (Beck et al., 2022 [8]), a review of published VoV frameworks and value concepts identified 18 unique vaccination benefits. A novel VoV framework was tailored specifically to the HTA/CEA setting in developed countries to enable future extensions of VoV considerations captured. The framework categorises value concepts into three dimensions: (I) those conventionally included in HTA/CEA from a payer perspective (i.e., health gains to vaccinated individuals and direct medical cost savings); (II) those conventionally included from a societal perspective (i.e., indirect health and economic gains for caregivers and health-related productivity gains for vaccinated individuals); and (III) broader novel societal perspective concepts (e.g., decreased antimicrobial resistance, financial risk protection, and macroeconomic gains).

In the second part of this research (Postma et al., 2022 [9]), country HTA/CEA guidelines (from Australia, France, Germany, the Netherlands, Spain, the United Kingdom [UK], and the United States) were reviewed to assess which of the identified value concepts are currently included in vaccine assessments. Of the 18 VoV concepts defined in part one [8], four are routinely included in current HTA/CEA, while some others may be assessed in ad hoc cases. Experts in vaccine HTA/CEA provided a system for ranking the importance of each concept based on their potential impact on decision-making and how feasible it is to quantify them within HTA/CEA. This analysis was able to prioritise three additional benefits of vaccination for inclusion in HTA/CEA that should be the focus of attention in the near to mid-term future, i.e., (1) health systems strengthening, resilience and security; (2) social equity and ethics; and (3) macroeconomic gains.

The objective of this final part of the research is to provide decision makers with an overview of methods for capturing vaccination value more broadly, with practical steps for including the three novel value concepts in HTA/CEA in the near term. The overview provides potential methods to be used in HTA/CEA with case studies where feasible, highlights relevant decision-making considerations, and some suggestions for evidence generation for each of the three novel concepts with reflection on the methodological limitations. Supplementary Materials S1 provides a summary slide deck of this work.

## 2. Methodology

First, for each of the three key concepts (i.e., health systems strengthening, resilience, and security; social equity and ethics; and macroeconomic gains), a targeted literature review was conducted to refine the concept definitions and identify potential methodologies for concept assessment as a part of HTA/CEA (Supplementary Materials S2).

Then, the literature findings were then discussed in expert working groups. Experts were allocated to one of three concept working groups based on their expertise and interest. The HTA/CEA experts from high-income countries with well-established HTA and health systems (involved throughout the entire study in the framework development and concept prioritisation), together with concept-specific experts (macroeconomics and equity), worked to build consensus on the methods for including each concept in vaccine HTA/CEA. Finally, a plenary advisory board brought together the entire expert panel to consolidate the overview of future methodological applications for the three concepts in these countries.

Finally, a practical application of the methods was illustrated through case studies. An existing economic model, where available, was adapted to include an assessment of the new concept. The potential impact on the cost-effectiveness of including vaccine benefits of ‘Health systems strengthening, resilience and security’ and ‘Social equity and ethics’ was thus assessed retrospectively, for paediatric rotavirus [10] and meningococcal B disease [11] vaccination programmes, respectively. For the potential impact of including vaccine “Macroeconomic gains’, suggestions were made for future model adaptations, based on current non-HTA/CEA methods that are used in macroeconomic impact assessment.

## 3. Health Systems Strengthening (HSS), Resilience, and Security Using Opportunity Cost Methods

Kutzin and Sparkes (2016) [12] define HSS as comprising the means needed (i.e., through policies) to improve universal health coverage, health systems resilience and security. Universal health coverage refers to the provision of effective health services to all people, without causing financial hardship [12] and is in place in most developed countries. Health systems resilience is the ability to adapt and continue to provide quality services when faced with disturbances such as pandemics, natural disasters or changes in disease burden [12]. Health systems security refers to the ability to protect society from health threats originating from other countries [12].

Following the discussion with experts, vaccination could be assumed to primarily help strengthen health systems and therefore improve their resilience and security by impacting two drivers; health systems efficiency (HSE), e.g., quality of care, costs, effectiveness of interventions [13,14]; and health systems capacity (HSC), e.g., capacity of resources such as hospital/intensive care unit beds and the medical workforce (e.g., nurses, physicians, general practitioners) [13,15], with these two drivers interacting and impacting each other. Based on findings of the targeted literature review, Table 1 shows some existing health economics methods potentially applicable for capturing benefits of vaccination for HSE and HSC.

An expert discussion led to a selection of the opportunity cost approach, as this method is well developed with illustrative case studies available [13,16], and only required adaptation for inclusion into CEA. In addition, decision makers are aware of issues relating to opportunity costs due to the consequences of COVID-19 e.g., burden on health systems leading to overcrowding and delayed care.

The ‘opportunity cost’ is described as the benefit foregone for the next best alternative use of resources, expressed in monetary terms [16], e.g., in health economics, it is the cost of the health foregone by not treating the next best patient due to allocation of scarce resources (e.g., beds, medical staff, and equipment) to other patients. Vaccination reduces the incidence of vaccine-preventable diseases (VPDs), thereby reducing the burden on health systems, and freeing up resources to treat other diseases. Opportunity cost approaches consider both economic (i.e., costs) and health (e.g., quality-adjusted life-years [QALY]) outcomes, which is important for compatibility with CEA. The hospital burden and corresponding opportunity costs were previously investigated in England, for norovirus infections leading to gastroenteritis hospitalisations [13]. Due to the infectious nature of the disease, the opportunity cost calculations considered not only the burden from infected cases, but also the wider health and economic impact due to outbreaks within the hospital, staff absences from infection, and beds becoming unavailable for patients with other diseases due to infection control measures (e.g., decontamination). The foregone costs and benefits (from not treating non-norovirus patients) outweighed those from treated norovirus gastroenteritis patients, resulting in an overall loss of health and costs [13].

When assessing vaccine impact on drivers of HSS, and choosing the appropriate methodological approach, a distinction should be made between non-epidemic, epidemic and pandemic effects on health systems. The vaccination impact and its magnitude will vary greatly in each case. The existing capacity and average utilisation levels need to be considered, e.g., in countries, such as the UK, that operate at or above target utilisation levels, where increased respiratory infections during winter (‘winter stress’) put pressure on demand for hospital beds [15]. Studies have shown that constrained capacity in the intensive care unit can increase the risk of mortality, as well as healthcare costs [20,21]. Vaccines that prevent respiratory VPDs, such as respiratory syncytial virus (RSV), pneumococcal, or influenza vaccines, could free up healthcare resources and have an impact on HSS during winter stress [15], which can be assessed using opportunity cost approaches.

**Figure 1:**
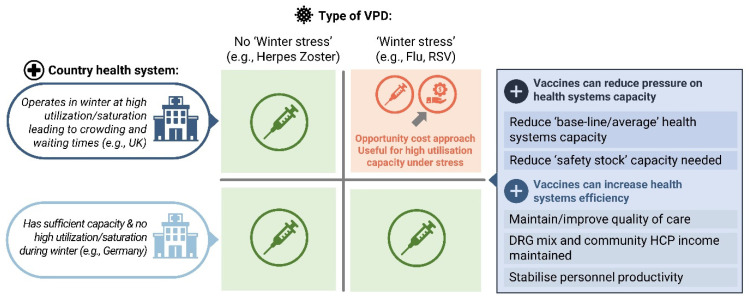
Assessing vaccination impact in health systems with constrained versus normal utilisation. DRG: diagnosis related group; Flu: influenza; HCP: healthcare professional; RSV: respiratory syncytial virus; VPD: vaccine-preventable disease.

For systems with constrained capacity in high utilisation times (e.g., ‘winter stress’ in the UK), peak load opportunity cost can be considered a large non-marginal change in the system, which is novel to CEA compared with the sum of small marginal changes/cost savings in the system already captured by CEA. Thus, the proposed opportunity cost method can be applied to capture non-marginal differences associated with peak incidence during winter stress, when hospitals are exceeding maximum capacity. For systems with no constrained capacity (i.e., sufficient hospital bed capacity even during winter stress times) vaccination impact can be quantified via reduction in hospital beds needed, from a queuing theory, safety stock perspective, and/or improvement in efficiency/quality of care within existing bed capacity (Figure 1, Table 1). No method, however, could be identified to readily incorporate this impact into CEA.

### 3.1. Case Study for HSS Benefits Using Opportunity Costs: Rotavirus Vaccination

A case study using infant rotavirus vaccination provided a clear example of (a) peak incidence of a VPD (i.e., rotavirus gastroenteritis, RVGE) occurring during winter, (b) significant reduction in RVGE hospitalisations following vaccination introduction [22], and (c) evidence that utilisation of paediatric wards did not decrease following reduction in RVGE-related paediatric hospitalisations, suggesting beds became available for non-RVGE cases [23]. For this illustrative modelling exercise, a previously published CEA of infant rotavirus vaccination in the UK [10] was re-analysed, using a simple calculator to capture the impact of vaccination on HSS and how this affects cost-effectiveness, using opportunity cost methodology. The methodological approach proposed by Sandmann et al. [13] for estimating the opportunity cost of bed-days was extended, to allow for integration into the CEA and estimation of the incremental cost-effectiveness ratio (ICER) (Supplementary Materials S1). In the paediatric setting, every child with acute and urgent need for care is assumed to be treated without significant delay, therefore, the rationale for considering opportunity costs and next-best alternatives was based on children waiting for non-urgent/acute and planned treatments, such as planned elective surgeries. Due to limited evidence on corresponding outcomes, multiple exploratory scenarios were considered (Supplementary Materials S1). Besides planned surgeries (referral to a treatment scenario), upper respiratory tract infections (URTI) were used as a proxy for alternative conditions, since the peak incidence seasons for rotavirus and URTI largely overlap, and URTI is reported as one of the most common causes for hospitalisation [24]. Alternative scenarios also considered non-gastroenteritis patients with chronic conditions and without chronic or life-threatening conditions. First, the public health impact with and without vaccination was assessed, estimating foregone bed-days and patient-equivalents (i.e., how many other patients could have been treated instead) due to RVGE. Second, the impact on cost-effectiveness was estimated. This analysis considered additional health services that could be provided to children with the resources available in the absence of RVGE hospitalisations, and their health benefits and costs. The results obtained, therefore, reflect the broad benefits of vaccination, as well as the efficiency of alternative uses of resources. Considering the public health impact (i.e., considering forgone bed-days, or forgone patient equivalents [PE]), rotavirus vaccination made available up to 33,006 additional beds over the first five years in the vaccinated cohort, to be used for the treatment of 5501 to 57,083 patients, depending on the type of alternative hospitalisation. Considering the approaches for estimating opportunity cost, with foregone health benefit for the next-best PE or foregone health benefit for the best alternative use (i.e., treatment-equivalents [TE]), rotavirus vaccination could provide 20–1461 additional QALYs for other patients benefitting from available resources, depending on the alternative hospitalisation, reducing the ICER to GBP 11,552–GBP 23,016/QALY compared with the baseline ICER (with no inclusion of opportunity costs) of GBP 23,337/QALY gained, based on cost and QALY estimates reported by Martin et al. [10] (Table 2, see Supplementary Materials S3 for details and scenario analyses).

Several approaches including health benefits with associated costs (e.g., using gross expenditure saved, or foregone net/gross monetary benefit for the next-best PE) found that opportunity cost estimates varied widely according to parameters of the alternative hospitalisation (cost, length of stay, QALY gain, and the respective resource use efficiency) and how they compared to parameters of RVGE hospitalisation (Supplementary Materials S3).

### 3.2. Policy Considerations

Building upon the methodological approach to estimate opportunity costs by Sandmann et al. [16], this case study demonstrated that the inclusion of opportunity cost considerations in CEA is possible, and illustrated the additional benefits that vaccination brings to health systems by freeing up resources faster to treat patients with non-VPD conditions. A key advantage of the approach is that it could be captured by the health systems or payer perspective typically used in HTA/CEA and thus does not require applying a societal perspective. In addition, the opportunity cost approach gives decision makers a broader perspective when evaluating the benefits of a particular program by demonstrating the benefits that can be achieved across other programs as well (e.g., surgeries). In particular, the public health impact estimation may also allow the capture of the impact of vaccination in resource and capacity planning for non-pandemic, pandemic, or disaster scenarios. Rotavirus primarily affects infant hospitalisation during a defined period (winter months), therefore, the impact of freeing up hospital beds and other resources would likely result in reduced waiting times for other planned treatments. In the case of VPDs and the vaccination of older adults (e.g., against influenza, pneumococcal, RSV, and herpes zoster), however, much larger opportunity cost and impact of freeing up healthcare resources may be expected. It may be even more important to capture this, because older adults may experience worse and non-reversible health outcomes from delays in planned treatment, e.g., preventable deaths or long-term complications due to temporary worsening of their health state while waiting. Thus, capturing the opportunity costs relating to vaccination in this age group is extremely important [13].

### 3.3. Methods and Evidence Considerations

There is considerable uncertainty regarding the nature of other diseases for which a bed becoming available would be used, and it could be reasonably assumed that those could be occupied by patients with different conditions (e.g., different types of surgeries). To address this uncertainty, the analysis included various alternative hospitalisation scenarios. Dedicated evidence generation studies would be needed to inform such analyses and provide the relevant data for the target population and intervention of interest. These might include studies aiming to better quantify health state utilities pre- and post-surgery, as well as comprehensively assessing adverse outcomes, including related costs and quality of life, in adults waiting for treatment. Further studies estimating opportunity costs should also capture the impact on (a) a broader range of resources, (b) settings beyond the hospital (e.g., primary care visits, nurse time, emergency visits, etc.), (c) target populations beyond children, and (d) additional benefits from the decreased pressure on healthcare facilities. For example, there are usually many more cases requiring GP/outpatient care (although with lower severity) than there are hospitalised cases. Vaccination could help to reduce the healthcare burden for general practitioners, especially during winter stress months, and these benefits can also be demonstrated with an opportunity cost approach. In addition to the health benefits for the vaccinated individual, the same types of broader vaccination benefits could be considered with such an approach, e.g., freeing up capacity to treat other diseases (with additional capacity for GPs if nurses/other healthcare professionals administer vaccines) and impact on quality of care and efficiency in the GP practice. Further method considerations include using a different opportunity cost according to the time horizon used, e.g., likely to be highest in the first year of infant vaccination, and lower once transmission is reduced (e.g., through herd immunity, which is captured in a limited way in static models).

## 4. Social Equity and Ethics Using Distributional Cost-Effectiveness Analysis

Health inequalities, disparities, or inequities are differences in health between more and less socially disadvantaged groups that are systematic, socially produced—for example, by poverty, discrimination and unequal access to services including health services, and unfair [25].

**Figure 2:**
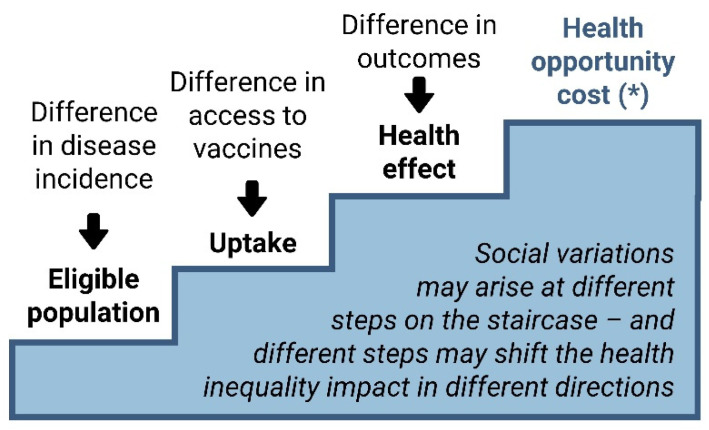
Staircase of health inequality impact [Based on a figure developed by Cookson and Love-Koh, Centre for Health Economics, University of York]. * Health loss due to intervention costs: scarce resources would otherwise be used to improve health in other ways.

In Figure 2, the staircase of health inequality impact provides a stepwise conceptual approach to capture the impact of interventions on health inequalities in general, as well as considering its application in HTA/CEA through distributional cost-effectiveness analysis (DCEA). It illustrates how social variations may arise at each stair. Notably, different stairs may shift the health inequality impact in different directions, either improving or worsening health inequalities.

In the context of VPDs and vaccination, health equity stratifiers allow the identification of health inequalities in the population, by disaggregating relevant health indicators, such as VPD incidence or prevalence. Equity strata can be defined by socioeconomic status, race/ethnicity, geographic location, disability, sex, gender, sexual orientation, or other factors. Socioeconomic status, race/ethnicity, and geographic location are well-known risk factors for the transmission and acquisition of several infectious diseases [26,27], and are the most commonly considered equity stratifiers in DCEA [28]. As many of these equity strata contribute to social deprivation, and thus health inequalities overall, indices of deprivation have been developed capturing multiple equity strata, e.g., the index of multiple deprivation (IMD) in England [29], or the social vulnerability index in the US [30]. However, as only few countries have developed such indices, high-quality data are mostly available for the socioeconomic stratifiers, which reflects decision makers’ increased awareness of this social determinant of health. Clearly, the final selection of stratifiers used in each country can differ and must reflect country-specific priorities.

Regarding uptake of vaccination, NIPs, as a part of universal healthcare coverage, provide an opportunity for fair, often free, access for the whole population; however, uptake rates can vary across health equity strata. Lower vaccination uptake has often been correlated with socioeconomic background (e.g., lower education level) and ethnicity [31,32].

The health effects can also be influenced by health equity strata, such as differences in access to healthcare. Treatment and recovery from acute infectious diseases and long-term treatment of sequelae can be affected, resulting in worse health outcomes in populations with poor access to healthcare [33]. Further, socioeconomically disadvantaged groups tend to have a lower baseline health, i.e., lower overall quality of life/health state utility and lower life-expectancy. The impact of an infectious disease in these groups may, thus, be larger than in non-disadvantaged groups, even considering all groups face the same QALY losses from a disease [34].

Whereas the first three stairs of the staircase (Figure 2) can be applied to any type of analysis of the impact of health technologies (e.g., public health impact analysis), the health opportunity cost stair for vaccination (within a fixed budget scenario) is specific to CEA/DCEA, as it relates to the question of efficient allocation of more healthcare resources for vaccination to alleviate health inequalities arising within the first three stairs, at the cost of limited funding of other health services. Evidence on the distribution of opportunity costs is limited, and on current evidence, it is not unreasonable to assume that the health opportunity costs of healthcare expenditure are equally distributed across different socioeconomic groups [35].

### 4.1. Approach to Considering Health Equity in Vaccination HTA/CEA

The impact of vaccination on health inequality can be studied with a stepwise approach, which could also be applied to other health technologies, as outlined below.

In step 1, the impact of vaccination on health equity can be assessed by comparing total health outcomes, such as total infections, deaths, or QALYs per health equity strata quintile in scenarios with and without vaccination, as net health opportunity costs. The advantage of this step is that it can provide straightforward insights into the direction and magnitude of the impact of vaccination on health inequalities, considering health outcomes only. This step is similar to the traditional ‘public health impact analysis’ in HTA/CEA assessments of vaccines.

**Figure 3:**
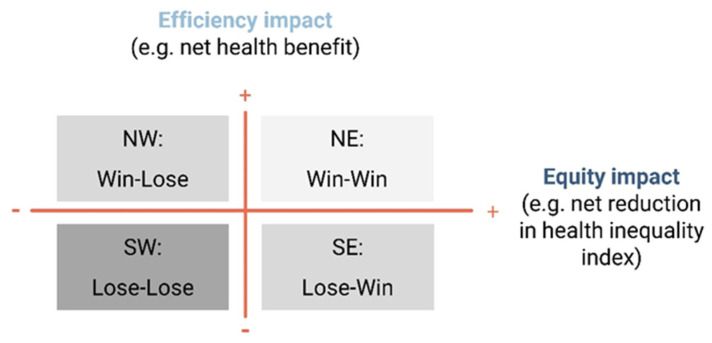
Equity-efficiency impact plane. NE: north-east; NW: north-west; SE: south-east; SW: south-west (based on [37]).

In step 2, the standard CEA efficiency impact measures, such as the net health benefit, can be visually compared with the impact on equity using health inequality indices in a two-by-two plane (Figure 3), with four possible combinations of the CEA conclusions and potential equity impact [36].

In step 3, DCEA can be applied to study both efficiency and health inequality impacts considering social preferences to reduce health inequality, using inequity aversion parameter or ‘health inequality aversion’, via Kolm–Pollak and Atkinson social welfare functions (SWF) [38]. This allows systematic exploration of the implications of equity weights applied to health gains and losses for people in multiple social groups. An alternative approach, known as ‘threshold weighting’, is to apply a single equity weight to the recipient population of the intervention—though this is harder to achieve in a systematic and comparable way since the appropriate weight will depend on the social composition of the recipient population and the wider general population. With only limited evidence available on the magnitude of the inequity aversion parameter, sensitivity analysis may be used to determine the impact of changes in this parameter.

### 4.2. Case Study for Equity Benefits Using DCEA: Meningococcal B (MenB) Infant Vaccination

To illustrate the methodologic options for the equity case study and assess feasibility, the example of MenB vaccination against invasive meningococcal disease in England was used. Socioeconomic status is a known risk factor for MenB invasive meningococcal disease [39]. The case study was conducted in a retrospective fashion to obtain insights into how consideration of equity may have impacted cost-effectiveness and thus decision making. An existing CEA for meningococcal B vaccination (4CMenB, GSK) in England [11] was adapted to consider the additional benefits of vaccination on health equity using DCEA. The three-step approach described above was used to account for equity in the public health impact and CEA for MenB vaccination in England (see Supplementary Materials S4 for details).

The model was adapted to allow for consideration of total QALYs as the main outcome per treatment arm to inform health equity analysis, in line with the published literature on DCEA and expert opinion. The model was populated with data for the English setting: inequality aversion parameters reflecting population preferences (i.e., 10.95 [Atkinson SWF, reflecting relative inequality in health benefit] and 0.15 [Kolm–Pollak SWF, reflecting absolute inequality in health benefit] [38]), and Index of Multiple Deprivation Quintiles (IMDQ)-stratified inputs for population distribution, carriage prevalence, vaccination coverage, life expectancy, utility, and annual income.

The original model included a quality-of-life adjustment factor (QAF) based on a QALY weighting of three to account for society’s preferences to prevent very severe diseases such as MenB-related cases of invasive meningococcal disease. As these preferences for prioritising severe illness may potentially intersect with preferences for reducing health inequality, two reference cases were explored; with a QAF equal to three and QAF equal to one (i.e., equivalent to no QALY weighting for severity).

**Figure 4:**
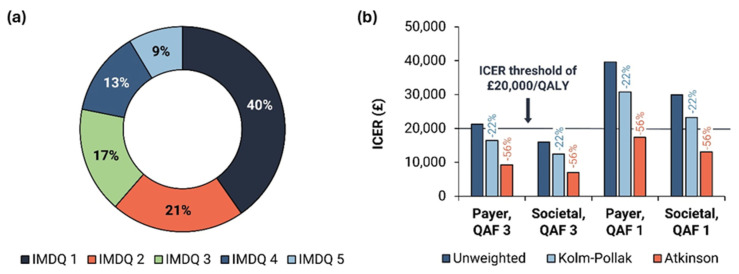
(**a**) MenB cases prevented across equity strata and (**b**) the impact of including equity benefits on cost-effectiveness. ICER: incremental cost-effectiveness ratio; IMDQ: index of multiple deprivation quintile; MenB: MenB-related cases of invasive meningococcal disease; QAF: quality-of-life adjustment factor; QALY: quality-adjusted life-year.

The results obtained suggest that 4CMenB infant vaccination disproportionately prevented MenB cases among more deprived groups; of all prevented cases, 40% were among the most deprived IMDQ 1 (accounting for 26% of the target population under five years of age) and 78% among the three most deprived IMDQs (Figure 4a).

The analysis showed that 4CMenB vaccination improved equity (i.e., it has positive net equity impact) in the population studied, with the net equity benefit being robust to changes in distribution of uptake, MenB carriage prevalence, life expectancy and utility stratified by IMDQ. For the analysis considering a QAF of three and a societal perspective, 4CMenB vaccination could be in the ‘win-win’ quadrant of the equity-efficiency impact plane, being both cost-effective and improving equity (see Supplementary Materials S4 for impact plane and more results). Considering Kolm–Pollak’s absolute and Atkinson’s relative QALY weighting in the step 3, the unweighted (baseline) ICER (i.e., GBP 16,035 and GBP 29,919, with QAF of three and one, respectively) was reduced by 22% with Kolm–Pollak (to GBP 12,460 and GBP 23,261, respectively) and by 56% with Atkinson (to GBP 7014 and GBP 13,114, respectively) (Figure 4b). Almost all presented options in Figure 4b have an ICER below the GBP 20,000/QALY threshold, regardless of QAF. Deterministic (DSA) and probabilistic sensitivity analysis (PSA) showed a positive net equity benefit across the majority of simulations, with weighted ICERs not exceeding the threshold (Supplementary Materials S4).

### 4.3. Policy Considerations

This case study demonstrated that implementing DCEA into formal vaccine HTA/CEA is possible and can provide important information for decision makers interested in improving health inequality. This clear quantitative approach can, thus, be used to answer questions previously informed by qualitative reflection on observed evidence (e.g., for the extension of human papillomavirus vaccination to boys in the UK [40]). Considering the magnitude of effect, e.g., reducing the ICER by up to 56%, inclusion of equity considerations could have aided decision making. The reduction of health inequalities is an inherent value proposition of an NIP, as an important building block of universal healthcare coverage, and should, therefore, be explicitly considered in future HTA/CEA.

Future work, including the update and development of HTA/CEA guidelines by National Immunisation Technical Advisory Groups (NITAGs) and HTA bodies (e.g., update of Canadian guidelines with recommendations on including equity considerations in vaccine CEA [41]), should focus on providing guidance on expectations regarding which equity stratifiers to consider and prioritise, to allow for the proper evidence generation and planning of analysis. In the absence of fully stratified data on various outcomes needed for DCEA (e.g., ranging from incidence to direct medical cost and productivity losses), the minimum data stratification needed to estimate the health equity impact are incidence and coverage stratified by the selected equity parameter (e.g., income, race, etc.), which would allow exploration of the distribution of outcomes (step 1 above).

In countries where a cost-effectiveness threshold is used, a reflection should be made on how the inclusion of equity in CEA modifies value judgements. Considering both threshold weighting and QALY weighting approaches (see Supplementary Materials S2 for details), the DCEA case study illustrated that the QALY weighting approach is more relevant for vaccines, as it includes a parameter for society’s aversion to inequality, via the SWF. Further, the SWF approach is also applicable in settings without a formal cost-effectiveness threshold.

Lastly, the relationship between societal preferences regarding the prevention of disease severity and preferences for health equity should be further explored; criteria for interpreting equity measures further refined; and dedicated evidence generation studies conducted, especially in countries where data stratified by socioeconomic status are not routinely reported.

### 4.4. Methods Considerations

The adaptations required to account for equity stratifiers, such as socioeconomic status, are relatively straightforward to implement in static cost-effectiveness models, however, it is challenging to stratify a dynamic transmission model (DTM) retrospectively [42], as this would require stratifying mixing patterns and risk exposure. If equity needs to be considered, it will be important to ensure at the design stage, especially for a dynamic model, that the data processing and results generated by the DTM are compatible with the DCEA.

Traditionally, vaccine CEA focuses on prevented cases and associated QALY gains and losses. However, DCEA is conventionally based on total QALY levels over a lifetime—quality-adjusted life expectancy—which requires QALY gains and losses to be added onto baseline estimates of lifetime health without vaccination. This allows any measure of health inequality to be used, including relative health inequality measures, such as the Atkinson SWF, based on proportional changes relative to baseline health. In principle, however, DCEA could be conducted based on health gains and losses only, using a Kolm–Pollak SWF or other measures of absolute health inequality that do not require estimating proportional changes relative to baseline health. This should be considered at the model design stage.

While CEA considers average health benefits for the total population, DCEA uses the equally-distributed equivalent level of health (EDEH) taking into account SWFs and inequality aversion parameters (e.g., 10.95 [Atkinson] and 0.15 [Kolm–Pollak]). As different SWFs reflect different types of inequality in health benefit (Kolm–Pollak ‘absolute’; Atkinson ‘relative’), further development of criteria allowing to better interpret equity measures in specific context settings (e.g., prevention versus treatment; common versus rare disease) could also facilitate implementation of the DCEA framework in the formal decision-making process. As inequality aversion parameters are likely to be country-specific, estimating these may require dedicated evidence generation studies.

## 5. Macroeconomics Gains Using Computable General Equilibrium Models

Traditional CEA analysis helps NITAGs and HTA bodies to evaluate the costs and benefits of a new vaccine. Assessing the efficiency, i.e., the value for money, is important because the allocation of resources to a vaccination programme presents an opportunity cost in terms of the other benefits that could have been achieved with these funds. At present, HTA/CEA uses microeconomic models to inform healthcare policy decisions, with a focus on the impact of disease and interventions on the patient and healthcare sector. Healthcare decision makers are familiar with these methods but not accustomed to considering broader macroeconomic impacts of diseases and interventions. By contrast, other decision makers, such as finance ministers, typically use macroeconomic models to inform their policies, and might be much more willing to invest in an intervention with a positive impact on the national economy (or subsectors).

A macroeconomic approach to assessing disease impact and prevention through vaccination on economic welfare should, therefore, consider the combined impact of disease on economic components relating to non-health consumption, leisure time, and health status [43,44]. Disease may, for instance, increase healthcare costs, reduce the labour force and productivity and reduce investment in human and physical capital, which can all have an important impact on macroeconomic outcomes [45]. Of note, HTA/CEA microeconomic models also consider the impact of disease on healthcare costs, labour, and productivity, although from a much narrower perspective compared with macroeconomic models.

**Figure 5:**
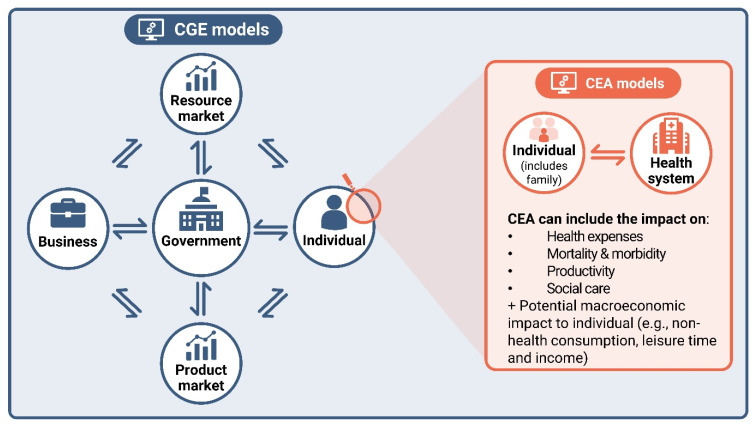
Illustrative diagram of the different elements that could be included in a CGE model and links with microeconomic CEA models. CEA: cost-effectiveness analysis; CGE: computable general equilibrium.

The expert panel agreed that the commonly used macroeconomic computable general equilibrium (CGE) models are suitable to capture the macroeconomics gains concept. CGE models are multi-sectoral models of the whole economy and have been applied to health policy interventions, e.g., COVID-19, influenza, and antimicrobial resistance (AMR) [46,47,48]. CGE models include multiple production sectors and goods markets and a government budget, which allows us to capture publicly funded health costs. The model demonstrates the impact on gross domestic product (GDP) by capturing the behaviour of different economic agents in the economy (Figure 5) and can also be tailored to describe the impact on one or more specific sectors of the economy. The behaviour of different agents is based on economic theory, specified mathematically with a system of equations solved simultaneously.

A CGE model was previously applied to assess the macroeconomic impact of AMR in the UK, i.e., the impact on productivity, labour supply, and healthcare costs [48]. A CEA model considers increases in treatment duration, morbidity, and mortality due to AMR and corresponding increased costs to the healthcare system, patients/households, and productivity losses if the societal perspective is applied. The CGE model, however, allows for consideration of increased morbidity and mortality and the corresponding reduction in the labour supply and productivity, leading to a drop in national output and national income, which in turn reduces national savings and welfare. With fewer national savings, there is reduced investment and a further decline in the economy. The increased healthcare costs required to treat AMR reduces costs and resources for other diseases, ultimately reducing societal welfare [48]. In the case of AMR, the effects on labour and productivity had a larger impact on reducing GDP than the increased healthcare costs.

With our focus on non-pandemic vaccines, it is important to note that the impact on GDP might be relevant in some sub-sectors, while the overall economy impact may be relatively limited.

The first step to including the macroeconomic gains concept in HTA is to assess how CEA and CGE model results can complement each other and which inputs are common to both methods (Figure 5), to illustrate the direction of impact of broader value components. Parameters relevant to both CGE and CEA models, based on current value considerations in HTA/CEA frameworks [9], are mortality, morbidity, medical and healthcare costs, productivity, social care and informal caregivers (e.g., significant others taking care of their partners, adults taking care of their children/parents, and grandparents looking after grandchildren), and individual behaviours beyond health impacts.

Disease and vaccination impacts, in both CEA and CGE models, are quantified via variations in direct health expenditure, social care, labour and productivity loss. In CEA, the impact on productivity is estimated by aggregating individual productivity/labour losses. The opportunity costs of preventing productivity losses by avoiding disease is estimated, in most cases, in terms of lost wages for the individual. In CGE models, the impact on productivity loss in the entire workforce is included, as well as the effects that a change in the labour force has on other parts of the economy. As a result of a reduction in productivity, an individual will see a reduction in income, leading to lower consumption, lower taxation, and potentially a greater need for monetary transfers from the government. A reduction in consumption will reduce demands for goods and services, leading to a reduction in supply, affecting changes in labour force demand and, in turn, taxation. These aggregated effects will have an impact on GDP. CGE models use the same broad approach for estimating the impact of disease and vaccination on direct health expenditure and on social care.

### 5.1. Policy Considerations

Adding this system perspective may help to inform the true value for money in an intervention and help to finance interventions that deliver a better return on investment from a governmental point of view. Additionally, understanding how an intervention affects different economic agents can also inform decision making on where to place emphasis to achieve broad policy objectives. Some studies on possible ways of integrating macroeconomic and microsimulation models (which are not used as standard in HTA/CEA) have shown that this is a complex process in which inconsistencies in each models’ datasets are difficult to overcome, as well as the interaction between the models and how the outcomes of one model affect the other [49]. Another approach, in the field of vaccines, included the impact of taxes lost as a result of lost productivity within a microeconomic model, to provide insights on return on investment from a governmental viewpoint and inform funding decisions [50]. Thus, the proposal to capture macroeconomic impact in assessment and recommendation of vaccines by NITAGs and HTAs is, therefore, to complement CEA with considerations on the macroeconomic impact. This could be achieved either by presenting a complementary CGE model that illustrates how the impact on productivity of a vaccine affects the broad economy, or by estimating the cost to the broad economy and including it as a fixed cost within the CEA. The latter approach also requires a reflection on how to account for a multiplier for productivity.

### 5.2. Methods Considerations

Considerations for a possible macroeconomic case study, to help illustrate the magnitude of impact and to demonstrate the relevance of this VoV concept for NIPs in non-pandemic settings, could be to assess the impact of adult vaccination against respiratory diseases en bloc. This would reflect the magnitude of the unmet need to be addressed by adult immunisation for the population aged 50 years and older. The case study could assess the impact in this age group, including the impact on labour and productivity, as well as in individuals aged 65 years and older, where different sectors of the economy are likely to be affected, e.g., hospitals, social care, and informal care. The impact and value of social care could be estimated by mapping how changes in informal care affect productive agents, e.g., if a grandparent is sick, the parent might have to stay home to take care of the child, hence impacting labour participation in other groups.

## 6. Discussion

The current evaluation of vaccines in HTA/CEA lacks a consistent approach for considering the broader value benefits, with consequences for access to vaccination. In particular, three key VoV concepts that have the potential to be included in assessments in the near future are HSS, health equity, and macroeconomic gains. Discussion of policy and methodological considerations, as well as case studies, showed that the benefits of vaccination to health systems can already be assessed in HTA/CEA using the opportunity cost approach, while the impact on equity can be assessed using DCEA. Additional research on methods for incorporating vaccination impact on health systems capacity/improvement in quality of care and on macroeconomic gains is urgently needed to provide decision makers with more complete and relevant information on the true value of vaccines, which could currently be underestimated in CEA. A review of the HTA criteria used in Western and Asian countries found that countries consistently use standard clinical safety and efficacy/effectiveness and economic (CEA and budget impact) criteria [51], but there was no discussion of additional value criteria [51]. A comparison of European economic evaluation guidelines found that around half of the countries recommended a societal perspective, which allows for some broader value criteria [52]. However, no further details were provided on what should be included or how. The importance of key VoV concepts is high, as reflected by the experts and demonstrated by the COVID-19 pandemic, and these should already be considered as additional analyses and reflected in the deliberative process.

Case studies were used to illustrate the practical application of the proposed approaches for the three concepts. The selection of examples was driven by model availability, relevance of the concept for a given VPD, and expert consultation. A limitation was the lack of a model to illustrate the macroeconomic gains approach. In the case of the equity case study, England was selected as it was the only country with an established index for deprivation (IMD) and the data available for the proper design of the DCEA. Efforts to develop indices and generate equity-stratified evidence on disease burden are also ongoing in other countries, such as Australia [53], Chile [54], and the US (e.g., extension of the social vulnerability index (SVI) [55]. A considerable amount of work still needs to be performed to refine methodological guidance and generate evidence to support the assessment of these new vaccination value concepts. Another hurdle for expanding the use of these methods and value concepts is that they rely on countries applying a broader societal perspective rather than a narrow payer perspective. This is not, however, the case for vaccination impact on HSS, which will need to be applied from a health systems or payer perspective. This approach was limited by focusing on methods, from the literature or expert input, that can be readily applied in vaccines HTA/CEA; however, alternative methods may be available. While the focus was on evaluating vaccination programs, the methods proposed could be applied broadly to other healthcare technologies.

The appeal of current CEA methods is that they are well understood, accepted, and applied, being the ‘workhorse’ of vaccine HTA/CEA assessments [8] by healthcare decision makers, and provide funding-relevant information on efficiency and value for money. The key disadvantage, however, is that traditional CEA does not capture the broader societal perspective well, particularly where vaccination provides benefits to other stakeholders beyond patients and the healthcare sector. For example, estimates from a macroeconomic model on the impact of Ebola on employment, income, and economic growth were previously used to promote rapid prevention and disease management measures in West Africa [56]. Different stakeholders can require different types of evidence on the VoV. Payers and final policy decision makers, such as the Ministry of Health and the Ministry of Finance, may require outcomes such as the return on investment using macroeconomic impact considerations, whereas policy decision makers, such as NITAGs, are more concerned with the impact within the healthcare sector. If applicable, the societal perspective is used, although often limited to productivity loss considerations from an economic perspective [57]. Despite including broader ‘novel societal’ concepts, these CEAs are still likely to be limited in capturing the value of vaccines compared with other methods, such as cost-benefit analysis or multi-criteria decision analysis, though less applied, and for stakeholders beyond NITAGs/HTAs. Expanding value considerations in CEA, however, provides a near-term feasible opportunity to capture broader value concepts transparently and quantitatively. This may also allow traditional stakeholders involved in the assessment and development of recommendations to interact more closely and discuss the value of NIPs with other stakeholders, such as final policy decision makers, more holistically, as more aspects relevant to these other stakeholders are captured in the analysis. Considering a broader VoV can ultimately allow countries to allocate sufficient budgets beyond narrow healthcare considerations, e.g., to reflect benefits for education or social care budgets that are often separate from health budgets and thus enable more timely access to vaccines across the whole life course of the population.

This paper discusses the practical application of the three priority concepts that the expert panel selected. Despite the focus on the near-term feasibility to implement these concepts in vaccines HTA/CEA, the VoV framework developed in this study includes several additional concepts that will need to be further explored to better capture the full value of vaccines.

## 7. Conclusions

The aim of this work has been to broaden the valuation of vaccination programs within the context of HTA/CEA. It started with the development of a VoV framework that includes a comprehensive overview of unique VoV benefits [8]. Subsequently, an assessment of the key gaps in current vaccine guidelines led to the identification of three priority concepts that can be included in economic assessments in the near term [9]. And finally, a proposal has been put forward with methods applicable to consider equity benefits, HSS benefits, and macroeconomic gains associated with vaccination. The case studies presented here demonstrate the feasibility of including these concepts in HTA/CEA of vaccines in the near term and illustrate how, in the past, decision makers may have underestimated the value of vaccination (i.e., vaccination programs on rotavirus and MenB) by not formally including assessment of these aspects of societal benefits. This study paves the way to applying these proven methods in future HTA assessments.

## Figures and Tables

**Table 1:** Economic methods potentially applicable for capturing vaccination impact on health systems strengthening.

Methodologies	HSE Benefit	HSC Benefit
Opportunity cost [16]	Less VPD implies more time, resources, budget available for treatment of patients with other diseases [13]	Less VPD implies more capacity for treatment of patients with other diseases [13]
Optimisation model	Optimal allocation of resources to increase efficiency across different programmes [14]	Optimal allocation of new investments, e.g., vaccination versus beds [15]
Microsimulation model	Fewer VPDs can benefit quality of care metrics, within existing capacity [17,18]	No literature identified
Hospital capacity planning; queuing theory; logistics/supply chain theory; systems-level model	No literature identified	Fewer VPDs can reduce admission and target occupancy rates, and LOS from secondary infections [19]

HSC: health systems capacity; HSE: health systems efficiency; LOS: length of stay; VPD: vaccine-preventable disease.

**Table 2:** Impact of including opportunity cost on rotavirus cost-effectiveness.

Scenarios of Alternative Conditions Treated	RTT Admitted	URTI	URTI Mild	URTI Severe	Chronic Condition (Non-Gastroenteritis)	Non Chronic Condition (Non-Gastroenteritis)
Inputs for scenarios						
Cost of hospitalisation	GBP 1364	GBP 409	GBP 307	GBP 511	GBP 1270	GBP 1270
Length of stay (days)	1.2	1.0	0.5	2.1	5.4	3.3
QALY gain associated with hospitalisation	0.0047	0.0014	0.0010	0.0017	0.239	0.002
Results: Gross health benefit foregone for the next-best patient-equivalents						
Additional health benefit (QALYs gained)	132	45	65	27	1461	20
ICER	GBP 21,374	GBP 22,624	GBP 22,322	GBP 22,907	GBP 11,552	GBP 23,016
Results: Health benefit foregone for the best alternative use (TE) *						
Additional health benefit (QALYs gained)	614					
ICER	GBP 22,336					

Note: the baseline ICER without considering opportunity costs was GBP 23,337/QALY gained (based on cost and QALY estimates from Martin et al. [10], using 2006 costs). * The opportunity cost and ICER estimates are the same in all scenarios, as this approach does not specify how the newly available resources will be used. ICER: incremental cost-effectiveness ratio; QALY: quality-adjusted life-year; RTT: referral to treatment with hospital admission (e.g., referral for general surgery); TE: treatment equivalents; URTI: upper respiratory tract infections.

## Data Availability

The data presented in this study are available in this article and Supplementary Materials.

## Supplementary Materials

The following supporting information can be downloaded at: https://www.mdpi.com/article/10.3390/vaccines12070773/s1, Supplementary Materials S1: Summary slide deck; Supplementary Materials S2: Targeted literature reviews; Supplementary Materials S3: Case study: Rotavirus re-analysis with opportunity cost; Supplementary Materials S4: Case study: MenB vaccination with distributional cost-effectiveness analysis. References [10,11,13,16,22,23,24,28,29,36,38,39,58,59,60,61,62,63,64,65,66,67,68,69,70,71,72,73,74,75,76,77,78,79,80,81,82,83,84,85,86,87,88,89,90,91,92,93,94,95,96,97,98,99,100] are cited in the Supplementary Materials.
