# Supplementary material for: Capturing the Value of Vaccination within Health Technology Assessment and Health Economics—Practical Considerations for Expanding Valuation by Including Key Concepts
Source: Vaccines (Basel). 2024 Jul 15;12(7):773. doi: 10.3390/vaccines12070773 (PMC11281546; doi:10.3390/vaccines12070773)
Supplement: Supplementary file 1 [file vaccines-12-00773-s001.zip › Supplementary Materials S1.pdf]

Capturing the Value of Vaccination within health technology assessment and health economics

Practical considerations for including key concepts

Eliana Biundo, Annie Chicoye, Richard Cookson, Nancy Devlin, Mark Doherty, Mariia Dronova, Stephanie Garcia, Antonio J Garcia-Ruiz, Louis P Garrison, Terry Nolan, Maarten Postma, David Salisbury, Hiral Shah, Shazia Sheikh, Richard Smith, Mondher Toumi, Jurgen Wasem, Ekkehard Beck

The corresponding study and publication was funded by GSK

Overview

1. Draft Value of vaccine framework & concepts
2. Expert consensus on framework
3. Country analysis
4. Expert consensus on top 3 concepts
5. Practical considerations for including key concepts in HTA/CEA

- **Steps 1 & 2:** The development of a **novel Value of Vaccination (VoV) framework** designed to broaden the consideration of vaccination value in Health Technology Assessment (HTA) or Cost-Effeciveness Analysis (CEA) (Beck *et al*.)
- **Steps 3 & 4:** The framework was used to conduct a **country analysis** and to determine the **top 3 value concepts** to prioritise for HTA/CEA inclusion (Postma *et al*.)
- **Step 5:** This presentation of the final step explores **methods and practical considerations** for including the top 3 value concepts in HTA/CEA

CEA: cost-effectiveness analysis; HTA: Health technology assessment; VOV: Value of Vaccination

Background

- In the initial steps, we defined a **VoV framework for HTA/CEA**

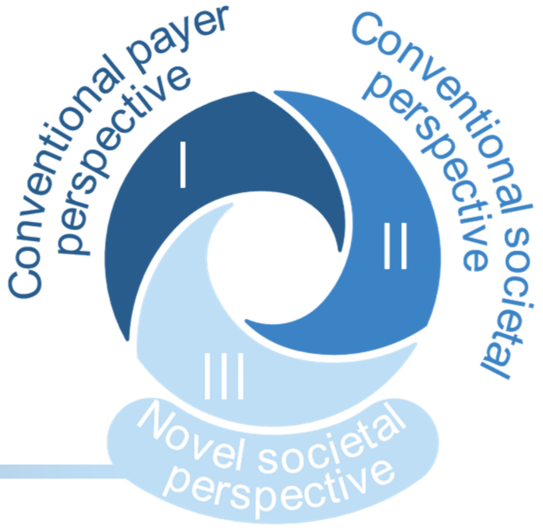

- We **prioritised 3 novel concepts** for near-term inclusion in HTA/CEA:
  - *Health systems strengthening
  - **Health equity
  - Macroeconomic gains

* Concept called "Health systems strengthening, resilience and security" in framework; ** Concept called "Social equity and ethics" in framework; CEA: cost-effectiveness analysis; HTA: Health technology assessment; VOV: Value of Vaccination

Objective and approach

OBJECTIVE

To inform decision makers, with specific examples for including vaccine benefits of health systems strengthening (HSS), health equity, and macroeconomic gains in HTA/CEA

APPROACH

- **Identify best economic methods from literature and expert working groups for valuing each concept**
- **Case studies for HSS and equity valuation, practical considerations for macroeconomic benefits**

5 Practical considerations for including key concepts in HTA/CEA

- **Literature reviews and expert working groups to determine best methods for implementing 3 concepts valuation**
- **Case study 1: Model HSS benefits with rotavirus vaccination**
- **Case study 2: Model equity benefits with meningococcal vaccination**
- **Discuss potential options for valuing macroeconomic gains within HTA/CEA**

CEA: cost-effectiveness analysis; HSS: Health systems strengthening; HTA: Health Technology Assessment

Case study 1: HSS benefits of vaccination (1)

Health Systems Strengthening (HSS) is improving:

- **Universal health coverage:** providing affordable and effective health services to all
- **Health systems resilience:** ability to adapt and function despite disturbances e.g., pandemic
- **Health systems security:** ability to protect society from global threats

Reduction in vaccine-preventable disease cases

- More HCP time, resources and budget to manage other diseases → **More efficiency, better quality of care**
- More beds and staff available to treat other diseases and reduce admission and occupancy rates → **More capacity in the health system**

The impact of vaccination on HSS can be captured by assessing its impact on health system efficiency and capacity

HCP: Healthcare professional; HSS: Health systems strengthening

Case study 1: HSS benefits of vaccination (2)

Vaccination impact on HSS can vary by 1) the existing health system capacity (e.g., constrained or sufficient, with/without winter stress periods) and 2) the type of VPD

Type of VPD

No 'winter stress' (e.g., Herpes Zoster)

'Winter stress' (e.g., Flu, RSV)

Country health system

Operates in winter at high utilization/saturation leading to crowding and waiting times (e.g., UK)

HOSPITAL

Opportunity cost approach
Useful for high utilisation capacity under stress

Has sufficient capacity & no high utilization/saturation during winter (e.g., Germany)

HOSPITAL

Vaccines can reduce pressure on health systems capacity

- Reduce 'base-line/average' health systems capacity
- Reduce 'safety stock' capacity needed

Vaccines can increase health systems efficiency

- Maintain/improve quality of care
- DRG mix and community HCP income maintained
- Stabilise personnel productivity

Several economic methods exist that could be used to quantify the benefits of vaccination on HSS e.g., opportunity cost, optimisation model, hospital capacity planning – but none could be readily incorporated into CEA.

HSS: Health systems strengthening; VPD: vaccine-preventable disease

Case study 1: HSS benefits with rotavirus vaccination (3)

The opportunity cost approach was chosen to assess HSS benefits of rotavirus vaccination

- Adapting a published CEA of infant rotavirus (RV) vaccination in the UK
- Opportunity cost: cost of health foregone by not treating the second best patient due to allocation of scarce resources to other patients

UK health system has strained capacity and winter stress periods

Peak incidence of RV gastroenteritis (RVGE) in winter

→ RV vaccination →

Significant reduction in RVGE hospitalisation

→

Pediatric wards filled with other cases

HSS benefit of RV vaccination: additional capacity to treat other patients

Opportunity cost approach can quantify how reducing RVGE frees up resources for other patients

CEA: cost-effectiveness analysis; HSS: Health systems strengthening; RVGE: rotavirus gastroenteritis

Case study 1: HSS benefits with rotavirus vaccination (4)

Rotavirus vaccination was more cost-effective with HSS benefits included: over 33,000 additional beds were available for the treatment of 5K-57K alternative patients (depending on condition)

- Methodological approach by Sandmann et al. for estimating the opportunity cost of bed-days was extended, to allow for integration into the CEA and the ICER
  - Opportunity costs evaluated from number of bed-days needed to treat RVGE patients and alternative conditions, to obtain number of bed-days and patients forgone, associated health benefits and/or expenditures.
- Public health impact:

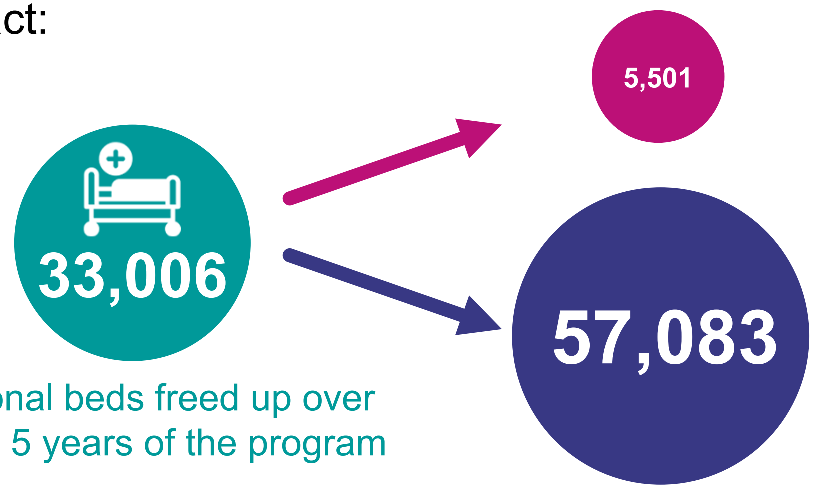

To treat (more severe) patients with chronic conditions

To treat mild URTI patients

Additional beds freed up over the first 5 years of the program

CEA: cost-effectiveness analysis; HSS: Health systems strengthening; ICER: incremental cost-effectiveness ratio; QALY: quality-adjusted life-year; URTI: upper respiratory tract infection

Case study 1: HSS benefits with rotavirus vaccination (5)

- RV vaccination could provide 20-1,461 additional QALYs for other patients benefitting from available resources, depending on the alternative hospitalisation considered

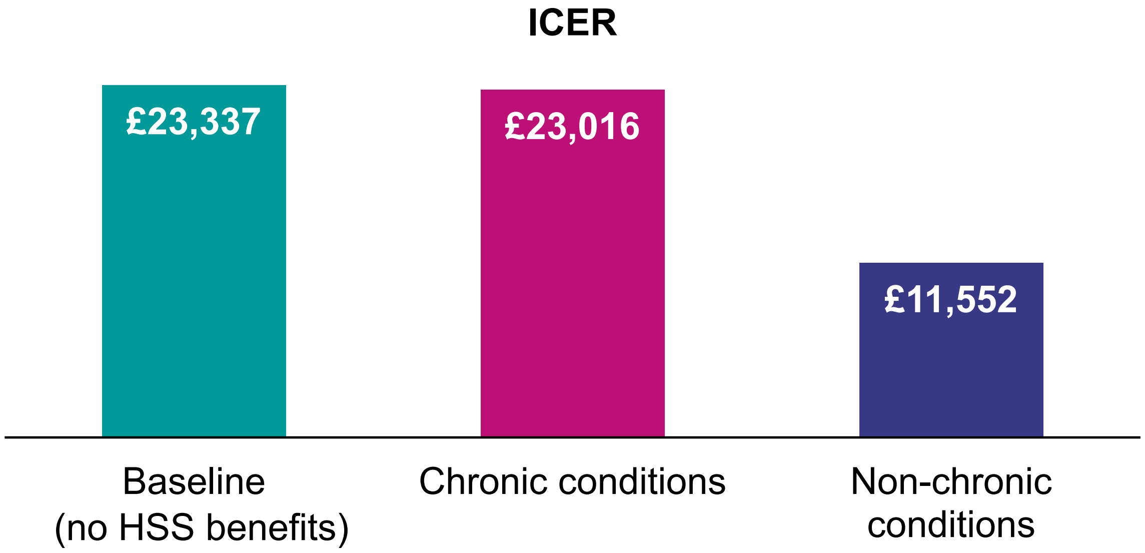

Policy implications: including HSS benefits in CEA is possible, and illustrates the additional benefits that vaccination brings to healthcare systems by freeing up resources faster to treat patients with non-vaccine preventable conditions.

CEA: cost-effectiveness analysis; HSS: Health systems strengthening; ICER: incremental cost-effectiveness ratio; QALY: quality-adjusted life-year; URTI: upper respiratory tract infection

Case study 2: Health equity benefits of vaccination

Conceptual approach to capturing health equity in HTA/CEA:

The staircase of health inequality impact guiding the health economics modelling approach

- Health inequities can be identified by disaggregating health indicators using equity stratifiers (e.g., socioeconomic status, ethnicity and geographic location)
- Disadvantaged groups may experience differences in:
  - Infectious disease incidence rates
  - Vaccine access/uptake rates
  - Health outcomes
- Promoting equity over efficiency has a health opportunity cost
  - E.g., more resources dedicated to disadvantaged groups

Social variations may arise at different steps on the staircase – and different steps may shift the health inequality impact in different directions

Health opportunity cost (*)

Difference in outcomes → **Health effect**

Difference in access to vaccines → **Uptake**

Difference in disease incidence → **Eligible population**

(*) Health loss due to intervention costs: scarce resources would otherwise be used to improve health in other ways.

Figure developed by Richard Cookson and James Love-Koh (Centre for Health Economics, University of York)

Case study 2: Health equity benefits of meningococcal vaccination

Experts agreed on a stepwise DCEA to assess vaccination impact on health equity

Objective: evaluate the potential impact of 4CMenB infant vaccination on health equity in England (retrospective analysis)

Existing CEA model[1] adapted for DCEA

- Stratify population into 5 socioeconomic subgroups using IMDQ
- Key equity-stratified inputs: carriage prevalence, incidence, vaccination coverage, utility, life expectancy, and productivity loss

CEA ➡ DCEA

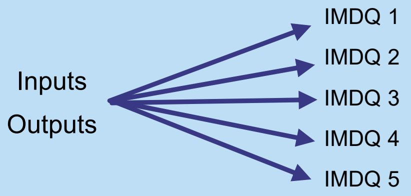

1. DISTRIBUTIONAL IMPACT

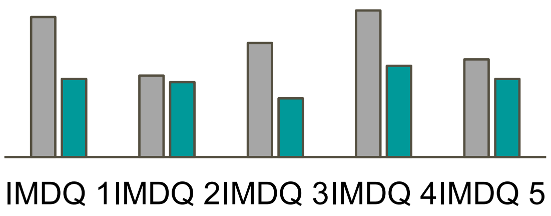

Distribution of outcomes by equity strata (IMDQ subgroups)

2. EQUITY IMPACT PLANE

Efficiency impact (e.g. net health benefit) +

NW: Win-Lose	NE: Win-Win
SW: Lose-Lose	NE: Lose-Win

- / + **Equity impact** (e.g. net reduction in health inequality index)

-

Trade-off between net health benefit (efficiency) and net equity benefit of vaccination

3. DCEA

DCEA considers **fairness** in distribution of costs and effects, and efficiency/equity **trade-offs**[2,3]

Equity accounted for with QALY weighting using inequality aversion parameters

Beck 2021[1]; Cookson 2020[2]; Cookson 2017[3]; D/CEA: Distributional cost-effectiveness analysis; IMDQ: Index of Multiple Deprivation Quintiles; QALY: quality-adjusted life-year

Case study 2: Health equity benefits of meningococcal vaccination

Results – STEP 1: distribution of health outcomes by IMDQ

- 40% of cases prevented were in the most deprived IMDQ (26% of the target population aged <5y) and 78% in the 3 most deprived IMDQs

4CMenB infant vaccination disproportionately prevented MenB cases, sequelae and deaths among more deprived groups

MenB cases prevented, by IMDQ level

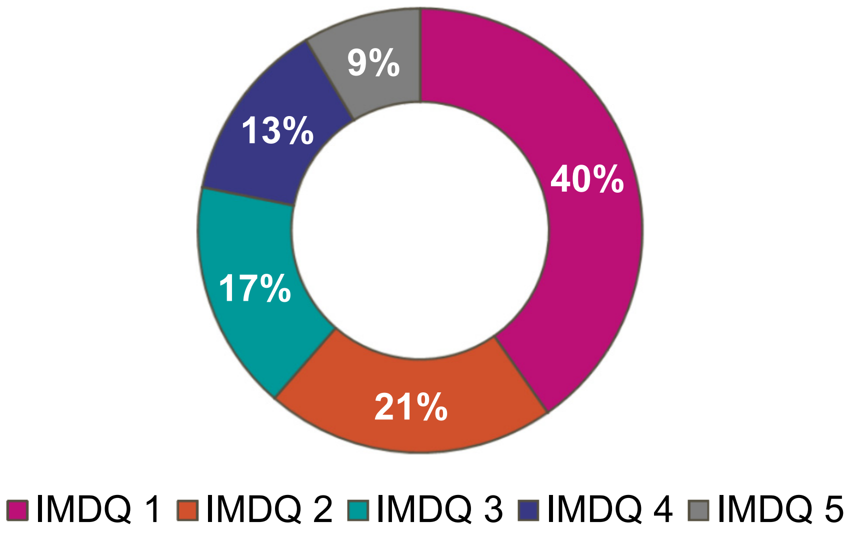

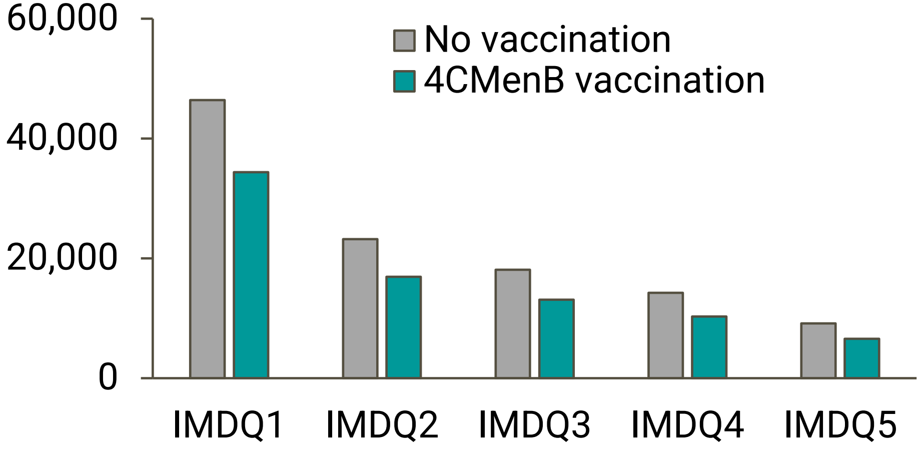

Same trends observed for other outcomes by IMDQ (e.g., for total QALY, QALY loss, incidence rate, the number of long-term sequelae, and the number of deaths related to invasive meningococcal disease)

IMDQ: Index of Multiple Deprivation Quintiles; MenB: meningococcal B; QALY: quality-adjusted life-year

Results – STEP 2: equity-efficiency impact plane (1)

Methodological considerations:

- The inequality aversion parameter is computed with total QALYs, not QALY losses
- While CEA considers average health benefits for the total population, DCEA uses the equally-distributed equivalent level of health (EDEH) taking into account **inequality aversion parameters (e.g., 10.95 [Atkinson] and 0.15 [Kolm-Pollak])**
- **EDEH were computed using two social welfare functions (SWF):**
    - Atkinson SWF: reflects relative inequality (scale-invariant) in health benefit,
    - Kolm-Pollak SWF: reflects absolute inequality (translation invariant) in health benefit

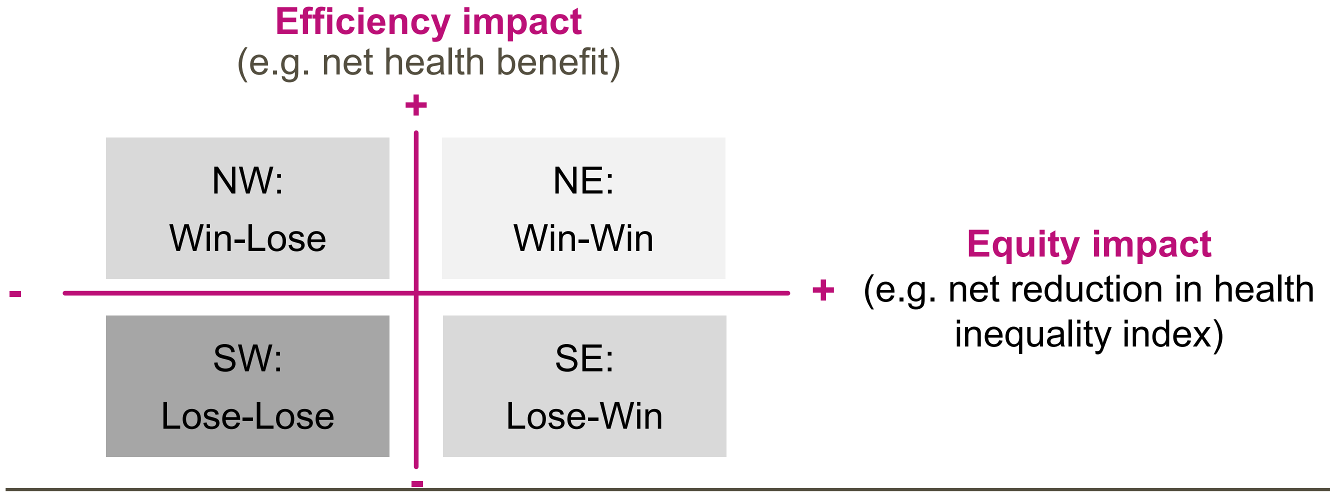

NET HEALTH BENEFIT

Health opportunity cost with vaccination, at a threshold of £20,000/QALY gained

NET EQUITY BENEFIT

Index of inequality with no vaccination vs. with vaccination

Case study 2: Health equity benefits of meningococcal vaccination

Results – STEP 2: equity-efficiency impact plane (2)

Vaccination had a positive net equity benefit, and was located in the 'win-win' quadrant from the societal perspective (reflecting both efficiency and equity benefits)

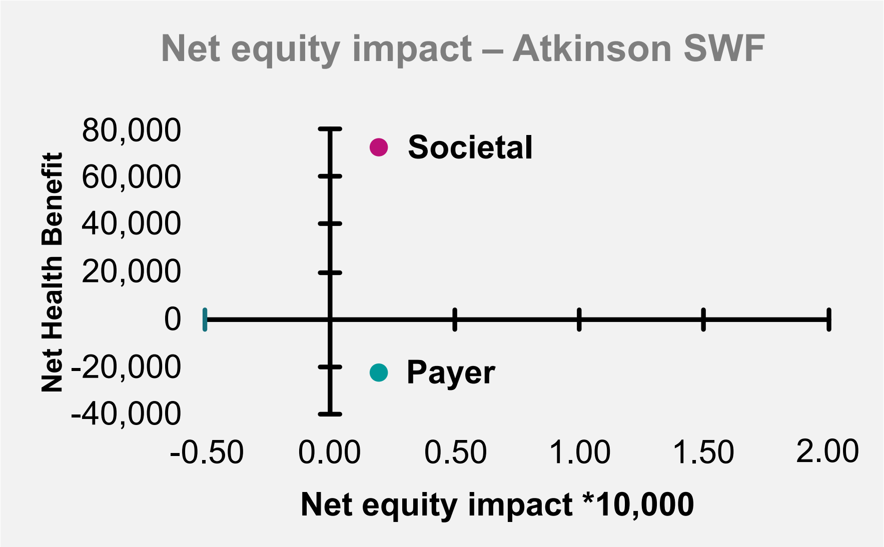

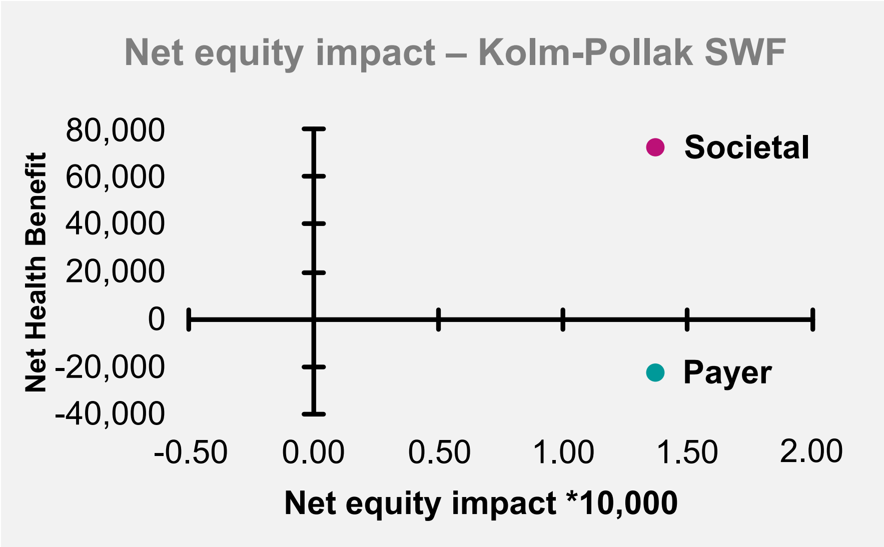

Note: the values obtained for net equity impact based on different SWF approaches are not directly comparable

The net equity benefit was robust to sensitivity analyses, varying: distribution of uptake, MenB carriage prevalence, life expectancy and utility stratified by IMDQ

IMDQ: Index of Multiple Deprivation Quintiles; SWF: Social welfare function. Note: Analysis based on original model with QALY adjustment for disease severity (QALY weight *3).

Case study 2: Health equity benefits of meningococcal vaccination

Results – STEP 3: DCEA

Analysis of equity impact: weighted QALYs to account for equity in the full DCEA

LEVEL-DEPENDENT EQUITY WEIGHTING FOR QALYS (INDIRECT WEIGHTING):

- According to the social welfare function using Atkinson's or Kolm-Pollak's inequality aversion parameters
- Equity weights for health outcomes reflect health inequality vs. IMDQ 5 (least disadvantaged group) and society's aversion to inequality
- The estimated weights for health outcomes were used to compute the equity-weighted QALYs and respective ICER
- For countries that use an ICER threshold, there is also the possibility to weight the threshold (direct weighting) e.g., as has been done for end of life treatments

Case study 2: Health equity benefits of meningococcal vaccination

Results – STEP 3: DCEA with weighted QALYs

4CMenB vaccination is more cost-effective when including the equity benefits of vaccination in DCEA

Equity-weighted QALYs resulted in **lower ICERs** from both payer and societal perspectives

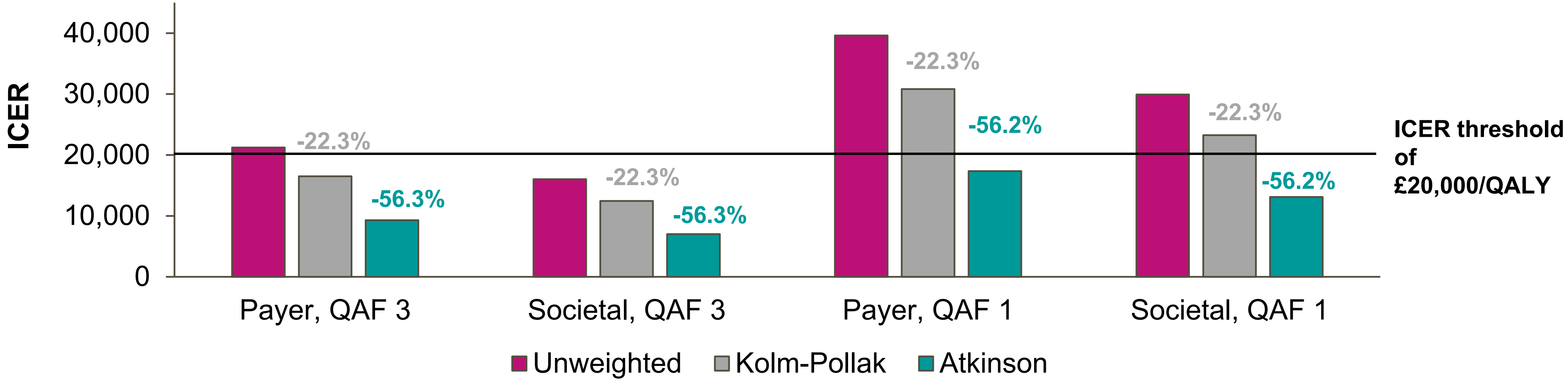

The original model included a QALY weight to account for disease severity (QAF 3)
In the QAF 1 analysis, this severity weight is removed, showing just the impact of equity weights on the ICER

DCEA: Distributional cost-effectiveness analysis; ICER: incremental cost-effectiveness ratio; QAF: quality of life adjustment factor; QALY: quality-adjusted life-year;
Note: Decision makers in England applied a QALY weight x3 to account for society's preferences to prioritise prevention of this very severe disease. QAF 1 represents removing this additional QALY weighting for severity

Case study 2: Health equity benefits of vaccination

Health equity improvements following universal 4CMenB vaccination can and should be captured in health economic evaluation

The 4CMenB infant national immunisation program **improves health equity**, by preventing disproportionately more cases in the most disadvantaged groups

- Including equity weights in DCEA reduced the ICER by 22-56%

DCEA is an important tool to demonstrate **health equity impact of vaccination**, allowing equity to be formally included in **health economic evaluation**

- Definition and alignment of health equity strata for DCEA is a key element of an analysis
- Dedicated evidence generation studies are needed to inform the equity-stratified model
- Health equity considerations should be incorporated in early stages of CEA modelling
- Further development of criteria for interpretation of equity measures could also facilitate implementation of the DCEA framework into the formal decision making process

Vaccination is a building block of universal health coverage, with a significant impact on improving health equity

Retrospective analysis demonstrated **health equity in vaccine HTA/CEA is doable, and can aid decision making - to be considered in future analyses!**

Macroeconomic benefits of vaccination

Computable General Equilibrium (CGE) models capture macroeconomics gains.

CGE models are **multi-sectoral models of the whole economy**, and have been applied to health policy interventions e.g., COVID-19, and antimicrobial resistance.

The model demonstrates the impact on gross domestic product (GDP)

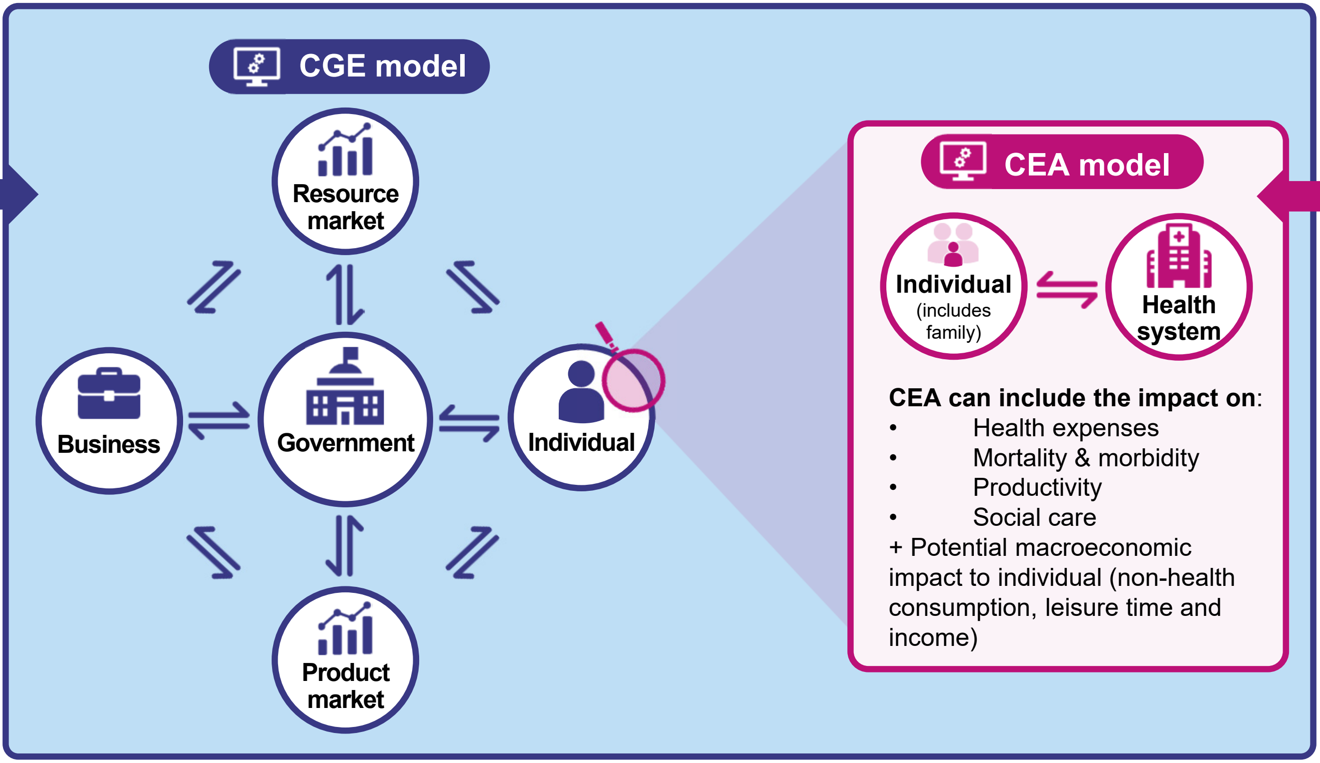

HTA/CEA uses microeconomic models with a focus on the impact of disease and interventions on **the patient and the healthcare sector**

A macroeconomic approach for vaccination **impact on economic welfare** should consider disease impact on economic components of **non-health consumption, leisure time and health status of the individual as an economic actor**

CEA: cost-effectiveness analysis; CGE: Computable general equilibrium; GDP: Gross domestic product; HTA: Health Technology Assessment

Macroeconomic benefits of vaccination

- First step to including the macroeconomic gains concept in HTA is to map commonalities in CEA and CGE models i.e., mortality, morbidity, medical and healthcare costs, productivity, social care and informal caregivers and individual behaviours.
- Disease and vaccination impacts, in both **CEA** and **CGE** models, are quantified via variations in direct health expenditure, social care, labour and productivity loss.

HTA/CEA

The impact on productivity is estimated by aggregating individual productivity/labour losses

The opportunity costs of preventing productivity losses by avoiding disease is estimated as **lost wages for the individual**

CGE

the impact on productivity loss of the entire workforce is included, and the resulting effects on other parts of the economy

- reduction in productivity
- reduction in income
- lower consumption, lower taxation, need for financial benefits
- reduced demand for goods and services, leading to reduction in supply
- changes in labour force demand, and, in turn in taxation
- impact on GDP

CEA: cost-effectiveness analysis; CGE: Computable general equilibrium; GDP: Gross domestic product; HTA: Health Technology Assessment

Macroeconomic benefits of vaccination

Adding this system perspective may help to inform the true value for money of an intervention and help to finance interventions that deliver a better return on investment from the governmental point of view

The proposal to capture macroeconomic impact in assessment and recommendation of vaccines by NITAGs and HTAs is, therefore, to complement CEA with considerations on the macroeconomic impact.

This could be done either by presenting a complementary CGE model that illustrates how the impact on productivity of a vaccine affects the broad economy, or by estimating the cost to the broad economy and including it as a fixed cost within the CEA. The latter approach also requires a reflection on how to account for a multiplier for productivity.

CEA: cost-effectiveness analysis; CGE: Computable general equilibrium; GDP: Gross domestic product; HTA: Health Technology Assessment

Conclusions (1)

The aim of this study in three parts was to broaden the valuation of vaccination programs within the context of HTA/CEA with:

1. Development of a **VoV framework**
2. Gap analysis and **identification of three priority concepts** for inclusion in economic assessments
3. Practical considerations for **assessing equity benefits, health system strengthening benefits and macroeconomic gains** due to vaccination.

D/CEA: Distributional/ cost-effectiveness analysis; HTA: Health Technology Assessment

Conclusions (2)

The case studies demonstrate that

- **expanding vaccine HTA/CEA in the near term is feasible**
- **previous positive rotavirus and meningococcal vaccination decisions are reinforced by including these broader vaccination benefits**

**Health systems strengthening**	Vaccination benefits to health systems can be assessed in HTA/CEA using the opportunity cost approach….
**Health equity**	…while the impact on equity can be assessed using DCEA.
**Macroeconomic gains**	Additional research is urgently needed to provide decision makers with more complete information on the true value of vaccines

In the past, decision makers may have underestimated the value of vaccination by not formally including assessment of novel broad societal benefits. This study paves the way to apply these proven methods in future HTA assessments.

D/CEA: Distributional/ cost-effectiveness analysis; HTA: Health Technology Assessment
