# Supplementary material for: Capturing the Value of Vaccination within Health Technology Assessment and Health Economics—Practical Considerations for Expanding Valuation by Including Key Concepts
Source: Vaccines (Basel). 2024 Jul 15;12(7):773. doi: 10.3390/vaccines12070773 (PMC11281546; doi:10.3390/vaccines12070773)
Supplement: Supplementary file 1 [file vaccines-12-00773-s001.zip › Supplementary Materials S2-S4.pdf]

Supplemental file

Manuscript title: Capturing the Value of Vaccination within Health Technology Assessment and Health Economics—Practical Considerations for Expanding Valuation by Including Key Concepts

Contents

Supplementary S2. Targeted literature reviews 3

Supplementary S3. Case study: Rotavirus vaccination and health systems strengthening 8

1. Background 8

2. Methods 8

2.1. The original analysis overview 8

2.2. Calculation tool considering opportunity costs 9

Rationale 9

Seasonality and time horizon 10

Opportunity costs 10

Calculation tool outcomes 15

2.3. Inputs 15

Seasonal distribution of RVGE cases 15

General settings and RVGE-related parameters 16

Hospitalisations occurring instead of RVGE hospitalisations 18

3. Results 20

3.1. Public health impact. Patient-equivalents and bed days foregone 21

3.2. Health benefits and associated costs 22

4. Discussion 27

Supplementary S4. Case study: MenB vaccination with distributional cost-effectiveness analysis 29

5. Background 29

6. Methods 29

6.1. The original model overview 29

6.2. Model adaptation considering health equity 32

Stratification 32

Health equity analysis 35

6.3. Model outcomes 42

6.4. Model runtime 43

6.5. Reference case inputs 44

Clinical and population inputs 44

Life expectancy 48

Utility inputs 52

Cost inputs 55

Equity inputs 57
Quality of life adjustment factor (QAF) 57
7. Results 57
7.1. Reference case results 57
Distributions 58
7.2. Health equity impact 66
Inequality index 66
7.3. Full DCEA 68
CEA summary 68
Threshold weighting (WTP) 68
QALY weighting (Total QALY) 69
7.4. Sensitivity analysis results 72
DSA and PSA results - unweighted ICER 72
DSA and PSA results - Weighted for Atkinson SWF 74
DSA and PSA results - Weighted for Kolm-Pollak SWF 76
8. Discussion 77
9. References 81

Supplementary S2. Targeted literature reviews

Targeted literature reviews were conducted for each of the three key concepts, to refine the concept definition and to identify potential methods to assess the concepts within HTA/CEA.

The following searches were conducted in PubMed on August 11, 2021.

Healthcare systems strengthening, resilience and security

("health systems"[All Fields] "health system"[All Fields] OR "health systems"[All Fields] OR "healthcare system"[All Fields] OR "healthcare systems"[All Fields])
AND
("resilience"[All Fields] OR "responsive"[All Fields] OR "responsiveness"[All Fields] OR "sustainability"[All Fields] OR "strengthening"[All Fields] OR "hss"[All Fields] OR "security"[All Fields])
AND
(((allocation, cost[MeSH Terms]) OR (allocations, cost[MeSH Terms]) OR (cost effectiveness[MeSH Terms]) OR (analyses, cost[MeSH Terms]) OR (analyses, cost benefit[MeSH Terms]) OR (analysis, cost benefit[MeSH Terms]) OR (economics[MeSH Terms]) OR ("economics, hospital"[MeSH Terms]))
OR
("vaccines"[MeSH Terms] OR "vaccination"[MeSH Terms] OR "vaccines"[All Fields] OR "vaccine"[All Fields] OR "vaccination"[All Fields] OR "immunization"[MeSH Terms] OR "immunisation"[All Fields] OR "immunization"[All Fields]))

Social equity and ethics

("equity"[All Fields] OR "equality"[All Fields] OR "ethics"[All Fields] OR "distribution"[All Fields] OR "equal"[All Fields] OR "fair"[All Fields] OR "distributional"[All Fields])
OR
((cost effectiveness[MeSH Terms]) OR (analyses, cost[MeSH Terms]) OR (analyses, cost benefit[MeSH Terms]) OR (analysis, cost benefit[MeSH Terms]))
AND
("effect"[All Fields] OR "outcomes"[All Fields] OR "access"[All Fields])
AND
("vaccines"[MeSH Terms] OR "vaccination"[MeSH Terms] OR "vaccines"[All Fields] OR "vaccine"[All Fields] OR "vaccination"[All Fields] OR "immunization"[MeSH Terms] OR "immunisation"[All Fields] OR "immunization"[All Fields])

Macroeconomic gains

("economy"[All Fields] OR "economics"[All Fields] OR "macroeconomic"[All Fields] OR "microeconomic"[All Fields] OR "fiscal"[All Fields] OR "monetary"[All Fields])
AND
(((allocation, cost[MeSH Terms]) OR (allocations, cost[MeSH Terms]) OR (cost effectiveness[MeSH Terms]) OR (analyses, cost[MeSH Terms]) OR (analyses, cost benefit[MeSH Terms]) OR (analysis, cost benefit[MeSH Terms]) OR (economics[MeSH Terms]) OR ("economics, hospital"[MeSH Terms]))
AND
("impact"[All Fields] OR "benefit"[All Fields] OR "gain"[All Fields] OR "opportunity cost"[All Fields])
AND
("vaccines"[MeSH Terms] OR "vaccination"[MeSH Terms] OR "vaccines"[All Fields] OR "vaccine"[All Fields] OR "vaccination"[All Fields] OR "immunization"[MeSH Terms] OR "immunisation"[All Fields] OR "immunization"[All Fields]))

Inclusion criteria:

- Studies published in the last 10 years in high income countries of interest

Exclusion criteria:

- Outcomes including general health systems outcomes and general costs;
- Publication types such as opinion pieces; letters to the Editor

Results

Healthcare systems strengthening, resilience and security:

N=1034 abstracts identified; N=16 articles included after title, abstract and full-text screening.

Fridell 2021 Health System Resilience: What Are We Talking About? A Scoping Review Mapping Characteristics and Keywords https://doi.org/10.15171/ijhpm.2019.71

Kutzin and Sparkes 2016. Health systems strengthening, universal health coverage, health security and resilience. https://doi.org/10.2471/blt.15.165050

Oppenheim et al. Assessing global preparedness for the next pandemic: development and application of an Epidemic Preparedness Index B 2019 https://doi.org/10.1136/bmjgh-2018-001157

Meyer D, Bishai D, Ravi SJ, et al A checklist to improve health system resilience to infectious disease outbreaks and natural hazards https://doi.org/10.1136/bmjgh-2020-002429

Lageron et al. 2015. Role of vaccination in the sustainability of healthcare systems. https://doi.org/10.3402/jmahp.v3.27043

Imai et al. A systematic review and meta-analysis of the direct epidemiological and economic effects of seasonal influenza vaccination on healthcare workers. https://doi.org/10.1371/journal.pone.0198685

Cleary et al. Economic evaluation and health systems strengthening: a review of the literature. https://doi.org/10.1093/heapol/czaa116

Chang et al. Dynamic modeling approaches to characterize the functioning of health systems: A systematic review of the literature. https://doi.org/10.1016/j.socscimed.2017.09.005

Sandmann et al. Estimating the Hospital Burden of Norovirus-Associated Gastroenteritis in England and Its Opportunity Costs for Non-admitted Patients. https://doi.org/10.1093/cid/ciy167

Standaert et al. Improvement in hospital Quality of Care (QoC) after the introduction of rotavirus vaccination: An evaluation study in Belgium. https://doi.org/10.1080/21645515.2015.1029212

Dort et al. 2018. Improving the Hospital Quality of Care during Winter Periods by Optimizing Budget Allocation Between Rotavirus Vaccination and Bed Expansion. https://doi.org/10.1007/s40258-017-0362-6

Morton et al. 2016 Decision rules for allocation of finances to health systems strengthening. https://doi.org/10.1016/j.jhealeco.2016.06.001

Verguet et al. 2019. Health system modelling research: towards a whole-health-system perspective for identifying good value for money investments in health system strengthening https://doi.org/10.1136/bmjgh-2018-001311

Social equity and ethics:

N=1034 abstracts identified; N=16 articles included after title, abstract and full-text screening

Jit et al. 2015 The broader economic impact of vaccination: reviewing and appraising the strength of evidence https://doi.org/10.1186/s12916-015-0446-9

Hinman & McKinlay 2015 Immunization equity https://doi.org/10.1016/j.amepre.2015.04.018

Cookson et al. 2021 Distributional Cost-Effectiveness Analysis Comes of Age https://doi.org/10.1016/j.jval.2020.10.001

Menkir et al. 2021 Incorporating equity in infectious disease modelling: Case study of a distributional impact framework for measles transmission https://doi.org/10.1016/j.vaccine.2021.03.023

Cookson et al. 2017 Using Cost-Effectiveness Analysis to Address Health Equity Concerns – ISPOR Task Force Report https://doi.org/10.1016/j.jval.2016.11.027

Riumallo-Herl et al. 2018 Poverty reduction and equity benefits of introducing or scaling up measles, rotavirus and pneumococcal vaccines in low-income and middle-income countries: a modelling study https://doi.org/10.1136/bmjgh-2017-000613

Pressman et al. 2021 Measuring and Promoting SARS-CoV-2 Vaccine Equity: Development of a COVID-19 Vaccine Equity Index https://doi.org/10.1089/heq.2021.0047

Gates et al. 2021 Health inequities related to vaccination: An evidence map of potentially influential factors and systematic review of interventions https://doi.org/10.1016/j.vaccine.2021.05.054

Crowcroft et al. 2012 Human papilloma virus vaccination programs reduce health inequity in most scenarios: a simulation study https://doi.org/10.1186/1471-2458-12-935

Boujaoude et al. 2018 Accounting for equity considerations in cost-effectiveness analysis: a systematic review of rotavirus vaccine in low- and middle-income countries https://doi.org/10.1186/s12962-018-0102-2

Dawkins et al. 2018 Distributional cost-effectiveness analysis in low- and middle-income countries: illustrative example of rotavirus vaccination in Ethiopia https://doi.org/10.1093/heapol/czx175

Luyten and van Hoek 2021 Integrating Alternative Social Value Judgments Into Cost-Effectiveness Analysis of Vaccines: An Application to Varicella-Zoster Virus Vaccination https://doi.org/10.1016/j.jval.2020.07.011

Menkir et al. 2021 Incorporating equity in infectious disease modeling: Case study of a distributional impact framework for measles transmission https://doi.org/10.1016/j.vaccine.2021.03.023

Portnoy et al. 2020 The impact of vaccination on gender equity: conceptual framework and human papillomavirus (HPV) vaccine case study https://doi.org/10.1186/s12939-019-1090-3

Schollin Ask et al. 2019 The Effect of Rotavirus Vaccine on Socioeconomic Differentials of Paediatric Care Due to Gastroenteritis in Swedish Infants https://doi.org/10.3390/ijerph16071095

Wateska et al. 2020 Pneumococcal Vaccination in Adults Aged ≥65 Years: Cost-Effectiveness and Health Impact in U.S. Populations https://doi.org/10.1016/j.amepre.2019.10.022

Wilson et al. 2019 Equity and impact: Ontario's infant rotavirus immunization program five years following implementation. A population-based cohort study https://doi.org/10.1016/j.vaccine.2019.01.061

Macroeconomic gains:

N=1172 abstracts identified; N=17 articles included after title, abstract and full-text screening

Sandmann et al. 2021 The potential health and economic value of SARS-CoV-2 vaccination alongside physical distancing in the UK: a transmission model-based future scenario analysis and economic evaluation https://doi.org/10.1016/S1473-3099(21)00079-7

Ruggeri et al. 2020 Estimating the fiscal impact of three vaccination strategies in Italy https://doi.org/10.1017/S0266462320000069

Thornton Snider 2019 et al. The potential impact of CAR T-cell treatment delays on society Am J Manag Care 25(8):379-386

Sevilla et al. 2018 Toward economic evaluation of the value of vaccines and other health technologies in addressing AMR https://doi.org/10.1073/pnas.1717161115

Mauskopf et al. 2018 Economic Analysis of Vaccination Programs: An ISPOR Good Practices for Outcomes Research Task Force Report https://doi.org/10.1016/j.jval.2018.08.005
Ozawa et al. 2016 Modeling The Economic Burden Of Adult Vaccine-Preventable Diseases In The United States https://doi.org/10.1377/hlthaff.2016.0462
Prager et al. 2017 Total Economic Consequences of an Influenza Outbreak in the United States https://doi.org/10.1111/risa.12625
Ahmed et al. 2018 Assessing the global poverty effects of antimicrobial resistance https://doi.org/10.1016/j.worlddev.2018.06.022
Remy et al. 215 The Economic Value of Vaccination: Why Prevention is Wealth https://doi.org/10.3402/jmahp.v3.29284
Setiawan et al. 2016 Assessment of the Broader Economic Consequences of HPV Prevention from a Government-Perspective: A Fiscal Analytic Approach https://doi.org/10.1371/journal.pone.0160707
Standaert et al. 2021 Comparing the Analysis and Results of a Modified Social Accounting Matrix Framework with Conventional Methods of Reporting Indirect Non-Medical Costs https://doi.org/10.1007/s40273-020-00978-4
Keogh Brown et al. 2020 The impact of Covid-19, associated behaviours and policies on the UK economy: A computable general equilibrium model https://doi.org/10.1016/j.ssmph.2020.100651
Connolly et al. 2020 Estimating the Fiscal Consequences of National Immunization Programs Using a "Government Perspective" Public Economic Framework https://doi.org/10.3390/vaccines8030495
Smith et al. 2009 The economy-wide impact of pandemic influenza on the UK: a computable general equilibrium modelling experiment https://doi.org/10.1136/bmj.b4571
Kotsopoulos et al. 2020 Estimating the money flow in the economy attributed to rotavirus disease and vaccination in the Netherlands using a Social Accounting Matrix (SAM) framework https://doi.org/10.1080/14737167.2020.1693269
Keogh Brown et al. 2010 The macroeconomic impact of pandemic influenza: estimates from models of the United Kingdom, France, Belgium and The Netherlands https://doi.org/10.1007/s10198-009-0210-1
Quilici et al. 2015 Role of vaccination in economic growth https://doi.org/10.3402/jmahp.v3.27044

Supplementary S3. Case study: Rotavirus vaccination and health systems strengthening

1. Background

A re-analysis of the cost-effectiveness of rotavirus vaccination in the UK was conducted, based on a previous published analysis [1], to include the benefits of vaccination for health systems, using the opportunity cost approach.

Opportunity cost is a well-established principle in economic theory that represents the forgone benefit that would have been derived from an option not chosen, in other words the value of the next best alternative use of given resources.

In the UK setting, where the health system operates at a high utilisation rate, rotavirus vaccination may decrease winter pressure and overcrowding in hospitals, as well as decrease the burden for other healthcare facilities. Thus, vaccination benefits would include not only rotavirus gastroenteritis (RVGE) cases prevented, but the freeing up of resources to treat patients with other diseases.

Opportunity costs were evaluated from the number of bed-days needed to treat patients with RVGE and alternative conditions, to obtain the number of bed-days and patients forgone, associated health benefits and/or expenditures. The methodology was mainly based on the approaches presented by Sandmann et al. [2,3], and extended to include the opportunity cost into the incremental cost-effectiveness ratio (ICER).

2. Methods

2.1. The original analysis overview

The original model was a decision tree model describing the Markov process, with specific health states that capture changes in cost and/or quality-adjusted life-years (QALYs) over time [1].

This cohort model followed a country-specific birth cohort over the average lifetime and simulated the disease process of RVGE up to the age of 5 years, in 1-month cycles. This model reflected the change in disease occurrence adjusted by subject age.

The transition probabilities in the model expressed the likelihood of developing RVGE, followed by the probabilities of subsequent events such as consulting a medical practitioner (1st or 2nd line), attending an emergency unit, hospitalisation, and death.

The model assumed that every hypothetical child in the cohort is expected to be infected at least once by the age of 5 years, with a proportion of these infections being symptomatic. Infants were assumed to be protected against rotavirus infection while being exclusively breast-fed, and the probability of a second RVGE event was lower than the probability of a first event, as infection with rotavirus has been shown to confer protection against subsequent infection [4].

Model transition probabilities were calibrated to fit published UK case numbers for community-acquired RVGE, GP consultations, accident and emergency (A&E) consultations, hospitalisations, nosocomial RVGE and deaths. For each parameter, incidence rates from published sources were multiplied by the population of children aged under 5 years in the UK (3,496,200) issued by the Office for National Statistics (ONS) (mid-2006 estimates) to give

case numbers. These were entered into the model, and subsequently calibrated using the RVGE distribution patterns to generate the transition probabilities.

Impact of RVGE on quality of life was assessed by multiplying the utility score of each health state by the amount of time spent in that health state across all the children in the vaccinated and unvaccinated cohorts.

The model estimated the costs from the perspectives of the payer and society. Depending on the chosen perspective, the model allowed to consider only direct cost (payer perspective) or direct and indirect costs (societal perspective). The direct costs included the cost of vaccination, hospitalisation, emergency visit, GP visit, cost for NHS Direct calls and prescription cost. Indirect costs considered the productivity lost by carers and transportation costs associated with the treatment of rotavirus infection. Other indirect non-medical costs such as extra nappies, over the counter medication (such as rehydration fluids), extra washing of bed linen and telephone calls were excluded due to a lack of reliable data.

2.2. Calculation tool considering opportunity costs

Rationale

Following the expert consultations, an opportunity cost approach was considered as a valid methodology to capture the value of vaccination for the health system strengthening during the annual winter-stress period in countries where the health system operates at high utilisation rates close to full capacity. Opportunity cost is a well-established principle in economics theory that represents the forgone benefit that would have been derived from an option not chosen, in other words the value of the next best alternative use of given resources [2]. For the UK setting, where health system is operating at a high utilization rate, the rotavirus vaccination may decrease winter pressure and overcrowding in hospitals, as well as decrease the burden for other healthcare facilities. Thus, the vaccination benefits for a society would include not only RVGE cases prevented, but also availability of resources to treat patients with other diseases.

A rotavirus vaccine for infants was introduced into the UK childhood immunisation schedule in July 2013 [5], which contributed to the significant reduction of the RVGE-related hospitalisations in children [6]. However, evidence from a study of Heinsbroek et al. [7] suggests that a reduction in RVGE-related paediatric hospitalisations due to vaccination did not change the overall bed occupancy, which in turn suggests that beds available were used for children with non-RVGE disease [7]. Of note, these beds are more likely to be reused within the paediatric setting (at least for the short-term perspective), and the ability to treat more paediatric patients in the hospital may be an element of value for a UK decision maker, providing monetary or non-monetary benefits.

The recent study has also shown that the number of emergency admissions in UK increases over time, particularly for infants. Thus, emergency admission rates increased between 2006/07 and 2015/16 by 23% in 0-1 year-olds, by 11% for 1-4 year-olds, and by 8% for 5-9 year-olds [8]. The increasing trend was also observed for the waiting time before A&E admission over 2012-2018 years, with the higher percentage of patients waiting for >4 h during the winter period observed for the most of years [9]. Further, waiting times for planned, elective surgeries for children in the UK are significant: median waiting time for consultant-led referral to treatment for paediatric services (X04) involving an admission to the hospital was 15.1 weeks throughout September 2021 to September 2022 [10].

This evidence suggests the existing unmet need in the access to healthcare in UK, and supports the underlying assumption applied for this analysis due to the high utilisation

setting, namely that any available bed will be likely reused for the treatment of patients with other diseases. Therefore, any hospitalized case will be associated with a certain opportunity cost for alternative patients, health system and society. For the particular setting of paediatric care in the hospital in the UK it was assumed that any acute or urgent paediatric case would receive treatment in the hospital ward with only relatively short waiting times occurring, thus the prevention of VPDs in the paediatric setting is assumed for this analysis to free up beds to treat planned, elective patients faster which are mostly awaiting surgery to improve their health [11]. During Covid 19, 63,368 children were waiting for an inpatient procedure in England [11,12]. Among these, an estimate 6,000 children were waiting for specialised surgery, 7,300 children waiting for trauma and orthopaedic surgery and 35,000 children waiting for general surgery [11,12].

The methodology for the opportunity cost estimation was based on the previous studies by Sandmann et al. [2,3]. NHS in the UK is a single payer system taxpayer funded. In this analysis, only opportunity costs incurred from the payer perspective were considered. Opportunity costs from a societal perspective (e.g., productivity losses, macro-economic considerations, carbon footprint, climate change impact, etc.) were not considered.

Seasonality and time horizon

The original model captured the impact of seasonality and child's age on the incidence of RVGE, using those for the calibration of transition probabilities for the first 5 life years of an average individual. Thus, the model does not provide explicitly the number of cases per each calendar month.

The calculation tool considered the number of hospitalisations avoided with vaccination, for community-acquired and nosocomial infections. Additionally, the number of cases expected for the peak incidence season was estimated.

Opportunity costs

For this illustrative modelling exercise, opportunity costs were evaluated from the number of bed-days needed to treat patients with RVGE and alternative conditions (referred as "other" diseases below), to obtain the number of bed-days and patients forgone, associated health benefits and/or expenditures. Several approaches were implemented as shown in Table S1, with the corresponding ICER estimation, where applicable.

Importantly, this study was mainly focused on the additional health services which could be provided to patients using resources becoming available in absence of RVGE hospitalisations, and the related health benefits (approaches 1-4, Table S1). The costs associated with providing the alternative services were also considered (approaches 5-10, Table S1), however the obtained results should be interpreted with caution, since those reflected not only benefits of the vaccination, but also the efficiency of the alternative use of resources, applying cost and health outcomes for the alternative hospitalisation, and/or the willingness-to-pay (WTP) threshold.

Table S1. Overview of approaches to value the opportunity costs and corresponding ICERs.

#	Description	Opportunity cost, per RVGE hospitalisation	ICER Vaccine vs. no vaccine	OC and ICER interpretation
**Health benefits**				
**1**	Bed-days forgone (PE)	$OC_1 = LOS_{rota}$	Not applicable	OC: Bed days which would not be available for treating patients with other diseases in the absence of vaccination. ICER: not applicable, as OC was not expressed in QALYs or monetary outcomes. This represents a 'public health impact' measure.
**2**	Patient-equivalents forgone (PE)	$OC_2 = LOS_{rota} \times \frac{OCR}{LOS_{other}}$	Not applicable	OC: Number of patients that could not be treated for other diseases in the absence of rotavirus vaccination. ICER: not applicable, as OC was not expressed in QALYs or monetary outcomes. This represents a 'public health impact' measure.
**3**	Gross health benefit forgone for the second-best patient-equivalents (PE)	$OC_3 = LOS_{rota} \times \frac{H_{other}}{LOS_{other}}$	$\frac{C_{vac} - C_{no\,vac}}{H_{vac} - (H_{no\,vac} - N_{rota} \times OC_3)}$	OC: Gross health benefit that could not be obtained for the alternative patient in the absence of rotavirus vaccination. ICER: Total amount of additional QALYs for all avoidable RVGE hospitalisations was subtracted from the total QALYs gained in the arm without vaccination, to reflect the lost treatment opportunity.
**4**	Health benefit forgone for the best alternative use (TE)	$OC_4 = C_{rota} \times \frac{1}{\gamma}$	$\frac{C_{vac} - (C_{no\,vac} - N_{rota} \times C_{rota})}{H_{vac} - (H_{no\,vac} - N_{rota} \times OC_4)}$	OC: Amount of health (QALYs) that could not be provided in absence of vaccination due to the costs incurred for RVGE hospitalisation at a defined willingness to pay (WTP) threshold. ICER: The additional QALY loss for all avoidable RVGE hospitalisations was accounted for the arm without vaccination, due to the lost treatment opportunity. It was considered that the alternative use of the resources available with rotavirus vaccination would be paid equally to RVGE hospitalisations. As this expense would not occur in the absence of vaccination, it was subtracted from the total costs of the no vaccination strategy.
**Health benefits and associated costs**				

**5**	Gross expenditure saved on the second-best* patient-equivalents (PE)	$OC_5 = LOS_{rota} \times \frac{C_{other}}{LOS_{other}}$	$\frac{C_{vac} - (C_{no\ vac} - N_{rota} \times (OC_5 - C_{rota}))}{H_{vac} - H_{no\ vac}}$	OC: Expenditure saved due to alternative hospitalisations would be offset by the expenditure spent for RVGE hospitalisations.<br>ICER: The difference between expenditure saved for alternative patient and expenditure incurred for RVGE patient was computed, multiplied by the total number of avoided RVGE hospitalisations, and then subtracted from the total costs of the arm without vaccination.
**6**	Net monetary benefit forgone for the second-best patient-equivalents (PE)	$OC_6 = LOS_{rota} \times \frac{(H_{other} \times \gamma - C_{other})}{LOS_{other}}$	$\frac{C_{vac} - (C_{no\ vac} + N_{rota} \times OC_6)}{H_{vac} - H_{no\ vac}}$	OC: Net monetary benefit not obtained for the alternative patient in the absence of rotavirus vaccination.<br>ICER: The net monetary benefit lost for all avoidable RVGE hospitalisations was considered as an additional cost associated with the no vaccination strategy.
**7**	Expenditure incurred + net monetary benefit forgone (PE)	$OC_7 = C_{rota} + LOS_{rota} \times \frac{(H_{other} \times \gamma - C_{other})}{LOS_{other}}$	$\frac{C_{vac} - (C_{no\ vac} + N_{rota} \times (OC_7 - C_{rota}))\ **}{H_{vac} - H_{no\ vac}}$	OC: Net monetary benefit not obtained for the alternative patient in the absence of rotavirus vaccination (as for OC_6), when added to the costs incurred for the treatment of RVGE.<br>ICER: Similarly to approach 6, however the cost of RVGE hospitalisation added on the opportunity cost side should be subtracted when the ICER was computed, to avoid double-counting (this cost was already accounted in $C_{no\ vac}$). ICER estimate was equal to obtained in the approach 6.
**8**	Gross monetary benefit forgone for the second-best patient-equivalents (PE)	$OC_8 = LOS_{rota} \times \frac{H_{other} \times \gamma}{LOS_{other}}$	$\frac{C_{vac} - (C_{no\ vac} + N_{rota} \times (OC_8 - C_{rota}))}{H_{vac} - H_{no\ vac}}$	OC: Gross monetary benefit not obtained for the alternative patient in the absence of rotavirus vaccination.<br>ICER: The difference between the gross monetary benefit expected for alternative patient and expenditure incurred for RVGE patient was computed, multiplied by the total number of avoided RVGE hospitalisations, and then added to the total costs of the no vaccination strategy.
**9**	Net health benefit forgone for the second-best	$OC_9 = LOS_{rota} \times \frac{(H_{other} - C_{other}/\gamma)}{LOS_{other}}$	$\frac{C_{vac} - C_{no\ vac}}{H_{vac} - (H_{no\ vac} - N_{rota} \times OC_9)}$	OC: Net health benefit not obtained for the alternative patient in the absence of rotavirus vaccination.<br>ICER: net health benefit lost for all avoidable RVGE

	patient-equivalents (PE)			hospitalisations was considered as an additional health loss associated with the no vaccination strategy.
**10**	Gross expenditure saved and gross health benefit forgone on the second-best patient-equivalents (PE)	OC_3 *as above*<br>OC_5 *as above*	$\frac{C_{vac} - (C_{no\ vac} - N_{rota} \times OC_5)}{H_{vac} - (H_{no\ vac} - N_{rota} \times OC_3)}$	<u>OC:</u> Gross health benefit not obtained and gross expenditure saved for the treatment of alternative patient in the absence of rotavirus vaccination (computed as OC_3 and OC_5 above).<br><u>ICER:</u> Gross health and monetary benefits lost for all avoidable RVGE hospitalisations were considered as additional health lost and expenditure saved with the no vaccination strategy, respectively.

OC: opportunity costs, LOS: length of stay in days, OCR: occupancy rate, C: costs incurred, H: health benefits (e.g., QALY), N: number of hospitalisations avoided, γ: cost-effectiveness threshold, PE: patient-equivalent approach TE: treatment-equivalent approach, rota: hospitalisation due to RVGE, other: hospitalisation due to other causes, displaced by RVGE, vac: with vaccination, no vac: without vaccination,

* “second-best patient” refers to the alternative patient with “other” diseases assumed to be hospitalized instead of the patient with RVGE.

C_{rota} should be subtracted for ICER calculation, to avoid double-counting, as it is accounted in the opportunity cost. ICER will be equal to the estimate from the approach 6.

The health benefit parameter (H) represented the 'incremental' health benefit gained due to hospitalisation vs. no hospitalisation which could be, as laid out by Sandmann et al. 2018 (appendix) [3], resulting from deaths avoided, severe complications avoided with long-term lower quality of life, and shorter duration of a health state with lower quality of life (i.e., faster recovery). The costs parameter related to hospitalisation due to other causes (C_{other}) is the incremental cost of the additional hospitalisation which may be the actual cost of the hospitalisation or other services needed when waiting for hospitalisation, e.g. an additional GP or A&E visit, outpatient procedures or medications. Even though not in the scope of this exercise, it would be worth to consider the potential productivity losses and loss in leisure time associated with this, for the further adaptations.

The same considerations were applied for hospitalisation due to community-acquired and nosocomial infections, and the respective opportunity costs per a single hospitalisation were multiplied by the number of avoided hospitalisations by type, and further summed up to compute the total opportunity cost related to all RVGE hospitalisations.

The previous study by Sandmann et al. [2] proposed several methodologies for the estimation of opportunity costs, representing patient-equivalent and treatment-equivalent approaches:

- Patient-equivalents (PE) were calculated in terms of the number of alternative patients that could have been treated using the same resources, e.g., bed-days, differently. Frequently, the alternative patient was (implicitly) approximated by the average patient population likely to occupy that bed.
- Treatment-equivalents (TE) were calculated in terms of the number of alternative treatments that could have been paid for using the same expenditure incurred differently. Resources such as beds are hence assumed to be monetised and the money spent elsewhere within health system. This group of approaches could be less applicable for the UK setting and RVGE occurring during the winter stress, however it was also presented for illustrative purpose.

In this study patient-equivalent approaches (referred as OC_1-OC_3 and OC_5-OC_{10}) were presented, in order to keep consistency with the underlying assumption on the maintained hospital occupancy rate regardless of the rotavirus vaccination [7]. An alternative treatment-equivalent approach (OC_4) was also explored. As an extension of the methodology proposed by Sandmann et al.[2,3], this study considered opportunity cost expressed in monetary outcomes and health outcomes for the estimation of ICER, where applicable. Additionally, two approaches were implemented in this study: OC_1, to capture the number of bed-days forgone with RVGE hospitalisations to better demonstrate the public health impact, and OC_{10}, to capture opportunity cost expressed in both types of outcomes within a single ICER.

Several approaches proposed by Sandmann et al. were not applied for the planned analysis: (1) approaches which considered the revenue for the healthcare provider were omitted, as the healthcare provider perspective was out of scope for this adaptation which was focused on the healthcare payer perspective; (2) treatment-equivalent approaches which consider health benefit for the second-best treatment were omitted (apart from one approach, OC_4, taken for illustrative purpose) as such benefit obtained elsewhere within the health system beyond hospitalisations would be difficult to define and quantify.

The list of "other" diseases or admissions, displaced by RVGE, could include a considerable number of infectious and non-infectious pathologies. Based on expert opinion, however, it was considered that in general any acute or urgent paediatric case should be assumed to receive treatment in the hospital ward with only relatively short waiting times occurring. Thus, freed up beds resulting from displacing RVGE should from a conceptual point of view

for this case study be assumed to allow for admission of patients waiting for referral based inpatient procedures. A dedicated study would be needed to investigate this aspect and determine the most appropriate choice of ‘other’ paediatric admissions displaced by RVGE, which however is out of scope of the planned calculation tool. To allow for capture of potential different aspects of ‘other’ paediatric admissions, several scenarios were defined to reflect the possible range of alternative conditions which could be treated in absence of RVGE hospitalisations.

For this pilot study, only bed-days were used for the opportunity cost estimation. The study by Thomas et al. [13] showed that ~70% of direct savings for NHS associated with rotavirus vaccination came from reduced hospitalisation costs. Therefore, it could be reasonably assumed that the hospitalisation-related savings for opportunity costs for rotavirus vaccination could represent the substantial proportion of the total opportunity costs.

For future analyses, the opportunity cost could be also computed for other relevant healthcare resource usage beyond hospitalisations (e.g., NHS Direct calls, GP consultations, A&E visits etc.), and LOS in the formulas above could be replaced by units of the corresponding resource used. Further, the total opportunity cost could be estimated as a sum of opportunity costs for specific resources (e.g., NHS Direct calls, GP consultations, A&E visits etc.). However, the interpretation of the total opportunity cost would largely depend on the completeness and consistency of the data sources used to inform computations for each resource. The described opportunity cost approach is relevant for health systems operating at capacity as it assumes that the resource becoming available in absence of rotavirus infections (resulting from vaccination) would be utilized by paediatric patients with other diseases.

Calculation tool outcomes

The calculation tool estimated the following outcomes:

- Opportunity costs of avoided hospitalisation
    - Per one hospitalisation avoided for community-acquired infection.
    - Per one hospitalisation avoided for nosocomial infection.
    - Per total hospitalisations avoided.
- ICER adjusted for opportunity costs (where applicable).

Opportunity costs per total hospitalisations avoided and corresponding ICERs are provided in this report.

2.3. Inputs

Seasonal distribution of RVGE cases

The seasonal distribution of rotavirus cases in the UK is reported by NHS public reports [5,14] and the surveillance study by Hungerford et al. [15].

Both studies consistently reported that before introduction of the vaccination programme, the number of confirmed cases started to rise in late December, peaked around February, and decreased in June. The study by Hungerford et al. [6] suggested that **95%** (5,014/5,272) of rotavirus cases observed in the UK in 2007/2008-2012/2013 occurred during weeks 1–25 of the year, which was considered as the peak incidence season. This estimate was used for the planned analyses (hereinafter referred as “6-months timeframe”).

Additionally, an alternative estimate was included, to explore the impact of an assumption of a shorter duration of the peak incidence season (March to May). The proportion of cases

occurring during this period was estimated at **64%**, based on NHS public reports by month [16], for 2007/2008-2012/2013 seasons, (hereinafter referred as "3-months timeframe").

General settings and RVGE-related parameters

Input parameters for RVGE and vaccine were derived as follows:

- RVGE and vaccine-related parameters (population number, C_{rota}, LOS_{rota}, N_{rota}, C_{vac}, $C_{no\ vac}$, H_{vac}, and $H_{no\ vac}$ were based on the previous study by Martin et al. [1] conducted in the UK.
- The study by Martin et al. reported health and cost outcomes being discounted at an annual rate of 3.5%. Therefore, all estimates derived from this source were consistently discounted and no additional adjustment was applied specifically for the opportunity costs in this study. The alternative hospitalisations were expected to occur in the same year as RVGE hospitalisations were avoided, thus the impact of discounting should be accounted for the same period, and it seems sufficient to apply it only for the analysis inputs (i.e., to adopt the estimates reported by Martin et al. [1]). It is unclear if this approach was associated with any bias, however as the majority of hospitalisations are expected during the first 2 years after the child's birth, this potential bias is not expected to be significant.
- OCR was assumed to be 90%, considering that seasonal outbreaks lead to the crowding of paediatric wards and intensive care units (ICU) as provision of paediatric ward and ICU capacity is mostly focused on the periods of the highest utilisation. This assumption is supported by the expert opinion provided during the previous advisory board series as well as the available literature for the UK [7,17].

Table S2. Opportunity cost parameters - general settings and RVGE-related parameters.

Description	Parameter name	Value	Source	Comment
**General settings**				
**Bed occupancy rate**	OCR	90%	Expert opinion	-
**Proportion of cases occurring in peak incidence season**<br>**● 6-months timeframe**<br>**● 3-months timeframe**	-	● 95%<br>● 64%	● [6]<br>● assumption, [16]	● [6] Data for the UK<br>● [16] Data for England and Wales
**Cost-effectiveness threshold**	γ	£ 20,000	[1]	[1] Data for the UK
**Population (birth cohort)**	-	716,000	[1]	[1] Data for the UK

Description	Parameter name	Value	Source	Comment
**RVGE**				
**Cost of hospitalisation, gastroenteritis cases *** **● community-acquired** **● nosocomial**	C_{rota}	● £ 772 ● £ 662	[1]	[1] Data for the UK
**LOS, RVGE** **● community-acquired** **● nosocomial**	LOS_{rota}	● 2 ● 2	[1]	[1] Data for the UK
**Cost-effectiveness outcomes, Rotarix vaccination vs. no vaccination**				
**Incremental costs of hospitalisation** **● community-acquired** **● nosocomial**	-	● £ 9,938,349 ● £ 2,977,772	[1]	[1] Data for the UK N_{rota} (number of hospitalisations avoided with vaccination, in a peak incidence season) will be computed using the reported total costs for hospitalisation, cost for a single hospitalisation (C_{rota}), and proportion of cases occurring in peak incidence season.
**Cost incurred in a world with vaccination**	C_{vac}	£ 58,220,236	[1]	[1] Data for the UK
**Cost incurred in a world without vaccination**	$C_{no\ vac}$	£ 24,801,080	[1]	[1] Data for the UK
**QALY per child in a world with vaccination**	-	25.981	[1]	[1] Data for the UK H_{vac} (QALY in a world with vaccination) will be computed using the reported estimate per child and the size of the starting population (birth cohort).
**QALY per child in a world without vaccination**	-	25.979	[1]	[1] Data for the UK $H_{no\ vac}$ (QALY in a world without vaccination) will be computed using the reported estimate per child and the size of the starting population (birth cohort).

OCR – occupancy rate,
LOS – length of stay in days,
γ – cost-effectiveness threshold,
C – costs incurred,
H – health benefits,
N – number of hospitalisations avoided,
rota – hospitalisation due to RVGE,
vac – world with vaccination,
no vac – world without vaccination

* – This study aimed to represent an extension of the previous cost-effectiveness study by Martin et al. [1] for the UK, reflecting the opportunity costs assessment for the period of the vaccine introduction. Therefore, to keep consistency with the previously reported cost-effectiveness outcomes [1], costs were not inflated.

Hospitalisations occurring instead of RVGE hospitalisations

There is considerable uncertainty regarding the nature of other diseases, for which a bed becoming available would be used, and it could be reasonably assumed that those were occupied by patients with different conditions.

Importantly, the opportunity cost concept routinely considers a value of the second-best alternative use, chosen among several alternatives using the net benefit estimates [2,3]. However, it is not clear whether such net benefit estimates should be derived using the cost-effectiveness threshold of £20,000-30,000/QALY gained used by NICE for health technology assessment (HTA). Indeed, this WTP threshold may not be suitable for valuing hospital interventions or admissions, especially in the context of emergency and/or paediatric services considering also 'the rule of rescue'. Furthermore, it is likely that in practice the decision for providing health services or admitting a patient will be based on the clinical need rather than a cost-effectiveness of these services [18-20].

A range of 6 scenarios considering alternative hospitalisations were explored, with the respective estimates for costs, LOS and QALY gain associated with hospitalisation.

Input values for the scenario analyses are presented in Table S3, detailed description of each scenario is provided below.

Table S3. Opportunity cost parameters – hospitalisations occurring instead of RVGE hospitalisations.

Parameter	Scenario 1: RTT admitted	Scenario 2: URTI	Scenario 3: URTI mild	Scenario 4: URTI severe	Scenario 5: Non-gastroenteritis cases, with chronic conditions	Scenario 6: Non-gastroenteritis cases, without chronic conditions
**Cost of hospitalisation, non-gastroenteritis cases (C_{other})**	£ 1,364	£ 409	£ 307	£ 511	£ 1,270	£ 1,270
**LOS, non-gastroenteritis cases (LOS_{other})**	1.2	1.0	0.5	2.1	5.4	3.3
**QALY gain associated with hospitalisation, non-gastroenteritis cases (H_{other})**	0.0047	0.0014	0.0010	0.0017	0.239	0.002

C – costs incurred,
LOS – length of stay in days,
RTT – referral to treatment with hospital admission (e.g., referral for general surgery)
H – health benefits,
other – hospitalisation due to other causes, displaced by RVG

Scenario 1 considered children waiting for referral-based treatments (RTT) in hospital such as elective surgeries as a proxy for alternative conditions taking into consideration the underlying assumptions of urgent paediatric cases to be treated without significant delay in the hospital. As proxy for such a scenario and considering the conceptual nature of this case study, it was assumed that displacement of RVGE would lead to a total reduction in waiting time for RTT, so prevention of an average 15.1 weeks of waiting time [21] reflective of the average median wait time for paediatric procedures in the hospital from September 2021 to September 2022 (data on wait time for inpatient paediatric RTT procedures were not available before 2020). In approximation of the loss of quality of life during waiting time and the improvement of quality of life due to surgery, a disutility of 0.0162 based on Suhonen et al. 2008 [22] was assumed. With regards to cost, herniotomies were assumed a proxies for the RTTs patients to be admitted instead of patients with RVGE as observed as most common procedure among general paediatric surgeries in less than 5 year-olds in the UK [23]. Cost and average length of stay of herniotomies were estimated based on herniotomy related surgeries (currency codes: FZ17D, FZ18D, FZ19Z, FZ39F) statistics available in the National Schedule of Reference Costs 2009-10 for NHS Trusts [24] and applying a deflation of costs to 2005/2006 using price & pay index.

Scenarios 2-4 considered Upper Respiratory Tract Infection (URTI) as a proxy for alternative conditions since the available data suggest that the peak incidence seasons for rotavirus and URTI largely overlap, and URTI is reported as one of the most common causes for hospitalisation [8]. Thus, it could be assumed that a considerable number of patients with URTI can be admitted instead of patients with RVGE. Variation in the input parameters was applied to address the uncertainty around the severity of the admitted URTI case.

Scenario 2 considered URTI as a proxy for alternative conditions with inputs estimated for severe cases. Costs of hospitalisation were based on the data from 2015/2016 for PD11B and PD11C [25], deflated to 2005/2006 cost year according to pay & prices index 2015 and 2016 [16,26]. Costs for PD11A were not considered, as patients with high score (4+) are assumed to be hospitalized regardless of the occupancy rate. LOS of 1 day was assumed instead of 0.5 days reported by HRG for PA11Z [27], as the latest would be difficult to interpret, and a major part of costs would be likely incurred at the admission. QALY gain associated with hospitalisation was calculated as difference between cases with complications lasting for one day vs. two days [28], assuming that timely hospitalisation prevented aggravation of the condition.

Scenario 3 considered lower bounds for URTI parameters. Costs and QALY gain associated with hospitalisation were calculated as -25% of the values used in the Scenario 1. LOS was set to 0.5 as reported for URTI infections by HRG (PA11Z) [27].

Scenario 4 considered higher bounds for URTI parameters. Costs and QALY gain associated with hospitalisation were calculated as +25% of the values used in the Scenario 1. LOS was set to 2.1 as reported for upper respiratory tract disorders with LOS of 1 day or more (PA65B, PA65C) [27].

Scenarios 5 and 6 considered non-gastroenteritis patients: with chronic conditions, and without chronic or life-threatening conditions respectively, to address the uncertainty around the severity of the non-gastroenteritis admitted case. Of note, these scenarios could plausibly represent the "second-best" alternative use since the decision space includes only two mutually exclusive options (gastroenteritis and non-gastroenteritis hospitalisation). The important limitation of these scenarios is the lack of data for paediatric patients for LOS and QALY gained, and uncertainty regarding the distribution of costs for severe and mild cases.

Scenario 5 was based on the data provided by Sandmann et al. [3]. Cost of alternative hospitalisation was derived as mean NHS reference costs of non-gastroenteritis in England in 2015/2016, activity-weighted, excluding HRGs FZ36 and PF21 (following the approach reported by Sandmann et al. [3]), and additionally excluding codes related to adults and children over 5 years to adapt the estimate to the paediatric setting. The obtained value was deflated to 2005/2006 cost year according to pay & prices index 2015 and 2016 [16,26]. LOS and QALY gain were derived from the data for patients with chronic conditions with the mean age of 61.7 years [3].

Scenario 6 was based on the data provided by Sandmann et al. [3]. Cost of alternative hospitalisation was equal to the value used in Scenario 4, as this estimate covers "average" non-gastroenteritis hospitalisation, and it is not clear how to quantify the impact of chronic condition on the cost of stay, due to broad definitions applied (for "chronic condition" and "non gastroenteritis"). LOS and QALY gain were derived from the data for patients without chronic or life-threatening conditions with the mean age of 48.7 years [3]. Thus, this scenario explored uncertainty around LOS and QALY for non-gastroenteritis case, assuming the average cost of hospitalisation.

3. Results

This study considered that maximizing the health benefit is a priority for decision-makers. Therefore, approaches 1-4 (

Table S1) were proposed as the most relevant for the estimation of the opportunity costs associated with RVGE hospitalisations in the absence of rotavirus vaccine. The introduction of vaccination could be associated with the higher availability of hospital beds, which could be used for the treatment of patients with different diseases (estimated with approaches 1 and 2 respectively; 'public health impact' estimation). The associated health benefits (approach 3) as well as other resources becoming available (approach 4) were expressed as opportunity cost and then considered for the ICER estimation, as these additional benefits could be seen as an element of the value of vaccination for health system.

3.1. Public health impact. Patient-equivalents and bed days foregone

Table S4 and

Table S5 below provide the number of bed-days (approach 1) and patient-equivalents forgone (approach 2) due to the RVGE hospitalisation. The results obtained showed that rotavirus vaccination was associated with an additional 33,006 beds becoming available, over the first 5 years of life of the vaccinated cohort, when the 6-months timeframe for the peak incidence season was considered. These bed-days could be reused for the treatment of 5,501 to 57,083 other patients, which otherwise would not be admitted (or would be admitted after the certain waiting time). The number of alternative patients was the lowest for a scenario 4, considering non-gastroenteritis hospitalisation in patients with chronic conditions, and highest for a scenario 2, considering mild URTI as an alternative hospitalisation, which was in line with the length of stay for the respective disease.

When the 3-months timeframe for the peak incidence season was considered, all estimated outcomes were lower for about 30% comparing to the 6-months timeframe.

Table S4. Results – Opportunity costs. Patient-equivalents and bed days forgone (6-months timeframe for the peak incidence season).

#	Approach	Scenario 1: RTT admitted	Scenario 2: URTI	Scenario 3: URTI mild	Scenario 4: URTI severe	Scenario 5: Non-gastroenteritis cases, with chronic conditions	Scenario 6: Non-gastroenteritis cases, without chronic conditions
	**Parameters for non-gastroenteritis cases:**<br>• Cost of hospitalisation (C_{other})<br>• LOS (LOS_{other})<br>• QALY gain associated with hospitalisation (H_{other})	• £ 1,364<br>• 1.2<br>• 0.0047	• £ 409<br>• 1.0<br>• 0.0014	• £ 307<br>• 0.5<br>• 0.0010	• £ 511<br>• 2.1<br>• 0.0017	• £ 1,270<br>• 5.4<br>• 0.239	• £ 1,270<br>• 3.3<br>• 0.002
		Opportunity costs					
1	Bed-days forgone (PE)				33,006		
2	Patient-equivalents forgone (PE)	25,168	29,706	57,083	14,165	5,501	9,002

Table S5. Results – Opportunity costs. Patient-equivalents and bed days forgone (3-months timeframe for the peak incidence season).

#	Approach	Scenario 1: RTT admitted	Scenario 2: URTI	Scenario 3: URTI mild	Scenario 4: URTI severe	Scenario 5: Non-gastroenteritis cases, with chronic conditions	Scenario 6: Non-gastroenteritis cases, without chronic conditions
	**Parameters for non-gastroenteritis cases:**<br>• Cost of hospitalisation (C_{other})<br>• LOS (LOS_{other})<br>• QALY gain associated with hospitalisation (H_{other})	• £ 1,364<br>• 1.2<br>• 0.0047	• £ 409<br>• 1.0<br>• 0.0014	• £ 307<br>• 0.5<br>• 0.0010	• £ 511<br>• 2.1<br>• 0.0017	• £ 1,270<br>• 5.4<br>• 0.239	• £ 1,270<br>• 3.3<br>• 0.002
	Opportunity costs						
1	Bed-days forgone (PE)	22,070					
2	Patient-equivalents forgone (PE)	16,829	19,863	38,169	9,472	3,678	6,019

3.2. Health benefits and associated costs

Table S6 and

Table S7 below provide the estimates for the opportunity costs associated with RVGE hospitalisations avoided over the first 5 years of life of the vaccinated cohort, expressed in health and/or costs outcomes, and the corresponding ICERs. The baseline ICER which did not account for any opportunity costs was estimated at £ 23,337/QALY gained, considering incremental costs and QALYs derived from Martin et al. [1]

The results obtained with 6-months timeframe for the peak incidence season for each of the considered approaches should be interpreted as follows:

- Approach 3. The rotavirus vaccination could provide the additional gross health benefit of 20-1,461 QALY gained for patients admitted to a hospital using beds becoming available due to avoided RVGE hospitalisations. ICER considering this additional health benefit was estimated at £11,552-£23,016 /QALY, which was lower than the baseline ICER for all scenarios.
- Approach 4. The rotavirus vaccination could provide the additional health benefit of 614 QALY gained for patients which can be treated using the funds saved for avoided RVGE hospitalisations. ICER considering this additional health benefit was estimated at £22,336 /QALY, which was lower than the baseline ICER. The opportunity cost and ICER estimates were the same in all scenarios, as this approach did not specify how the newly available resources are used.
- Approach 5. The rotavirus vaccination could result in the additional gross expenditure of £7,760,420-£38,134,624 for the treatment of patients admitted to a hospital using beds

becoming available in absence of RVGE hospitalisations. If the alternative treatment was less costly, comparing to RVGE hospitalisation, ICER considering this opportunity cost was lower than the baseline estimate (for scenarios 4 and 5); and vice versa, the ICER was higher when more costly treatments were considered (scenarios 1, 2 , 3 and 6).

- Approach 6. The rotavirus vaccination could be associated with positive or negative net monetary benefit of the alternative hospitalisations. Positive net health benefit was estimated for the scenario 5, as the considered alternative hospitalisation could be "cost-effective", if NHS cost-effectiveness threshold would be applicable for hospital services (ICER for this alternative hospitalisation was estimated at £1,270 / 0.239 = £5,312 /QALY gained). ICER for rotavirus vaccination vs. no vaccination was lower than the baseline estimate, when opportunity costs were considered in the scenario 5. For the other scenarios, negative net monetary benefit was expected, therefore ICER considering this opportunity cost was higher than the baseline estimate.
- Approach 7. As shown for the Approach 6, the rotavirus vaccination could be associated with positive or negative net monetary benefit of the alternative hospitalisations. Additionally, opportunity cost estimate could include the expenditures associated with RVGE hospitalisation (as those would be still incurred in absence of vaccination). With this approach, estimated opportunity costs increased for all scenarios, when compared to approach 6. However, the cost of RVGE hospitalisation added at the opportunity cost side should be subtracted when the ICER considering this opportunity cost was computed, to avoid double-counting (at this cost was already accounted in $C_{no\ vac}$). Therefore, ICER estimates remained the same as in the approach 6.
- Approach 8. The rotavirus vaccination could provide the additional gross monetary benefit estimated from the health benefit for patients admitted to a hospital using beds becoming available in absence of RVGE hospitalisations (when NHS cost-effectiveness threshold was considered). Gross monetary benefit varied in a range £400,074-£29,216,549, depending on the scenario settings. Similarly to approach 6, the ICER considering this opportunity costs was lower than the baseline estimate only for the scenario 5, as the considered alternative hospitalisation could be "cost-effective".
- Approach 9. The rotavirus vaccination could be associated with positive or negative net health benefit of the alternative hospitalisations. Similarly to approach 6, positive net health benefit estimated for the scenario 5, as the considered alternative hospitalisation could be "cost-effective", if NHS cost-effectiveness threshold would be applicable for hospital services. Therefore, ICER for rotavirus vaccination vs. no vaccination was lower than the baseline estimate when opportunity costs were considered in the scenario 5. For the other scenarios, negative net health benefit was expected, therefore ICER considering this opportunity cost was higher than the baseline estimate.
- Approach 10. The rotavirus vaccination could be associated with additional gross health benefit and gross health expenditure, as estimated for the approaches 3 and 5 respectively. Similarly to the approaches 6-9, ICER for rotavirus vaccination vs. no vaccination was lower than the baseline estimate only for the scenario 5.

As previously shown for the public health impact, all estimated opportunity costs were lower for about 30% when the 3-months timeframe for the peak incidence season was considered, however the ICERs demonstrated the same trends as shown for 6-months timeframe.

Overall, for the approaches 3 and 4, which were considered as the most relevant for this study, the ICER considering opportunity costs was lower than the baseline for all the explored

scenarios. The lowest estimate (£11,552/ QALY gained) was obtained for the scenario 5 which considered the most severe condition being a cause of the hospitalisation displacing RVGE.

Table S6. Results – Opportunity costs expressed in health benefits and associated costs (peak incidence season for 6-months timeframe).

#	Approach	Scenario 1: RTT admitted	Scenario 2: URTI	Scenario 3: URTI mild	Scenario 4: URTI severe	Scenario 5: Non-gastroenteritis cases, with chronic conditions	Scenario 6: Non-gastroenteritis cases, without chronic conditions
**Parameters for non-gastroenteritis cases:**<br>• Cost of hospitalisation (C_{other})<br>• LOS (LOS_{other})<br>• QALY gain associated with hospitalisation (H_{other})		• £ 1,364<br>• 1.2<br>• 0.0047	• £ 409<br>• 1.0<br>• 0.0014	• £ 307<br>• 0.5<br>• 0.0010	• £ 511<br>• 2.1<br>• 0.0017	• £ 1,270<br>• 5.4<br>• 0.239	• £ 1,270<br>• 3.3<br>• 0.002
Opportunity costs (OCs)							
3	Gross health benefit forgone for the second-best patient-equivalents (PE)	132	45	65	27	1,461	20
4	Health benefit forgone for the best alternative use (TE)	614	614	614	614	614	614
5	Gross expenditure saved on the second-best* patient-equivalents (PE)	£38,134,624	£13,501,472	£19,458,515	£8,047,903	£7,760,420	£12,698,869
6	Net monetary benefit forgone for the second-best patient-equivalents (PE)	-£35,503,605	-£12,597,813	-£18,156,149	-£7,509,253	£21,456,128	-£12,298,795
7	Expenditure incurred + net monetary benefit forgone (PE)	-£23,233,290	-£327,498	-£5,885,834	£4,761,062	£33,726,443	-£28,480
8	Gross monetary benefit forgone for the second-best patient-equivalents (PE)	£2,631,019	£903,659	£1,302,366	£538,649	£29,216,549	£400,074
9	Net health benefit forgone for the second-best patient-equivalents (PE)	-1775	-630	-908	-375	1073	-615
10	Gross expenditure saved and gross health benefit forgone on the	OC3 and OC5 as above					

#	Approach	Scenario 1: RTT admitted	Scenario 2: URTI	Scenario 3: URTI mild	Scenario 4: URTI severe	Scenario 5: Non-gastroenteritis cases, with chronic conditions	Scenario 6: Non-gastroenteritis cases, without chronic conditions
	second-best patient-equivalents (PE)						
ICER (the baseline value derived from the study by Martin et al. was £ 23,337 / QALY gained)							
3	Gross health benefit forgone for the second-best patient-equivalents (PE)	£21,374	£22,624	£22,322	£22,907	£11,552	£23,016
4	Health benefit forgone for the best alternative use (TE)	£22,336	£22,336	£22,336	£22,336	£22,336	£22,336
5	Gross expenditure saved on the second-best* patient-equivalents (PE)	£41,399	£24,197	£28,357	£20,389	£20,188	£23,637
6	Net monetary benefit forgone for the second-best patient-equivalents (PE)	£48,130	£32,135	£36,016	£28,581	£8,354	£31,926
7	Expenditure incurred + net monetary benefit forgone (PE)	£48,130	£32,135	£36,016	£28,581	£8,354	£31,926
8	Gross monetary benefit forgone for the second-best patient-equivalents (PE)	£30,069	£31,275	£30,997	£31,530	£11,503	£31,627
9	Net health benefit forgone for the second-best patient-equivalents (PE)	£41,664	£41,664	£63,754	£31,631	£13,342	£40,902
10	Gross expenditure saved and gross health benefit forgone on the second-best patient-equivalents (PE)	£91,381	£31,764	£35,320	£28,423	£14,235	£31,762

* ICERs computed within approaches 5 to 10 should be interpreted with caution, as those incorporate the efficiency of the alternative use of the resource.

Table S7. Results – Opportunity costs expressed in health benefits and associated costs (peak incidence season for 3-months timeframe).

#	Approach	Scenario 1: RTT admitted	Scenario 2: URTI	Scenario 3: URTI mild	Scenario 4: URTI severe	Scenario 5: Non-gastroenteritis cases, with chronic conditions	Scenario 6: Non-gastroenteritis cases, without chronic conditions
	**Parameters for non-gastroenteritis cases:**<br>• Cost of hospitalisation (C_{other})<br>• LOS (LOS_{other})<br>• QALY gain associated with hospitalisation (H_{other})	• £ 1,364<br>• 1.2<br>• 0.0047	• £ 409<br>• 1.0<br>• 0.0014	• £ 307<br>• 0.5<br>• 0.0010	• £ 511<br>• 2.1<br>• 0.0017	• £ 1,270<br>• 5.4<br>• 0.239	• £ 1,270<br>• 3.3<br>• 0.002
	Opportunity costs (OCs)						
3	Gross health benefit forgone for the second-best patient-equivalents (PE)	88	30	44	18	977	13
4	Health benefit forgone for the best alternative use (TE)	410	410	410	410	410	410
5	Gross expenditure saved on the second-best* patient-equivalents (PE)	£25,499,295	£9,027,964	£13,011,232	£5,381,352	£5,189,123	£8,491,292
6	Net monetary benefit forgone for the second-best patient-equivalents (PE)	-£23,740,025	-£8,423,719	-£12,140,385	-£5,021,176	£14,346,966	-£8,223,776
7	Expenditure incurred + net monetary benefit forgone (PE)	-£15,535,292	-£218,986	-£3,935,652	£3,183,556	£22,551,698	-£19,043
8	Gross monetary benefit forgone for the second-best patient-equivalents (PE)	£1,759,271	£604,245	£870,847	£360,176	£19,536,089	£267,516
9	Net health benefit forgone for the second-best patient-equivalents (PE)	-1,187	-421	-607	-251	717	-411
10	Gross expenditure saved and gross health benefit forgone on the second-best patient-equivalents (PE)	OC3 and OC5 as above					
	ICER (the baseline value derived from the study by Martin et al. was £ 23,337 / QALY gained)						

#	Approach	Scenario 1: RTT admitted	Scenario 2: URTI	Scenario 3: URTI mild	Scenario 4: URTI severe	Scenario 5: Non-gastroenteritis cases, with chronic conditions	Scenario 6: Non-gastroenteritis cases, without chronic conditions
3	Gross health benefit forgone for the second-best patient-equivalents (PE)	£21,987	£22,855	£22,649	£23,048	£13,874	£23,121
4	Health benefit forgone for the best alternative use (TE)	£22,594	£22,594	£22,594	£22,594	£22,594	£22,594
5	Gross expenditure saved on the second-best* patient-equivalents (PE)	£35,415	£23,912	£26,694	£21,366	£21,232	£23,538
6	Net monetary benefit forgone for the second-best patient-equivalents (PE)	£39,916	£29,220	£31,815	£26,844	£13,319	£29,080
7	Expenditure incurred + net monetary benefit forgone (PE)	£39,916	£29,220	£31,815	£26,844	£13,319	£29,080
8	Gross monetary benefit forgone for the second-best patient-equivalents (PE)	£27,838	£28,645	£28,459	£28,815	£15,424	£28,880
9	Net health benefit forgone for the second-best patient-equivalents (PE)	£136,405	£33,062	£40,509	£28,299	£15,549	£32,738
10	Gross expenditure saved and gross health benefit forgone on the second-best patient-equivalents (PE)	£38,763	£29,029	£31,467	£26,759	£16,028	£28,996

* ICERs computed within approaches 5 to 10 should be interpreted with caution, as those incorporate the efficiency of the alternative use of the resource.

4. Discussion

The analysis presents an illustrative case study capturing the value of vaccination for health system strengthening in CEA. Introducing rotavirus vaccination could lead to additional health benefits with the resources becoming available due to fewer RVGE hospitalisations - this was illustrated within a range of scenario analyses and approaches tested. However, considerable uncertainty remains for the future application of the explored methodology. Key areas to investigate further are summarised below:

- This study considered that maximizing health benefit is a priority for decision-makers. Therefore, approaches 1-4 were proposed as the most relevant for the estimation of the

opportunity costs associated with RVGE hospitalisations in the absence of rotavirus vaccination. Other approaches (approaches 5-10) should be interpreted with caution, since they reflect not only benefits of vaccination, but also the efficiency of the alternative use of resources, applying cost and health outcomes for the alternative hospitalisation, and/or WTP threshold. However, it is not clear whether such net benefit estimates should be derived using the cost-effectiveness threshold of £20,000-30,000/QALY gained used by NICE for HTA. Indeed, this WTP threshold may not be suitable for valuing hospital interventions or admissions, especially when they occur in emergency and/or paediatric services. Furthermore, it is likely that in practice the decision for providing health services or admitting a patient will be based on the clinical need rather than cost-effectiveness of these services [18-20]. The implications for the cost-effectiveness of alternative use of resources, as well as differences between health systems and HTA practices across different countries, should be taken into account for the future studies.

- There is considerable uncertainty regarding the nature of other diseases, for which beds becoming available will be used. The most suitable methodology identified to date [3] considered the decision space with only two mutually exclusive options (e.g., gastroenteritis and non-gastroenteritis hospitalisation). Therefore, all alternative hospitalisations could plausibly represent the "next-best" alternative use, which is in line with the definition of opportunity costs. However, dedicated evidence generation studies are needed to inform such analyses and provide relevant data for the target population and intervention of interest.
- Three scenario analyses (2-4) were designed to explore the opportunity costs of the forgone RVGE hospitalisation being replaced by URTI of different severity, which was taken as a proxy for alternative cause of hospitalisation in line with the evidence on high incidence and largely overlapping peak seasons for RVGE and URTI. These scenarios also adopted an assumption that delayed hospitalisation could result in more severe disease and higher QALY losses. The health benefit associated with timely admission was not expected to be high for the paediatric population, however it could be more important for older adults with severe diseases, when longer waiting time and leaving the emergency room without being seen could result in delayed diagnosis and significant worsening of the condition.
- Further studies estimating opportunity costs of the health system resource use could potentially capture the broader range of resources (e.g., GP visits, nurse time, emergency visits, etc.) and additional benefits stemming from the decreased pressure on health facilities, e.g., considering non-fixed hospital resources or outpatient services, as in the study by Sandmann et al. [3], where 15% of variable healthcare cost savings were taken into account. Also, health benefits and potential cost savings of timely vs. delayed hospitalisation, emergency admission or physician visit, as well as cost of the lost productivity and leisure time could be taken into account.

Supplementary S4. Case study: MenB vaccination with distributional cost-effectiveness analysis

5. Background

This analysis aimed to extend the previous cost-effectiveness analysis for 4CMenB (GSK) vaccination in England [29] and consider the additional value of vaccination for health equity. Health equity was considered to be a key priority value of vaccination concept to expand current value considerations applied in HTA of vaccines.

Infant vaccination in England against invasive meningococcal disease (IMD) serogroup B (MenB) was considered for this case study, due to availability of equity-stratified data for disease and at the population-level and observational evidence showing socioeconomic status is a risk factor for meningococcal disease [30]. Equity benefits of vaccination can be captured with distributional cost-effectiveness analysis (DCEA) [31-33], where the population is stratified by health equity strata. For this analysis the Index of Multiple Deprivation approximative of socioeconomic status was considered and the population was stratified into five subgroups using the Index of Multiple Deprivation quintiles (IMDQ). The IMDQ is an official measure of deprivation in England based on income, employment, education and health, among other indicators [34].

6. Methods

6.1. The original model overview

The objective of the original health economic analysis was to assess the cost-effectiveness of introducing 4CMenB for active immunisation against MenB into the National Immunisation Programme (NIP) in England, with a 2+1 dosing schedule given at 2, 4 and 12 months in infants, compared with no MenB vaccination [35]. However, the developed model once populated with appropriate inputs (dosing scheme, coverage, vaccine effectiveness parameters) enables comparison of alternative vaccination strategies for 4CMenB and/or MenACWY vaccines with a flexible dosing scheme [36].

The main settings of the original model are summarised in the Table S8.

Table S8. The original model settings.

Model settings	Description
**Population**	General population 0 to 99 years of age
**Target population**	Infants at 2, 4 and 12 months of age<br><br>In the model, dosing schedules are flexible and can be considered in infants, aged 0 to 12 months old, toddlers and adolescents (with a flexible definition of age ranges)
**Perspective**	Payer and societal
**Time horizon**	100 years lifetime horizon, with associated direct and indirect costs, and QALY losses due to deaths and sequelae
**Interventions considered**	• 2+1 4CMenB (Bexsero) infant NIP with dosing schedule at 2, 4 and 12 months<br>• No vaccination

**Country perspective**	England
**Analytic technique**	A cost-utility analysis

The underlying epidemiological process for the transmission of meningococcal infection has been simulated using a classical susceptible-infected-susceptible (SIS) epidemic model.

The set of original model compartments included susceptible and infected, i.e., carriers of *Neisseria meningitidis* divided by serogroup into three categories: (1) serogroup B only, (2) serogroups A, C, W and Y, and (3) other pathogenic and non-pathogenic serogroups. Each of these compartments is further subdivided into vaccinated and not vaccinated, and the vaccinated subcompartments into vaccinated as infants, as toddlers and as adolescents.

Additionally, there were three observational compartments for tracking incident cases, separately for serogroups MenB, MenACWY and other meningococcal strains (MenOther). Thus, the DTM 'observed' the occurrence of invasive meningococcal disease cases after acquisition of carriage. The model captured direct vaccine protection, i.e., prevention of invasive meningococcal disease in vaccinees, and indirect protection due to the decreased transmission of meningococcal carriage to non-vaccinated individuals (herd immunity).

The presented study did not assume any impact of 4CMenB on carriage acquisition of MenB, MenACWY and MenOther strains [37].

The DTM structure flow chart is presented in Figure S1 below.

To evaluate the cost-effectiveness of different vaccination strategies, relevant costs and health outcomes of each vaccination strategy were assessed, within a decision tree cost-effectiveness model (CEM). For each incident case of invasive meningococcal disease simulated in the DTM, a decision tree model was applied with branches for the probability of various outcomes with assigned relevant costs and health outcomes of each vaccination strategy. Two main categories of costs were considered related to cases of invasive meningococcal disease and vaccination, respectively, while the benefits of vaccination were expressed in terms of LY/QALY gains related to disease cases prevented.

After developing invasive meningococcal disease, all patients were assumed to experience the acute phase of disease. During or close to the end of the acute phase, patients either survive or die. Survivors may be either free from long-term sequelae or develop one or more long-term sequelae, also the status of survivor was reflected in the model by the adjusted life expectancy. The decision tree represented all possible combinations from acute disease, survival and occurrence of 16 types of long-term sequelae that may occur as a consequence of invasive meningococcal disease, and the corresponding probability of each of these. The resulting probabilities were then used for calculating the expected number of health outcomes and economic outcomes for a case of invasive meningococcal disease.

The structure of the decision tree CEM is presented in Figure S2.

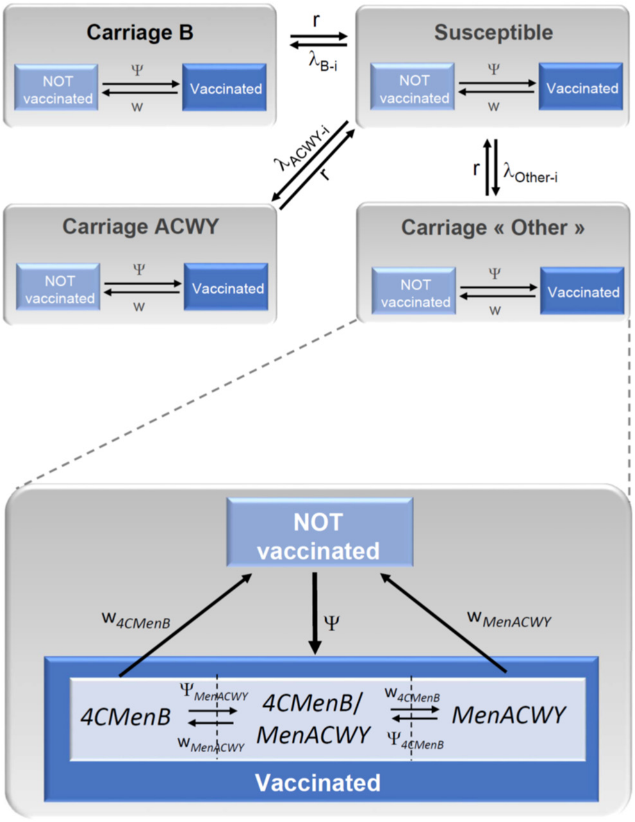

Figure S1. Structure of the DTM.

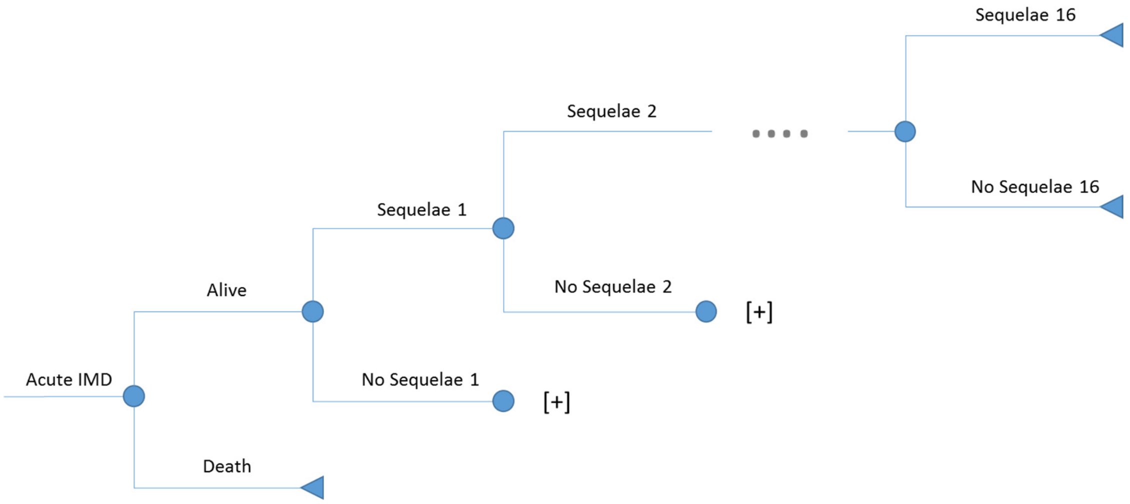

Figure S2. Structure of the CEM.

Ultimately, the DTM generated epidemiological outputs that were further used in the decision tree CEM for the economic analysis. The overview of the model is shown in Figure S3.

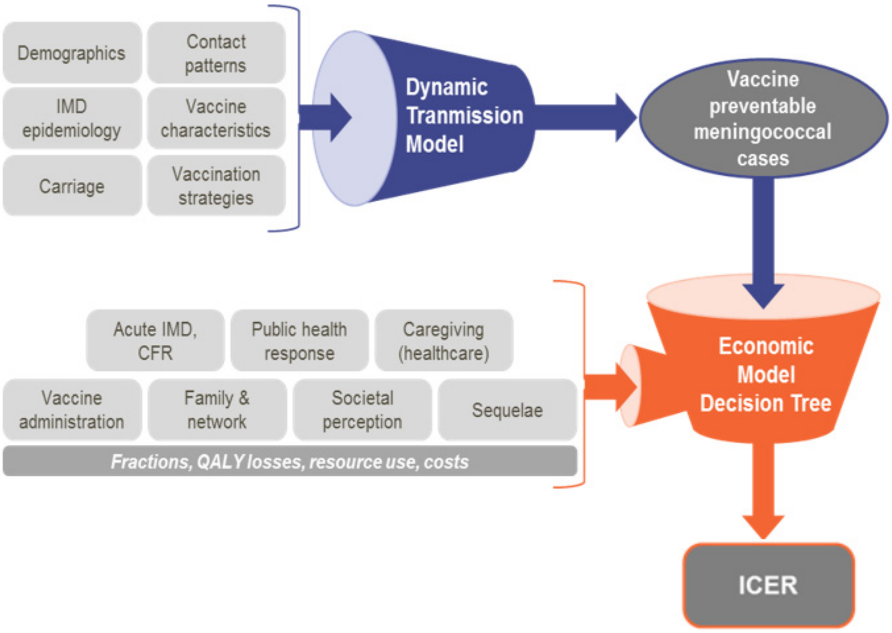

Figure S3. Overview of the model structure.

6.2. Model adaptation considering health equity

The additional value of vaccination for the equity can be captured through the framework of distributional cost-effectiveness analysis (DCEA) [31-33] where generally speaking the overall or general population is stratified by health equity strata. In the present case study, the general population of England is stratified into five subgroups, depending on the socioeconomic status, represented by the Index of Multiple Deprivation quintiles (IMDQ) [34].

The model adaptation did not consider modification of the DTM due to significant complexity associated with equity-stratified simulation of occurrence of cases in the no vaccination scenario and impact of vaccination, while the limitations of this approach were acceptable for this case study. E.g., this methodology did not capture the impact of vaccination on MenB transmission between strata and the related herd effect, however there is currently no clear evidence on this type of protection for MenB vaccine. Thus, stratification with regards to equity was implemented for inputs and outputs of the CEM. The DTM was run only once to simulate outcomes (incidence of invasive meningococcal disease) for the general population. This step assumed that DTM inputs were not changed. Then, the obtained number of cases (per serogroup and age) was retrospectively adjusted to account for differences in invasive meningococcal disease incidence across considered socioeconomic strata.

A step-wise approach was applied to consider the impact of 4CMenB vaccination on equity. First, cost and effects were calculated to provide distribution of outcomes across the population which is similar to public health impact analysis in traditional CEA of vaccines. Second, the net health equity impact was evaluated. Third, the equity impact was incorporated into the CEA framework using distributed cost-effectiveness modelling. In general, computation of model outcomes was done for each stratum separately and the cost-effectiveness was estimated for the total population.

Stratification

Rationale

The model adaptation considered stratification of the target population according to the socioeconomic status, represented by IMDQ. The index of multiple deprivation is an official measure of deprivation in England at local authority district level based on income, employment, education and health among other indicators [34].

Notably, this index does not reflect the characteristics of an individual, however it captures the social deprivation at the level of small areas, incorporating the spatial differences within the general population. Considering the contagious nature of the modelled disease, and the relatively small size of the geographic areas included (1,500 inhabitants on average), this measure was considered as an appropriate proxy for the stratification of the individuals in the target population. The UK national framework considers 10 deprivation categories, however this analysis considered less granular classification with 5 categories to avoid introducing the excessive complexity into the analysis. This approach is in line with the previous studies applying the DCEA framework [38,39] and recommendations of the advisory board with external experts preceding the development of the current analysis. This study focuses on socioeconomic status reflected by the IMDQ, where the first quintile (IMDQ1) is the most deprived and the fifth (IMDQ5) is the least deprived. The adapted model allows to stratify population by other equity strata (i.e., social or economic determinants), with up to five categories and thus could be adapted straightforwardly to other country contexts.

Methodological approach

The DTM was run only once to produce the number of invasive meningococcal disease cases (stratified by serogroup to MenB, MenACWY and MenOther; age and a simulated time step) for the general population, which was further split into five strata, for the vaccinated and unvaccinated individuals separately. Stratification was applied to the number of cases using a specific coefficient representing an estimated case proportion for each stratum, assuming that the total number of cases (as provided by the original model) incorporates all social inequalities, thus only a distribution of cases in different strata has to be derived.

The estimated case proportion was calculated for both the vaccination and no-vaccination scenario using the following stratum-specific parameters:

- Proportion of each stratum in the total population,
- Meningococcal carriage prevalence,
- Vaccine effective coverage (EC), considering the vaccination coverage, protection against invasive meningococcal disease and waning of protection.

The calculation was performed using the formula below:

$$Estimated\ case\ proportion_{stratum\ j} = \frac{Proportion_{stratum\ j} * Carriage_{stratum\ j} * \left(1 - Average\ EC_{stratum\ j}\right)}{\sum_{i=1}^{5}(Proportion_{stratum\ i} * Carriage_{stratum\ i} * (1 - Average\ EC_{stratum\ i}))},$$

where $Average\ EC_{stratum\ j}$ *is an average effective coverage over time of* j-th stratum.

The estimated case proportion can be also considered as an average proportion of individuals in the stratum being not protected.

For the vaccinated arm, an estimated case proportion of MenB cases considered direct protection provided by 4CMenB, an estimated case proportion of MenACWY cases considered direct cross-protection, and no protection was assumed for MenOther cases.

For the unvaccinated arm effective coverage was assumed equal to 0%, in that case the formula for the estimated case proportion included only stratum proportion in the population and carriage prevalence. The same estimated case proportion was used for MenOther cases.

$Average\ EC_{stratum}$ was derived aside of the DTM in a simplified way assuming that each next dose of the vaccine supersedes or is equal to the effectiveness of the previous one. Thus, the proportion of protected individuals at the time of the dose administration is equal to the dose effectiveness times the dose coverage by stratum, then it drops according to the waning function until the next dose is administrated, and the whole process is repeated. To calculate the average effectiveness, the area under the plotted function ('area under the curve' (AUC)) of the effective coverage was computed by integrating the function on the intervals defined by the time points of two subsequent vaccine doses. It was assumed that the effective coverage before the first dose is equal to 0%. The last interval is defined from the last dose timepoint to the T_{MAX} equal to the considered timeframe. Finally, to obtain the average effective coverage, these integrals were added up and divided by the length of the time interval $[0;\ T_{MAX}]$.

Table S9 summarises all calculation steps.

Table S9. Average vaccine effectiveness per stratum over time – calculations steps.

Time point	Dose	Effective coverage	Protection function	Effective coverage at time t	EC over time (area under the curve)
**T1**	D1	EC1	P1	EC(t) = EC1*P1(t-T1)	$\int_{T1}^{T2} EC(t)dt$
**T2**	D2	EC2	P2	EC(t)=EC2*P2(t-T2)	$\int_{T2}^{T3} EC(t)dt$
**T3**	D3	EC3	P3	EC(t)=EC3*P3(t-T3)	$\int_{T3}^{T4} EC(t)dt$
**T4**	D4	EC4	P4	EC(t)=EC4*P4(t-T4)	$\int_{T4}^{T_{MAX}} EC(t)dt$
**Total EC**	$\int_0^{T_{MAX}} EC(t)dt = \int_{T_1}^{T_2} EC(t)dt + \int_{T_2}^{T_3} EC(t)dt + \int_{T_3}^{T_4} EC(t)dt + \int_{T_4}^{T_{MAX}} EC(t)dt$				
**Average EC**	$\frac{1}{T_{MAX}} * \int_0^{T_{MAX}} EC(t)dt$				

EC, Effective coverage, computed as $Coverage\ \times Effectiveness$, for each stratum.
The original model allows to include up to 4 doses, the reference case considers 3 doses.

For the reference case analysis, an average effective coverage was computed by stratum for the timeframe of 5 years, as the majority of cases are observed in children < 5 years. Additionally, with the last dose of vaccination at 12 months of age and persistence duration of 38 months, no significant vaccine effect is expected beyond this timeframe.

Two alternative approaches could be applied for the estimation of EC:

- AUC and average EC could be computed for each yearly cycle using the formulas provided in Table S9, with the estimated case proportion applied to predicted cases by year.
- AUC and average EC for each year separately could be approximated using a simplified methodology, with EC for a certain period computed as the product of coverage and mid-period efficacy (derived from the waning function). This approach was implemented for monthly intervals with further averaging over a year (as a proxy for yearly AUC).

For this analysis, the estimated case proportion considered only direct and cross-protection against invasive meningococcal disease, for MenB and MenACWY respectively, as the carriage effect (and potential herd immunity) was not included in the reference case of the original 4CMenB infant NIP analysis [29]. It could be considered as a plausible approximation of the reality, as no impact on transmission or herd effect was so far observed for MenB vaccination [37,40], as well as for infants the herd effect reducing transmission in the total population would likely be of lower magnitude than in comparison to an adolescent vaccination programme targeting the age-group with the higher prevalence of meningococcal carriage. The applied approach assumes the incidence varies across strata due to different meningococcal carriage prevalence and different vaccination coverage. There is no evidence available to our knowledge that the case carrier ratio, i.e., the risk of getting meningococcal disease given carriage, is impacted by socioeconomic status, thus the assumption was made that the case-carrier ratio is the same across different IMDQs for each age. This also follows the approach considered in the original model where the case-carrier ratio is assumed to be constant over time for each age group. However, this assumption could be withdrawn to consider stratum-specific case-carrier ratios for the calculations, in case these data would become available. The formula would then include case-carrier ratios as an additional multiplier for the numerator and denominator, similarly to the carriage prevalence. Similarly, vaccine efficacy and waning were not assumed to be impacted by socioeconomic status, therefore these parameters were also set equal for all strata.

Importantly, all estimated case proportions should be considered as proxies, developed for the preliminary assessment prior to the comprehensive DCEA, in comparison to equity-stratified estimates on the number of invasive meningococcal disease cases derived from a DTM adapted to capture equity. Particularly, stratum-specific epidemiologic and vaccination-related inputs, contacts between individuals from the different strata, movement of individuals between strata over time, equity-stratified herd effect and protection against carriage transmission could be appropriately captured only within a future model adaptation based on an equity-stratified DTM.

Health equity analysis

Rationale

DCEA could be used to inform decisions related to two main objectives: (1) maximizing total health benefit obtained with the limited resources, and (2) reducing health inequality [31]. The optimal pursuit of these objectives could be seen as a maximization of the social welfare. Mathematically, it could be measured as a numerical index, which considers the distribution of benefits in a population, and value judgements about the appropriate trade-offs between reducing inequality and improving total benefit. These value judgements can be represented

by an inequality aversion parameter [31,33]. Further, an inequality aversion parameter could be incorporated into DCEA within a social welfare function (SWF). This analysis applied values of inequality aversion parameters informed by the literature, to assess the distribution of vaccination benefits across strata.

Vaccination may deliver a broad range of benefits to the society and an individual, of those health benefits and financial benefits are the most applicable for the HTA framework. This study focused on the health-related benefits. Explicit modelling of the distribution of financial benefits of vaccination, including the statistical relationship between health outcomes and health expenditure, as well as out-of-pocket payments, financial risk protection and other monetary measures could be considered for the further analyses.

Further, a stepwise approach was applied to account for equity in public health impact and cost-effectiveness analysis building upon the DCEA framework [41]:

- Step 1. Assessment of the distributional impact of vaccination.
- Step 2. Assessment of the equity impact of vaccination.
- Step 3. Accounting for equity in full DCEA.

For the purpose of the health equity analysis, total QALYs were introduced as an additional outcome, in line with the published literature on DCEA, referring to the lifetime health, or health-adjusted life expectancy as the health outcome of interest [31-33,42]. Furthermore, inequality aversion parameters were elicited also for the lifetime health estimate (expected years of life in full health) [43], and it is not clear if aversion to inequality in a society is the same for total health and other health outcomes (e.g., QALY loss), which raises the concern on the applicability of the estimated inequality aversion parameters for outcomes other than total QALYs.

Methodology: Step 1. Distributional impact of vaccination (‘Public Health Impact Analysis’)

The first step in analysing the impact of 4CMenB vaccination on equity is considering the public health impact, i.e., the impact on health outcome distribution by IMDQ (for total QALY, QALY loss, incidence rate, the number of long-term sequelae, the number of cases and the number of deaths related to invasive meningococcal disease) with and without vaccination. This step also mirrors the approach to public health impact and cost-effectiveness analysis of vaccination programmes in general, where in a first step usually the public health impact is studied, estimating most often the number of infectious disease cases prevented by comparison of the vaccination and no vaccination scenario. For the present equity analysis, health outcomes for this step were presented in tabular and graphical format, aiming to compare the expected distribution of benefits of the intervention across the different strata.

Additionally, slope index of inequality (SII) was calculated for each outcome. SII is an absolute measure of inequality and it represents a linear regression coefficient β_1 in the outcome distribution in population described as $Q_j = \beta_0 + \beta_1 r_j + \varepsilon_j$, where r_j is the fractional rank, j is number of each group, β_0 is a constant and ε_j is a random error.

SII was computed using the following formula:

$$SII(\beta_1) = \frac{\frac{1}{J}\sum_{j=1}^{J}(Q_j - \bar{Q})(r_j - \bar{r})}{\frac{1}{J}\sum_{j=1}^{J}(r_j - \bar{r})^2}$$

where J is a number of group, Q_J is an outcome for an individual in a stratum J, $\bar{Q}$ is a mean outcome across individuals in all strata, $\bar{r}$ is a fractional rank.

The fractional rank was calculated as a cumulative mid-point of the group within the distribution from the lowest to the highest value of the outcome. For example, if groups 1, 2 and 3 represent 10%, 15% and 20% of the population, respectively, then the fractional rank for each group is calculated as: 0.1/2=0.05 for a group 1, 0.1+0.15/2=0.175 for a group 2 and 0.1+0.15+0.2/2=0.35 for a group 3.

SII provides a numeric measure describing the distribution of outcomes across individuals in strata, it is estimated on the same scale as the outcome and it is relatively easy to interpret. Thus, higher SII suggests higher inequality, and when the difference between SII in the vaccination scenario vs. no vaccination scenario is below zero, it could be concluded that this vaccination strategy improves equity. However, SII is dependent on the mean level of health in population and the distribution of strata, therefore it should not be compared for different populations and outcomes. Of note, SII was computed for individual-level outcomes and incidence rate since it is not clear if this methodology is applicable for aggregated population-level outcomes.

Despite this study was focused on the health outcomes, the impact of 4CMenB vaccination on the distribution of costs across IMDQ was also explored. Total costs and net monetary benefit (NMB) were presented for payer and societal perspective. NMB per IMDQ and in total population was computed as follows:

$$NMB_{IMDQ} = Health\ benefit_{IMDQ} \times Cost - effectiveness\ threshold \\ - Total\ cost\ of\ an\ intervention \times Proportion\ in\ population_{IMDQ}$$

$$NMB_{Total\ population} \\ = Total\ health\ benefit \times Cost - effectiveness\ threshold \\ - Total\ cost\ of\ an\ intervention$$

Methodology: Step 2. Equity impact of vaccination

In a standard CEA, the health benefit for the society (social welfare) is measured simply as an average health benefit for the total considered population. The more the society gains as total, the greater health benefit is expected. Within the DCEA which values equity, it is considered that the smaller differences between subgroups for the benefits result in the greater equity of a given intervention. One of the approaches to incorporate equity dimension into DCEA is to transform health benefit of individuals by a SWF. Equity based SWF utilizes ethics of prioritarianism, in that case it gives higher priority to individuals with a lower health benefit. Mathematically, SWF is a function that is strictly increasing and strictly concave (with respect to health benefit). The first property reflects the rule that the higher benefit is advantageous for society. The second property means that improving health of individual with lower health benefit generates more value to society than improving health of an individual with higher benefit [31]. Following the approach of DCEA as described in [31], health benefit was represented by total QALYs in this analysis. According to Asaria et al. [33]. "A common feature of such functions (i.e., SWF) is the need to specify the nature of and level (or value) of inequality aversion. The inequality aversion parameters in these functions describe the trade-off between total health and the level of health inequality (i.e., the amount of total health that a decision maker would be willing to sacrifice to achieve a more equal distribution). These inequality aversion parameters are difficult to interpret on a raw scale. A more intuitive scale can be provided by combining a specific value of the

parameter with a specific health distribution to derive the equally distributed equivalent (EDE) level of health. The difference between the mean level of health in that distribution and the EDE level of health then represents the average amount of health per person that one would be willing to sacrifice to achieve full equality in health, given that specific value of inequality aversion."

DCEA allows to combine health benefits in an equity impact estimate, represented by the equally distributed equivalent health (EDEH). For this analysis two EDEH measures were used:

- Atkinson EDEH which reflects the relative inequality (scale-invariant) in health benefit,
- Kolm-Pollak EDEH which reflects the absolute inequality (translation invariant) in health benefit [31,33].

EDEH indices for strategies with and without vaccination were computed using the following formulas:

$$EDEH_{Atkinson} = \left[\frac{1}{N}\sum_i (H_i)^{1-\varepsilon} \cdot n_i\right]^{1/(1-\varepsilon)}$$

$$EDEH_{Kolm-Pollak} = -\left(\frac{1}{\alpha}\right) log\left(\frac{1}{N}\sum_i e^{-\alpha H_i} \cdot n_i\right)$$

where ε and α are parameters of a decision-maker aversion to inequality (of relative and absolute health benefit inequality respectively), N is the size of the total population, H_i is the health outcome for the stratum i, and n_i is the number of individuals in the stratum.

Finally, index of inequality was calculated for each assessed strategy, depending on the measure.

Index of inequality based on Atkinson's EDEH was calculated as:

$$A(\varepsilon) = 1 - \frac{EDEH(\varepsilon)}{\mu}$$

where μ is the mean health benefit in total population per strategy (e.g., mean total QALY for not vaccinated). Result equal to 0 represents no inequality, while 1 represents full inequality.

Index of inequality based on Kolm-Pollak's EDEH was calculated as:

$$K(\alpha) = \mu - EDEH(\alpha).$$

Net equity impact for assessed vaccination strategy was defined as the difference between the index of inequality of no vaccination strategy and the index of inequality of a given vaccination strategy.

Further, to facilitate the interpretation of DCEA outcomes, this analysis used net health benefit as an integral measure of cost-effectiveness (also representing "efficiency impact"), thus the decision-making process could operate within a two-dimensional space defined by equity and efficiency. Net health benefit per IMDQ and in total population was computed as follows:

$$NHB_{IMDQ} = Health\ benefit_{IMDQ} - \frac{Total\ cost\ of\ an\ intervention * Proportion\ in\ population_{IMDQ}}{Cost - effectiveness\ threshold}$$

$$NHB_{Total\ population} = Total\ health\ benefit - \frac{Total\ cost\ of\ an\ intervention}{Cost - effectiveness\ threshold}$$

Thus, the net health benefit considers the health opportunity cost of the intervention considering the cost-effectiveness threshold of £20,000 per quality-adjusted life year (QALY) [33], i.e. the same cost-effectiveness threshold as for the underlying cost-effectiveness analysis of Beck et al. [29]. Based on expert insights from latest unpublished research and previous DCEA [33], health opportunity costs were apportioned equally across IMDQs, i.e., the same threshold and cost for each individual within each IMDQ was applied. Cost within this context also refer to the net cost of the intervention considering both actual costs of the intervention as well as cost offsets occurring through prevention of disease. While for the direct costs (i.e., payer perspective) an assumption on the equal distribution of the health opportunity cost across IMDQ seems realistic, it is not clear how the cost savings stemming from indirect costs could be distributed. For the purpose of this study, the same assumption on the equal distribution of health opportunity costs was taken for both perspectives, however NHB computed for the societal perspective should be interpreted with caution.

The obtained equity and efficiency measures for the intervention were plotted on the 2-by-2 plane, with four possible combinations of the CEA conclusions and potential equity impact [31] (Figure S4).

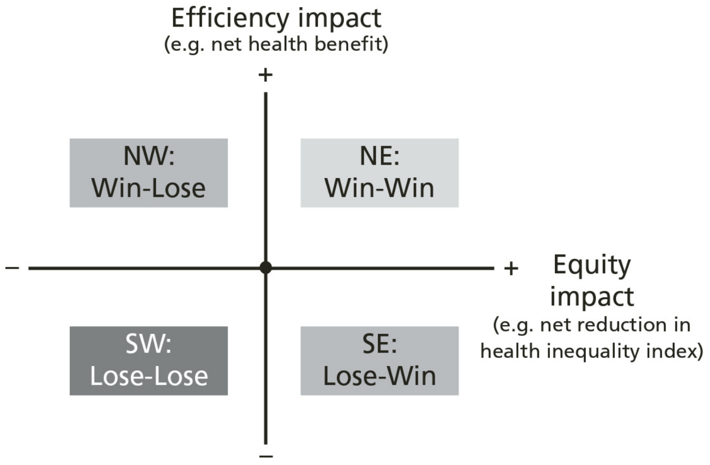

Figure S4. Equity-efficiency impact plane [31].

Methodology: Step 3. Accounting for equity in full DCEA

The possibility to account for equity in the full DCEA was explored, using two alternative approaches: (1) level-dependent equity weighting for QALYs; (2) weighting of the threshold.

Level-dependent equity weights for QALYs was implemented in line with the previous recommendations issued by NICE for modifiers [44], where weighting for health outcomes was considered, however the methodology for estimation of weights was not provided by NICE. For this pilot study, the methodology proposed by Professor Richard Cookson [31] and detailed during the expert consultation meeting was applied as described below.

Two different formulations of the QALY weighting were implemented according to the SWF considered, i.e., using the Atkinson's or Kolm-Pollak's inequality aversion parameter.

The level-dependent health maximising SWF can be written as follows:

$$W = \sum_{i=1}^{N} g(H_i)$$

where W is social welfare, N is the total population size, H_i the health of an individual i and $g(h_i)$ is a strictly increasing and concave transformation function of health.

This transformation function was computed for each IMDQ-defined subgroup i for Atkinson's and Kolm-Pollak's SWF as follows:

$$g(h_i) = \frac{{h_i}^{1-\varepsilon}}{1-\varepsilon}$$

where $\varepsilon > 0$, is the Atkinson's inequality aversion parameter, and

$$g(h_i) = -e^{-\alpha h_i}$$

where α is the Kolm-Pollak's inequality aversion parameter.

Next, the following function was derived for each IMD-defined subgroup i, for Atkinson's and Kolm-Pollak's respectively:

$$g'(h_i) = {h_i}^{-\varepsilon}$$

$$g'(h_i) = g(h_i) \times -\alpha$$

These functions were then used for the calculation of the indirect equity weights ω_i as presented below:

$$\omega_i = \frac{g'(h_i)}{g'(h_\theta)}$$

where θ is the best-off group (i.e., the least deprived socioeconomics group).

Indirect equity weights for no vaccination arm were used for QALY weighting in both arms, vaccinated and not vaccinated. IMDQ5 is the best-off group in the context of this analysis, therefore it is taken as a reference for these computations. Thus, QALY weights for more deprived IMDQ were always higher than one, and the magnitude of weight for a particular IMDQ increased with the higher difference in health between this IMDQ and IMDQ5. Finally, the weighted QALYs for a particular IMDQ reflects the level of health inequality comparing to IMDQ5, and the aversion to inequality in society.

The estimated weights for health outcomes were used to compute the equity-weighted QALYs and respective ICER, further assessed within a standard CEA framework.

Weighting of the willingness to pay (WTP) threshold was applied as an alternative approach to health outcome weighting (described above). Similar methodology is often used by decision-makers to prioritize patients with specific characteristics. Thus, while the standard threshold of £20,000-30,000 per QALY is used for the decision-making, higher thresholds are considered reasonable for "end-of-life" treatments and drugs for very rare diseases, reaching £50,000 and £100,000-300,000 respectively [45,46]. Considering the starting point of £20,000 per QALY, the weights applied to the threshold are 2.5 for "end-of-life" treatments, and 5-15 for interventions targeting very rare diseases. This range of weights

for the threshold (2.5 to 15) will be explored in the planned analysis, with ICER computed using the standard methodology.

Methodology: Introduction of total QALYs as an outcome

The original model estimated the total QALY loss related to the cases of invasive meningococcal disease for the whole modelled population. The latter was comprised of 100 cohorts of individuals at the age of 0 to 99 years at the model start, and 99 birth cohorts, entering the model in each year of the simulation. The model already estimated the discounted quality adjusted life-expectancy according to the age at model entry, using age-dependent utility, age-dependent life-expectancy and discount rate. Introduction of total QALYs as a model outcome followed the same approach, in order to keep consistency between estimated QALY loss and total QALYs.

First, total baseline QALYs accumulated over the lifetime of the modelled population without invasive meningococcal disease had to be estimated in order to derive the average total QALYs per individual for each vaccination strategy. Life expectancy for the individuals without invasive meningococcal disease ("Control" population), was sourced from the original model, and adjusted to general population (or stratum-specific) life expectancy reported for UK.

At model start, the total QALYs accumulated for the starting cohorts was estimated by multiplying the population distribution by the discounted quality adjusted life-expectancy by age. Then, for each birth cohort entering the model in the following years, the total discounted quality adjusted life-expectancy was estimated and expressed in value of the year of birth of the cohort. Then, the discounting over the time horizon of the simulation was applied to express the total accumulated QALYs of these new-born cohorts in current value. Summing the QALYs accumulated by all cohorts (starting population and birth cohorts) provided the total discounted QALYs accumulated over the lifetime of the simulated population without considering the impact of invasive meningococcal disease.

Then for each strategy, the QALY loss related to invasive meningococcal disease (estimated by the original model) was subtracted from the value estimated for population without the disease, to compute the total discounted QALYs accumulated over the lifetime of the simulated population. These values were divided by the total size of the simulated population (size of the starting population + size of each birth cohort) to estimate the average total QALYs by individual which was used for the estimation of equity measures.

The obtained estimate could be interpreted as total QALY per lifetime of an average individual of the simulated population, which includes individuals of different age. Of note, this estimate did not reflect population QALYs from birth to death, applied in published equity analyses [31,33], although its use still can be considered acceptable, as a large proportion of the model population was followed from birth. Furthermore, this population-level approach is routinely used for the CEA of vaccination programs, including previous study on 4CMenB vaccine [29], thus it was applied for this adaptation to ensure comparability of analyses.

Importantly, this model adaptation considered discounting for the health and cost outcomes for the reference case, in line with the standard CEA methodology and previous 4CMenB analysis [29,47], while discounting is not used routinely in analyses on health equity [31,33].

The estimates of the total baseline QALYs accumulated over the lifetime of the modelled strata and total population without invasive meningococcal disease, with and without discounting at annual rate of 1.5% are presented in Table S10.

Table S10. Total baseline QALYs (without invasive meningococcal disease) by IMDQ.

	IMDQ1	IMDQ2	IMDQ3	IMDQ4	IMDQ5	Total population
**Per stratum/population**						
**Not discounted**	1,848,516,446	1,687,618,338	1,478,514,605	1,371,591,519	1,340,450,973	7,726,691,881
**Discounted**	771,283,319	695,159,412	607,101,129	561,020,123	543,521,469	3,178,085,451
**Per individual**						
**Not discounted**	54.602	58.821	60.199	61.077	63.069	59.065*
**Discounted**	22.783	24.229	24.719	24.982	25.573	24.294*

* Weighted average, given proportions of population by IMDQ

6.3. Model outcomes

The original health economic model was designed to provide several health and cost outcomes for each intervention:

- Health outcomes:
  - Number of invasive meningococcal disease cases prevented through vaccination, by serogroup, age and time horizon,
  - Number of deaths related to invasive meningococcal disease prevented through vaccination, by serogroup, age and time horizon,
  - Number of long-term sequelae prevented through vaccination, by serogroup, age, type of sequelae (physical, neurological and psychological/behavioural) and time horizon, and also including,
    - Number of cases experiencing at least one long-term sequela,
    - Number of cases experiencing at least one physical long-term sequela,
    - Number of cases experiencing at least one neurological long-term sequela,
    - Number of cases experiencing at least one psychological/behavioural sequela,
  - Number of cases experiencing severe long-term sequelae which require long-term care,
  - Number of life-years lost due to deaths prevented through vaccination stratified by serogroup approximated by number of QALYs lost due to death,
  - Number of QALYs lost due to invasive meningococcal disease cases stratified by serogroup (total, acute care, long-term sequelae, long-term caregiving),
- Cost outcomes:
  - Direct healthcare costs,
    - Cost of cases in the acute phase (i.e. treatment costs and public health response costs),
    - Long-term medical costs (sequelae),
    - Long-term caregiving and annual cost,
  - Vaccination costs, administration and adverse event costs,
  - Special educational needs costs,

- Public health management and outbreak cost,
  - Indirect costs (productivity loss and litigation costs).
- Cost-effectiveness outcomes:
  - Incremental costs,
  - Incremental QALYs,
  - ICERs: per LY gained, QALY gained, case averted and death averted.

In the equity adapted model the same outcomes were available for both no vaccination and vaccination arm for each stratum and strategy, but the analysis was focused on several main outcomes:

- Health outcomes (per stratum and strategy):
  - Number of invasive meningococcal disease cases caused by MenB,
  - MenB incidence rate,
  - Number of deaths related to invasive meningococcal disease caused by all serotypes,
  - Number of long-term sequelae following invasive meningococcal disease caused by all serotypes,
  - Number of QALYs lost due to invasive meningococcal disease cases caused by all serotypes,
  - Number of total QALYs accumulated over the lifetime in a modelled population within a 100-year time horizon (outcome introduced during the adaptation, to compute equity impact measures).
- Cost outcomes (per stratum):
  - Total costs, for payer and societal perspectives.

Additional outcomes were provided for the adaptation to conduct the DCEA:

- SII,
- EDEH per strategy,
- Index of inequality,
- Net equity impact,
- Incremental equity benefit (difference between NHB computed for equity-weighted vs. unweighted outcomes),
- Incremental QALY (equity-weighted),
- ICER per QALY (equity-weighted),
- Net health benefit,
- Net monetary benefit.

6.4. Model runtime

The runtime of the original model was around 2 min, including calibration, DTM simulations, and CEM update (the latest was the most time-consuming calculation (~1 min), partially coded in VBA, which was required to estimate costs and utilities of sequelae).

The model adaptation did not consider a modification of the DTM, applying stratification for inputs, calculations and outputs of the CEM only. After inclusion of 5 strata, the runtime for reference case analysis increased to ~5 min, as 5 updates of the CEM were needed for all strata, and 1 more update to restore the total population state. More importantly, the runtime for DSA and PSA increased significantly, with ~15 min per parameter for DSA, and ~6 min per parameter for PSA (of note, varying some parameters required also to rerun calibration and DTM). Several optimization techniques were used to shorten the runtime, however significant improvement in efficiency could be easier to achieve, if the equity analysis would be planned at the early stages of the model development.

6.5. Reference case inputs

Clinical and population inputs

<u>DTM output stratification</u>

Population proportions

Proportion of individuals in total population by the index of multiple deprivation quintiles (IMDQ) was used for the stratification. Inputs were based on the Office for National Statistics data for 2015 [48], as provided in Table S11. However, the published data indicate that this proportion varies for different age groups, which should be taken into account for the further model adaptation. Considering the majority of the impact of vaccination to be in infants and young children <5 years of age (y.o.a.), in line with the timeframe chosen for the estimation of the average effective coverage, the estimates for persons aged <5 y.o.a. was used for the reference case.

Table S11. Proportion of UK population by IMDQ.

	Number of individuals, <5 y.o.a.	Distribution of individuals, <5 y.o.a. (ref. case)	Number of individuals, <1 y.o.a.	Distribution of individuals, <1 y.o.a.	Number of individuals, all ages, total population	Distribution of individuals, all ages, total population
**IMDQ1**	888,869	25.9%	174,535	26.3%	11,082,579	20.2%
**IMDQ2**	753,303	21.9%	149,352	22.5%	11,247,869	20.5%
**IMDQ3**	644,855	18.8%	126,225	19.0%	11,001,867	20.1%
**IMDQ4**	589,622	17.2%	112,731	17.0%	10,827,628	19.8%
**IMDQ5**	558,031	16.2%	100,134	15.1%	10,626,384	19.4%
**Total**	3,434,680	100.0%	662,977	100.0%	54,786,327	100.0%

Apart from the estimated case proportion, distribution of individuals by IMDQ is used in calculations of equity measures.

Meningococcal carriage

The previous study by Cleary et al. [39] provided an association of the deprivation index and prevalence of meningococcal carriage in England. In this study children (3-18 y.o.a.) in West Cumbria were tested for meningococcal carriage of meningococci serogroup B. The association was reported only for schoolchildren. However, the deprivation index reflects the status of a small areas mainly with regards to factors determined by adults residing in the area: income, employment, education, crime, among others. Thus, it is reasonable to assume that similar association between deprivation and meningococcal carriage holds for other age groups. The data sourced from the Cleary et al. [39] were adjusted for the original analysis settings as follows (see Table S12):

- MenB and MenACWY carriage were estimated as a product of the prevalence of *N. meningitidis* carriage observed in Cleary et al. [39] and proportion of MenB and MenACWY carriers among all Men carriers used in the original analysis (28.38% and 26.73%, respectively).
- The obtained values were further adjusted to reflect MenB carriage in children ≤5 y.o.a., to ensure that computed weighted average for total population corresponds to the original model settings (1.5% and 1.4% MenB and MenACWY carriers in <5 y.o.a. individuals, respectively).

It should be noted that the data from Cleary et al. [39] may be not representative of the total population due to the relatively small size of the tested sample (467 individuals, 65 carriers), and no stratification by age. Furthermore, the data suggested higher carriage in IMDQ3 compared with IMDQ2, which was considered counterintuitive, and could be due to the small sample size. Thus, for the reference case analysis, normalized data were used, where carriage for IMDQ 3 was estimated as an average of the estimates for IMDQ2 and IMDQ4, further adjusted to reflect population <5 y.o.a., as described above also considering expert insights.

The input values are provided in Table S12.

Table S12. Prevalence of meningococcal carriage according to IMDQ.

	Number of carriers in a sample (n / N)	Prevalence of *N. meningitidis* carriage	MenB carriage (28.38%)	MenB carriage, for <5 y.o.a.	Prevalence of *N. meningitidis* carriage, normalized	MenB carriage, normalized	MenB carriage, normalized, for <5 y.o.a. ref. case
**IMDQ1**	39 / 220	17.7%	5.0%	2.3%	17.7%	5.0%	2.4%
**IMDQ2**	9 / 86	10.5%	3.0%	1.3%	10.5%	3.0%	1.4%
**IMDQ3**	11 / 82	13.4%	3.8%	1.7%	9.5% (9+5)/(86+61)	2.7%	1.3%
**IMDQ4**	5 / 61	8.2%	2.3%	1.1%	8.2%	2.3%	1.1%
**IMDQ5**	1 / 18	5.6%	1.6%	0.7%	5.6%	1.6%	0.7%
**Total**	65 / 467	13.9%	3.3%	1.5%	62 / 459	3.1%	1.5%

MenACWY carriage for < 5 y.o.a. in IMDQ1, 2, 3, 4 and 5 was estimated at 2.2%, 1.3%, 1.2%, 1.0% and 0.7% for the reference case

Vaccine effectiveness and coverage

The same estimates for the vaccine effectiveness and duration of protection were applied for all strata, in line with the original model settings [35], as provided in Table S13.

Table S13. Vaccine effectiveness and persistence duration.

Dose	Effectiveness against MenB (direct protection)	Effectiveness against MenACWY (cross-protection)*	Persistence duration (months)
**Dose 1**	0%	62%	33
**Dose 2**	79%	62%	33
**Dose 3**	79%	32%	38

* conditional on the vaccinated individual being fully protected against MenB

In absence of evidence, it was deemed reasonable to assume the same vaccine efficacy and duration of protection for all strata, while the available data suggest that coverage varies. The recent study [49] reported the odds ratios of vaccination against invasive meningococcal disease within the 1st month following the recommended time window, according to IMDQ. The odds ratios by dose are provided in Table S14.

Table S14. Odds ratios of MenB vaccination by IMDQ.

	Odds ratio		
	Dose 1	Dose 2	Dose 3
**IMDQ1 : reference quintile**	1	1	1
**IMDQ2**	1 *	1.23	1.22
**IMDQ3**	1 *	1.41	1.32
**IMDQ4**	1 *	1.45	1.37
**IMDQ5**	1 **	1.6	1.43

* – Not reported by the source study [49]; as a conservative assumption, the same coverage was applied for all IMDQ.
** – Reported value is 0.81, which will not be applied for this analysis due to incompleteness of data for the 1st dose.

Estimates of coverage by stratum were derived using these odds ratios and the proportion of individuals in IMDQ (Table S11). Due to the lack of data for some of strata for the first dose, no difference in coverage was assumed.

The coverages by dose and stratum were based on the provided odds ratios, and the data on the coverage for the total population. The coverage in the total population could be represented as a weighted average of coverage in strata, which can be expressed by the following equation:

$$Coverage_{Total} = \sum_{i=1}^{5} p_i \left(1 + \left(\frac{1 - Coverage_1}{OR_i \times Coverage_1}\right)\right)^{-1},$$

where $Coverage_{Total}$ is the general population coverage for a given dose, $Coverage_1$ is the reference group coverage for a given dose, OR_i is an odds ratio for i-th stratum, and p_i is a proportion of i-th stratum in population.

This formula was used to obtain coverage in the 1st (reference) quintile. For the other quintiles coverage was estimated as follows:

$$Coverage_i = \left(\frac{OR_i \times Coverage_1}{1 - Coverage_1}\right) \div \left(1 + \frac{OR_i \times Coverage_1}{1 - Coverage_1}\right)$$

The final estimates of coverage by stratum and dose are provided in Table S15.

Table S15. Vaccine coverage by dose and IMD.

	Vaccine coverage		
	Dose 1	Dose 2	Dose 3
**IMDQ1 : reference quintile**	95.3%	91.2%	84.2%
**IMDQ2**	95.3%	92.7%	86.7%
**IMDQ3**	95.3%	93.6%	87.6%
**IMDQ4**	95.3%	93.7%	88.0%
**IMDQ5**	95.3%	94.3%	88.4%
**Total population**	95.3%	92.9%	86.7%

The stratified coverage was used in a reference case analysis

Estimated case proportions

An average effective coverage was computed by stratum for the timeframe of 5 years.

Example of calculations for the IMDQ 1 is provided in Table S16, Figure S5 presents a plot of the effective coverage function for the derived data.

Table S16. Estimated case proportion for IMDQ 1, over 5-year timeframe.

Age, months	Dose	Effective coverage (EC)	Protection function *	EC at time *t*	EC over time, (area under the curve, cumulative),
**2**	D1	$95.3\% * 0\% = 0\%$	$\exp\left(-\frac{t-2}{33}\right)$	$0\% * \exp\left(-\frac{t-2}{33}\right)$	$\int_2^4 EC(t)dt = 0\%$
**4**	D2	$91.2\% * 79.0\%$ $= 72.0\%$	$\exp\left(-\frac{t-4}{33}\right)$	$72.0\% * \exp\left(-\frac{t-4}{33}\right)$	$72.0\% \int_4^{12} \exp\left(-\frac{t-4}{33}\right) dt =$ $\frac{72.0\%}{33}\left(1 - \exp\left(-\frac{12-4}{33}\right)\right) = 512\%$
**12**	D3	$84.2\% * 79.0\%$ $= 66.5\%$	$\exp\left(-\frac{t-12}{38}\right)$	$66.5\% * \exp\left(-\frac{t-12}{38}\right)$	$66.5\% \int_{12}^{60} \exp\left(-\frac{t-12}{38}\right) dt =$ $\frac{66.5\%}{38}\left(1 - \exp\left(-\frac{60-12}{38}\right)\right) = 1813\%$
**Total EC**		$0 + 512 + 1813 = 2325\%$			
**Average EC**		$2325/60 = 38.7\%$			
**Estimated case proportion****		$\frac{25.9\% \times 17.7\% \times (1 - 38.7\%)}{0.071^{***}} = 35.3\%$			

* EC, Effective coverage computed as $Coverage \times Effectiveness$.
** Exponential waning was assumed in line with the original model.
*** Sum of all raw case proportions by stratum.

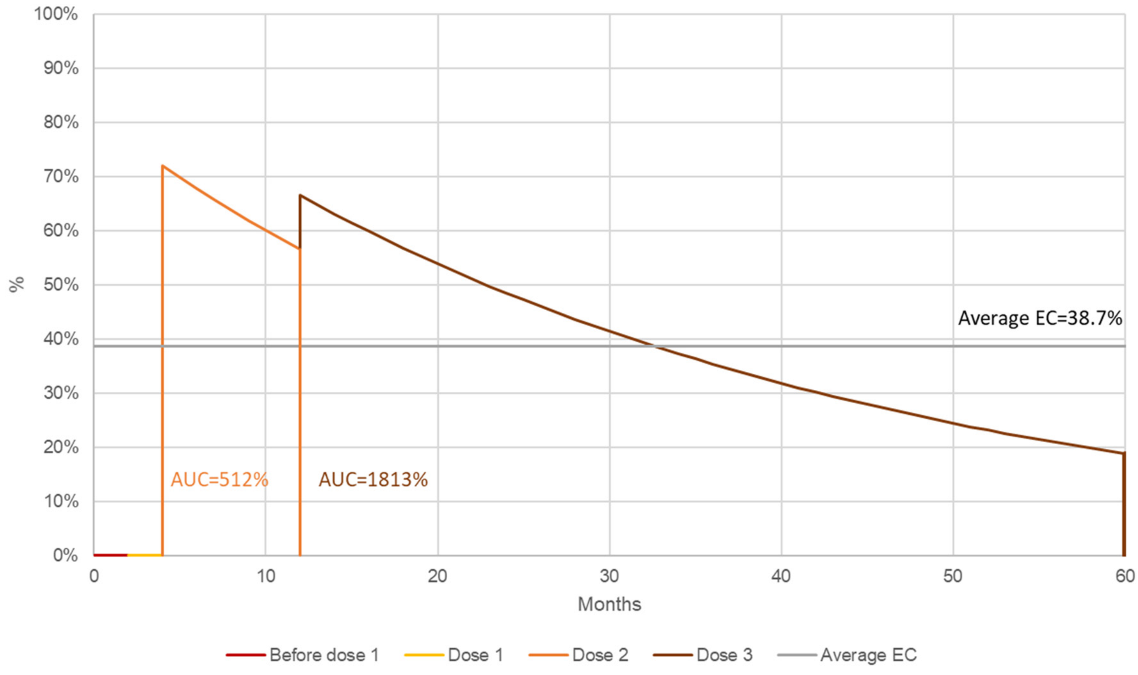

Figure S5. Vaccine effectiveness over time and the average effectiveness

AUC – Area under the curve (cumulative measure). EC – Effective coverage.
Exponential waning and 100-year model horizon were assumed in line with the original model.

Estimated case proportions for all strata are provided in Table S17.

Table S17. Estimated case proportions by IMDQ, reference case.

	Estimated case proportions		
	No vaccination *	4CMenB, direct protection against MenB	4CMenB, cross-protection against MenACWY
**IMDQ1**	41.8%	42.3%	41.9%
**IMDQ2**	20.9%	20.8%	20.9%
**IMDQ3**	16.3%	16.1%	16.2%
**IMDQ4**	12.8%	12.6%	12.8%
**IMDQ5**	8.2%	8.1%	8.2%

* The same estimated case proportion was applied for MenOther cases in 4CMenB arm, as no efficacy against these serogroups was assumed.

Life expectancy

The original model considers two types of inputs concerning mortality: age-specific death rates (the DTM input) and age-specific life expectancy (the decision tree input). In the model update only the decision tree input was changed following the methodology which does not consider DTM modification. General population life expectancy in reference case analysis was adjusted in the model before its use in the decision tree. Adjustment was made to account for reduced life expectancy following invasive meningococcal disease, in line with the original model.

For the reference case analysis, strata-specific data for the life expectancy were used. Life expectancy according to IMD decile by 5-year age groups was sourced from the data published by the Office for National Statistics, for 2014–2016 [50]. The data were adapted for this analysis: aggregated by quintile and gender, and redistributed for 1-year age groups, assuming the linear reduction of life expectancy for each year until 90. The exponential decay observed in the total population was applied for the individuals aged above 90.

The final estimates are provided in Table S18.

Table S18. Life expectancy.

Age	Life expectancy					
	IMDQ1	IMDQ2	IMDQ3	IMDQ4	IMDQ5	Total population
**0**	77.2	80.0	81.7	82.9	84.1	81.2
**1**	76.7	79.3	81.0	82.1	83.4	80.5
**2**	75.7	78.4	80.0	81.1	82.4	79.5
**3**	74.7	77.4	79.0	80.1	81.4	78.5
**4**	73.7	76.4	78.0	79.1	80.4	77.5
**5**	72.7	75.4	77.0	78.1	79.4	76.5
**6**	71.7	74.4	76.0	77.1	78.4	75.6
**7**	70.7	73.4	75.0	76.1	77.4	74.6
**8**	69.8	72.4	74.0	75.1	76.4	73.6
**9**	68.8	71.4	73.0	74.1	75.4	72.6
**10**	67.8	70.4	72.1	73.2	74.4	71.6
**11**	66.8	69.4	71.1	72.2	73.4	70.6

Age	Life expectancy					
	IMDQ1	IMDQ2	IMDQ3	IMDQ4	IMDQ5	Total population
**12**	65.8	68.5	70.1	71.2	72.4	69.6
**13**	64.8	67.5	69.1	70.2	71.4	68.6
**14**	63.8	66.5	68.1	69.2	70.4	67.6
**15**	62.8	65.5	67.1	68.2	69.4	66.6
**16**	61.8	64.5	66.1	67.2	68.4	65.6
**17**	60.9	63.5	65.1	66.2	67.5	64.6
**18**	59.9	62.5	64.1	65.2	66.5	63.6
**19**	58.9	61.5	63.1	64.2	65.5	62.6
**20**	58.0	60.6	62.1	63.2	64.5	61.7
**21**	57.0	59.6	61.2	62.3	63.5	60.7
**22**	56.0	58.6	60.2	61.3	62.5	59.7
**23**	55.1	57.6	59.2	60.3	61.6	58.8
**24**	54.1	56.7	58.2	59.3	60.6	57.8
**25**	53.1	55.7	57.3	58.4	59.6	56.8
**26**	52.1	54.7	56.3	57.4	58.6	55.8
**27**	51.2	53.7	55.3	56.4	57.7	54.9
**28**	50.2	52.7	54.3	55.4	56.7	53.9
**29**	49.2	51.7	53.4	54.5	55.7	52.9
**30**	48.2	50.8	52.4	53.5	54.8	51.9
**31**	47.3	49.8	51.4	52.5	53.8	51.0
**32**	46.3	48.8	50.4	51.5	52.8	50.0
**33**	45.3	47.8	49.5	50.6	51.9	49.0
**34**	44.4	46.9	48.5	49.6	50.9	48.1
**35**	43.4	45.9	47.5	48.6	49.9	47.1
**36**	42.5	44.9	46.6	47.6	48.9	46.1
**37**	41.5	44.0	45.6	46.7	47.9	45.2
**38**	40.6	43.0	44.6	45.7	47.0	44.2
**39**	39.6	42.1	43.7	44.7	46.0	43.3
**40**	38.7	41.1	42.7	43.8	45.0	42.3
**41**	37.8	40.2	41.8	42.8	44.0	41.4
**42**	36.9	39.3	40.8	41.8	43.1	40.4
**43**	36.0	38.3	39.9	40.9	42.1	39.4
**44**	35.1	37.4	38.9	39.9	41.1	38.5
**45**	34.2	36.5	38.0	39.0	40.1	37.6
**46**	33.3	35.5	37.0	38.0	39.2	36.6
**47**	32.4	34.6	36.1	37.1	38.2	35.7

Age	Life expectancy					
	IMDQ1	IMDQ2	IMDQ3	IMDQ4	IMDQ5	Total population
**48**	31.5	33.7	35.2	36.1	37.3	34.8
**49**	30.6	32.8	34.2	35.2	36.3	33.9
**50**	29.7	31.8	33.3	34.2	35.3	32.9
**51**	28.9	31.0	32.4	33.3	34.4	32.0
**52**	28.0	30.1	31.5	32.4	33.5	31.1
**53**	27.1	29.2	30.5	31.4	32.5	30.2
**54**	26.3	28.3	29.6	30.5	31.6	29.3
**55**	25.4	27.4	28.7	29.6	30.6	28.4
**56**	24.6	26.5	27.8	28.7	29.7	27.5
**57**	23.8	25.7	26.9	27.8	28.8	26.6
**58**	23.0	24.8	26.1	26.9	27.9	25.7
**59**	22.1	24.0	25.2	26.0	27.0	24.9
**60**	21.3	23.1	24.3	25.1	26.1	24.0
**61**	20.6	22.3	23.5	24.2	25.2	23.2
**62**	19.8	21.5	22.6	23.3	24.3	22.3
**63**	19.1	20.7	21.8	22.5	23.4	21.5
**64**	18.3	19.9	20.9	21.6	22.5	20.7
**65**	17.6	19.1	20.1	20.8	21.7	19.9
**66**	16.9	18.3	19.3	19.9	20.8	19.0
**67**	16.2	17.5	18.5	19.1	20.0	18.2
**68**	15.5	16.8	17.7	18.3	19.1	17.4
**69**	14.8	16.0	16.9	17.5	18.3	16.7
**70**	14.1	15.3	16.1	16.6	17.4	15.9
**71**	13.5	14.6	15.3	15.9	16.7	15.1
**72**	12.9	13.9	14.6	15.1	15.9	14.4
**73**	12.2	13.2	13.9	14.4	15.1	13.7
**74**	11.6	12.5	13.1	13.6	14.3	13.0
**75**	11.0	11.8	12.4	12.8	13.5	12.3
**76**	10.5	11.2	11.8	12.2	12.8	11.6
**77**	9.9	10.6	11.1	11.5	12.1	11.0
**78**	9.4	10.0	10.5	10.8	11.4	10.3
**79**	8.9	9.4	9.8	10.1	10.7	9.7
**80**	8.3	8.8	9.2	9.5	10.0	9.1
**81**	7.9	8.3	8.6	8.9	9.4	8.5
**82**	7.4	7.8	8.1	8.4	8.8	7.9
**83**	7.0	7.4	7.6	7.8	8.3	7.4

Age	Life expectancy					
	IMDQ1	IMDQ2	IMDQ3	IMDQ4	IMDQ5	Total population
**84**	6.5	6.9	7.1	7.2	7.7	6.9
**85**	6.1	6.4	6.5	6.7	7.1	6.4
**86**	5.8	6.0	6.1	6.3	6.7	6.0
**87**	5.4	5.7	5.7	5.9	6.3	5.5
**88**	5.1	5.3	5.3	5.5	5.8	5.1
**89**	4.8	4.9	5.0	5.1	5.4	4.8
**90**	4.4	4.6	4.6	4.7	5.0	4.4
**91**	4.1	4.2	4.2	4.3	4.6	4.1
**92**	3.8	3.9	3.9	4.0	4.3	3.8
**93**	3.5	3.6	3.6	3.7	3.9	3.5
**94**	3.2	3.3	3.3	3.4	3.6	3.3
**95**	3.0	3.1	3.1	3.1	3.4	3.1
**96**	2.7	2.8	2.8	2.9	3.1	2.9
**97**	2.5	2.6	2.6	2.7	2.9	2.7
**98**	2.3	2.4	2.4	2.5	2.7	2.5
**99**	2.2	2.2	2.2	2.3	2.5	2.3

Utility inputs

The study by Collins et al. [52] presents QALE (quality adjusted life expectancy) and LE for males and females from North West of England according to IMDQ. Average utilities for males and females according to quintiles of deprivation were calculated as ratio of respective QALE and LE values, subsequently pooled in a single estimate, regardless of gender.

Table S19. Utilities according to IMDQ.

	IMDQ1	IMDQ2	IMDQ3	IMDQ4	IMDQ5	Total population
	Males					
**QALE**	64.75	70.92	75.58	76.97	81.53	-
**LE**	74.87	78.53	83.42	85.52	88.9	-
**Calculated average utility (QALE/LE)**	0.86	0.90	0.91	0.90	0.92	-
	Females					
**QALE**	66.16	69.86	74.68	78.14	80.68	-
**LE**	78.35	80.56	85.06	88.22	91.22	-
**Calculated average utility (QALE/LE)**	0.84	0.87	0.88	0.89	0.88	-
	Calculated weighted* utilities					
**Calculated average baseline utility**	0.854	0.885	0.892	0.893	0.901	0.885**
**Adjustment factor – ratio of kth IMDQ utility / average utility for the total population**	0.966	1.000	1.008	1.009	1.018	-

* Males 49.34%, females 50.66%, based on ONS Mid-2015 estimates
** Computed using proportions for the total population, all ages (see Table S11).

In order to apply the derived distribution of utilities, an adjustment factor was computed, as a ratio of computed utility for each IMDQ to the estimated weighted average for population. Finally, these adjustment factors were applied to the utilities by age used by the original model strata-specific data used in the reference case analysis are presented in Table S20.

Table S20. Age-specific average utility by strata.

Age	Utility					
	IMDQ1	IMDQ2	IMDQ3	IMDQ4	IMDQ5	Total population
**0**	0.939	0.972	0.980	0.981	0.989	0.972
**1**	0.939	0.972	0.980	0.981	0.989	0.972
**2**	0.939	0.972	0.980	0.981	0.989	0.972
**3**	0.939	0.972	0.980	0.981	0.989	0.972
**4**	0.939	0.972	0.980	0.981	0.989	0.972
**5**	0.939	0.972	0.980	0.981	0.989	0.972
**6**	0.939	0.972	0.980	0.981	0.989	0.972
**7**	0.939	0.972	0.980	0.981	0.989	0.972

Age	Utility					
	IMDQ1	IMDQ2	IMDQ3	IMDQ4	IMDQ5	Total population
**8**	0.939	0.972	0.980	0.981	0.989	0.972
**9**	0.939	0.972	0.980	0.981	0.989	0.972
**10**	0.939	0.972	0.980	0.981	0.989	0.972
**11**	0.939	0.972	0.980	0.981	0.989	0.972
**12**	0.939	0.972	0.980	0.981	0.989	0.972
**13**	0.939	0.972	0.980	0.981	0.989	0.972
**14**	0.939	0.972	0.980	0.981	0.989	0.972
**15**	0.939	0.972	0.980	0.981	0.989	0.972
**16**	0.939	0.972	0.980	0.981	0.989	0.972
**17**	0.939	0.972	0.980	0.981	0.989	0.972
**18**	0.939	0.972	0.980	0.981	0.989	0.972
**19**	0.939	0.972	0.980	0.981	0.989	0.972
**20**	0.939	0.972	0.980	0.981	0.989	0.972
**21**	0.939	0.972	0.980	0.981	0.989	0.972
**22**	0.939	0.972	0.980	0.981	0.989	0.972
**23**	0.939	0.972	0.980	0.981	0.989	0.972
**24**	0.939	0.972	0.980	0.981	0.989	0.972
**25**	0.939	0.972	0.980	0.981	0.989	0.972
**26**	0.939	0.972	0.980	0.981	0.990	0.972
**27**	0.939	0.972	0.980	0.981	0.990	0.972
**28**	0.939	0.973	0.980	0.981	0.990	0.972
**29**	0.939	0.973	0.980	0.981	0.990	0.973
**30**	0.939	0.973	0.980	0.981	0.990	0.973
**31**	0.939	0.973	0.980	0.982	0.990	0.973
**32**	0.940	0.973	0.981	0.982	0.990	0.973
**33**	0.940	0.973	0.981	0.982	0.990	0.973
**34**	0.940	0.973	0.981	0.982	0.990	0.973
**35**	0.939	0.972	0.980	0.981	0.990	0.972
**36**	0.938	0.972	0.979	0.980	0.989	0.972
**37**	0.938	0.971	0.979	0.980	0.988	0.971
**38**	0.937	0.970	0.978	0.979	0.988	0.970
**39**	0.936	0.970	0.977	0.978	0.987	0.970
**40**	0.936	0.969	0.977	0.978	0.986	0.969
**41**	0.935	0.968	0.976	0.977	0.985	0.968
**42**	0.934	0.968	0.975	0.976	0.985	0.967
**43**	0.934	0.967	0.974	0.976	0.984	0.967

Age	Utility<br>IMDQ1	IMDQ2	IMDQ3	IMDQ4	IMDQ5	Total population
**44**	0.933	0.966	0.974	0.975	0.983	0.966
**45**	0.931	0.964	0.972	0.973	0.981	0.964
**46**	0.929	0.962	0.969	0.971	0.979	0.962
**47**	0.927	0.960	0.967	0.968	0.977	0.960
**48**	0.925	0.958	0.965	0.966	0.975	0.958
**49**	0.923	0.956	0.963	0.964	0.973	0.956
**50**	0.921	0.954	0.961	0.962	0.970	0.953
**51**	0.919	0.951	0.959	0.960	0.968	0.951
**52**	0.917	0.949	0.957	0.958	0.966	0.949
**53**	0.915	0.947	0.955	0.956	0.964	0.947
**54**	0.913	0.945	0.953	0.954	0.962	0.945
**55**	0.910	0.943	0.950	0.951	0.960	0.943
**56**	0.908	0.941	0.948	0.949	0.957	0.940
**57**	0.906	0.938	0.946	0.947	0.955	0.938
**58**	0.904	0.936	0.943	0.944	0.953	0.936
**59**	0.902	0.934	0.941	0.942	0.950	0.934
**60**	0.899	0.931	0.939	0.940	0.948	0.931
**61**	0.897	0.929	0.936	0.937	0.946	0.929
**62**	0.895	0.927	0.934	0.935	0.943	0.927
**63**	0.893	0.924	0.932	0.933	0.941	0.924
**64**	0.890	0.922	0.929	0.930	0.938	0.922
**65**	0.887	0.919	0.926	0.927	0.935	0.919
**66**	0.884	0.916	0.923	0.924	0.932	0.916
**67**	0.882	0.913	0.920	0.921	0.929	0.913
**68**	0.879	0.910	0.917	0.918	0.926	0.910
**69**	0.876	0.907	0.914	0.915	0.923	0.907
**70**	0.873	0.904	0.911	0.912	0.920	0.903
**71**	0.870	0.900	0.908	0.908	0.916	0.900
**72**	0.867	0.897	0.904	0.905	0.913	0.897
**73**	0.864	0.894	0.901	0.902	0.910	0.894
**74**	0.861	0.891	0.898	0.899	0.907	0.891
**75**	0.856	0.886	0.893	0.894	0.902	0.886
**76**	0.850	0.881	0.888	0.889	0.896	0.881
**77**	0.845	0.876	0.882	0.883	0.891	0.875
**78**	0.840	0.870	0.877	0.878	0.886	0.870
**79**	0.835	0.865	0.872	0.873	0.880	0.865

Age	Utility					
	IMDQ1	IMDQ2	IMDQ3	IMDQ4	IMDQ5	Total population
**80**	0.830	0.860	0.867	0.868	0.875	0.860
**81**	0.825	0.855	0.861	0.862	0.870	0.855
**82**	0.820	0.850	0.856	0.857	0.865	0.849
**83**	0.815	0.844	0.851	0.852	0.859	0.844
**84**	0.810	0.839	0.846	0.847	0.854	0.839
**85**	0.810	0.839	0.846	0.847	0.854	0.839
**86**	0.800	0.829	0.835	0.836	0.843	0.829
**87**	0.795	0.824	0.830	0.831	0.838	0.823
**88**	0.790	0.818	0.825	0.826	0.833	0.818
**89**	0.790	0.818	0.825	0.826	0.833	0.818
**90**	0.780	0.808	0.814	0.815	0.822	0.808
**91**	0.775	0.803	0.809	0.810	0.817	0.803
**92**	0.770	0.798	0.804	0.805	0.812	0.797
**93**	0.770	0.798	0.804	0.805	0.812	0.797
**94**	0.760	0.787	0.793	0.794	0.801	0.787
**95**	0.755	0.782	0.788	0.789	0.796	0.782
**96**	0.750	0.777	0.783	0.784	0.790	0.777
**97**	0.750	0.777	0.783	0.784	0.790	0.777
**98**	0.740	0.766	0.772	0.773	0.780	0.766
**99**	0.735	0.761	0.767	0.768	0.775	0.761

The literature data suggest that quality of life as well as life expectancy varies among strata [38,50]. However, due to the model approach to utility calculations (QALY loss = disutility * average health state utility), stratification of inputs related to baseline utilities may lead to different QALY losses for the different socioeconomic strata having the same sequelae. Such settings would result in disproportionally higher disutility for some strata, which could be not in line with the general equity concept and could contribute to the biased results.

Cost inputs

Direct costs

No evidence was identified to support an assumption on variation of the direct medical costs of meningococcal disease by IMDQ. Indeed, considering that universal healthcare coverage is provided by the NHS in the UK, it is not expected that direct medical costs attributable to payer vary by socioeconomic status. Although from a patient perspective, in particular long term survivors from less deprived classes may have better access to private health insurance or sick pay (including for taking care of children with invasive meningococcal disease). This study focusses on societal and payer perspective, thus the reference case analysis included the same set of inputs for direct costs as the original model. However, the model adaptation considers a possibility for stratification of these inputs, as such data may be available for other countries, or for the UK setting in the future.

Indirect costs

In the original analysis cost of productivity loss was applied during the acute phase of the disease, in case of death related to invasive meningococcal disease and for cases with work disabilities due to long-term sequelae as well as for parents staying at home during the acute phase and for a long term to take care of their child with severe sequelae. Given that children and parents can be reasonably assigned to the same socioeconomic strata (an assumption similar to the study of Cleary et al. [39]), in this analysis it was assumed that in general caregivers are from the same socioeconomic group as the actual patients.

Socioeconomic status is directly related to the income, thus it has an impact on the cost of the lost productivity. Annual income by age and IMDQ was estimated using the following data:

- Annual income by age from the original 4CMenB model,
- Population by age and IMDQ for England 2015 published by the Office for National Statistics (ONS) [48],
- Domains of deprivation statistics provided by Ministry of Housing, Communities & Local Government [51],
- Income distribution by percentiles estimated from the Survey of Personal Incomes (before tax) [52].

The annual income by age sourced from the original model was redistributed to obtain the data stratified by age and IMDQ with the following calculation steps:

1. Income deciles estimated from the Survey of Personal Incomes were matched to income deciles for each Lower-layer Super Output Area (LSOA), provided by Ministry of Housing, Communities & Local Government, to obtain the corresponding income,
2. Total income by LSOA was estimated by multiplying the matched income by the number of individuals in this LSOA.
3. The total income obtained in step 2 was matched to the IMD deciles, by LSOA,
4. Weighted average income by IMD decile was computed (LSOA aggregated),
5. Weighted average income by IMDQ was computed, grouping the data by decile (from step 4) pairwise,
6. Proportions of the total income attributable to each IMDQ were estimated,
7. Total annual income by age was computed, using the data from the original model and number of individuals by age,
8. Total annual income by IMDQ for each age group was estimated using the proportions computed in the step 6,
9. Average annual income by age and IMDQ was computed by dividing the values obtained in step 8 by the number of individuals in each IMDQ and age group.

The obtained data are provided in Table S21.

Table S21. Annual income by age and IMDQ.

	IMDQ1	IMDQ2	IMDQ3	IMDQ4	IMDQ5	Total population
**<15**	0	0	0	0	0	0
**15-19**	3,635	5,954	8,452	12,740	25,885	11,135
**20-24**	3,407	4,955	8,090	13,795	34,612	11,135
**25-29**	7,258	10,877	17,564	31,691	85,966	24,828
**30-34**	7,563	11,187	17,413	30,607	80,428	24,828

	IMDQ1	IMDQ2	IMDQ3	IMDQ4	IMDQ5	Total population
**35-39**	10,218	15,019	21,677	34,860	78,947	30,358
**40-44**	11,264	16,481	21,797	32,655	68,187	30,358
**45-49**	11,853	17,174	21,796	31,955	66,599	30,618
**50-54**	12,434	17,565	21,458	31,260	64,979	30,618
**55-59**	10,874	15,127	18,053	26,318	55,363	26,126
**60-64**	11,568	15,519	17,701	25,534	54,244	26,126
**65-69**	10,979	13,689	14,734	20,601	43,167	22,038
**70-74**	11,210	13,837	14,672	20,447	42,760	22,038
**75-79**	10,698	13,622	14,789	20,857	43,358	22,038
**80-84**	10,862	13,553	14,800	20,922	42,951	22,038
**85+**	10,927	13,282	14,621	20,945	43,900	22,038

Equity inputs

Inequality aversion parameter for the Atkinson's and Kolm-Pollak's EDEH were considered equal to 10.95 and 0.15 respectively, in line with the recent UK studies [33,43]. These parameters were elicited in a study by Robson et al., using the data from the online survey (n = 244). The obtained values indicate a substantial concern for health inequality among the English general public, as the majority of respondents (81.51%) expressed their preference to sacrifice gains in total health to reduce inequality [43].

Quality of life adjustment factor (QAF)

Quality of life adjustment factor was applied in the original analysis to scale up QALYs, which reflects society's preferences for the prevention of rare but severe diseases. Following the Joint Committee on Vaccination and Immunisation (JCVI) recommendation [53], a QAF equal to 3 was used.

During the expert consultation it was pointed out that inclusion of modifiers accounting for severity and equity in the same analysis could be criticized, as it could lead to the overestimation of the equity impact. However, to the best of our knowledge at present, we are not aware of any published conclusive data or studies evaluating whether society may not only have a preference for prevention of severe disease as well as a preference to prevent disease in the most deprived [43,54], but also a 'joint' preference for the prevention of severe disease in the most deprived. To explore the impact of these potential preferences and the related impact of the uncertainty on the results, two reference cases were explored in the adapted analysis, with QAF equal to 3 (baseline, in alignment with the original model and publication of Beck et al. 2021 [29]) and QAF equal to 1, i.e., without QALYs adjustment for severity.

7. Results

7.1. Reference case results

Reference case results are presented for outcome distributions, equity impact and full DCEA. All results are presented for lifetime horizon and 1.5% discount rate in alignment with the underlying model of Beck et al. 2021 [29] to ensure comparability between analyses.

Distributions

Number of MenB cases

Table S22 and Figure S6 below provide the number of MenB cases for no vaccination and 4CMenB arm for each IMDQ, the total number of cases and SII. Of all prevented cases, 40.3% were among the most deprived IMDQ and 78.1% among the three most deprived IMDQs. SII was lower in 4CMenB arm, which implies that vaccination decreased inequality. The obtained values of SII were relatively small, which reflects low incidence of invasive meningococcal disease.

Similar trend was observed for MenB incidence rate, the number of long-term sequelae and deaths due to invasive meningococcal disease, since no difference in the probability of sequelae or death across IMDQ was assumed for this analysis.

Table S22. Reference case results - Distributions: Number of MenB cases (QAF = 3 and QAF = 1).

	IMDQ1	IMDQ2	IMDQ3	IMDQ4	IMDQ5	Total population	SII
**Per stratum/population**							
No vaccination	46,428	23,228	18,096	14,240	9,135	111,127	NA
4CMenB vaccination	34,404	16,924	13,097	10,279	6,570	81,274	NA
Incremental	12,024	6,305	4,999	3,961	2,564	29,853	NA
**Per individual**							
No vaccination	1.4E-03	8.1E-04	7.4E-04	6.3E-04	4.3E-04	8.5E-04*	1.1E-03
4CMenB vaccination	1.0E-03	5.9E-04	5.3E-04	4.6E-04	3.1E-04	6.2E-04*	8.0E-04
Incremental	3.6E-04	2.2E-04	2.0E-04	1.8E-04	1.2E-04	2.3E-04	-2.7E-04

NA, Not available
* Weighted average, given proportions of population by IMDQ

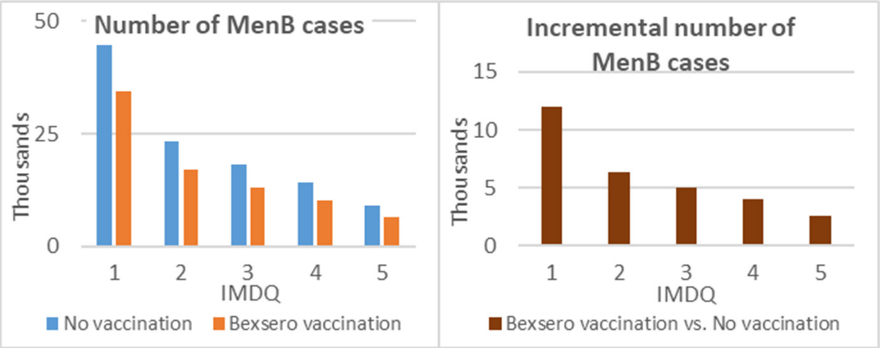

Figure S6. Reference case results - Distribution: Number of MenB cases per stratum (QAF = 3 and QAF = 1).

MenB incidence rate (per 100,000 persons)

Table S23 and Figure S7 below provide MenB incidence rate (per 100,000 persons) for no vaccination and 4CMenB arm for each IMDQ, the average incidence rate in population and SII.

Table S23. Reference case results - Distributions: MenB incidence rate (per 100,000 persons) (QAF = 3 and QAF = 1).

	IMDQ1	IMDQ2	IMDQ3	IMDQ4	IMDQ5	Total population	SII
No vaccination	3.06	1.81	1.64	1.41	0.96	1.89	2.38
4CMenB vaccination	2.27	1.32	1.19	1.02	0.69	1.39	1.79
Incremental	0.79	0.49	0.45	0.39	0.27	0.51	-0.59

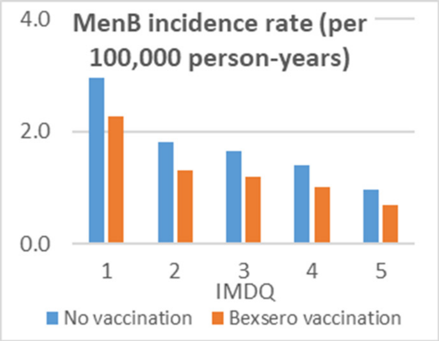

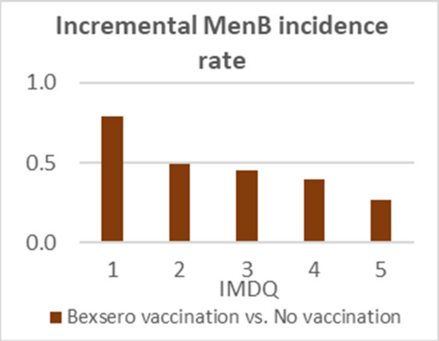

Figure S7. Reference case results - Distribution: MenB incidence rate per stratum (100,000 person-years, QAF = 3 and QAF = 1).

Number of long-term sequelae

Table S24 and Figure S8 below provide the number of long-term sequelae for no vaccination and 4CMenB arm for each IMDQ, the total number of sequelae and SII.

Table S24. Reference case results - Distributions: Number of long-term sequelae (QAF = 3 and QAF = 1).

	IMDQ1	IMDQ2	IMDQ3	IMDQ4	IMDQ5	Total population	SII
**Per stratum/population**							
No vaccination	25,911	12,963	10,099	7,947	5,098	62,019	NA
4CMenB vaccination	20,207	9,971	7,726	6,067	3,880	47,851	NA
Incremental	5,704	2,992	2,373	1,881	1,218	14,167	NA
**Per individual**							
No vaccination	7.7E-04	4.5E-04	4.1E-04	3.5E-04	2.4E-04	4.7E-04*	6.0E-04

4CMenB vaccination	6.0E-04	3.5E-04	3.1E-04	2.7E-04	1.8E-04	3.7E-04*	4.7E-04
Incremental	1.7E-04	1.0E-04	9.7E-05	8.4E-05	5.7E-05	1.1E-04	-1.3E-04

NA, Not available
* Weighted average, given proportions of population by IMDQ

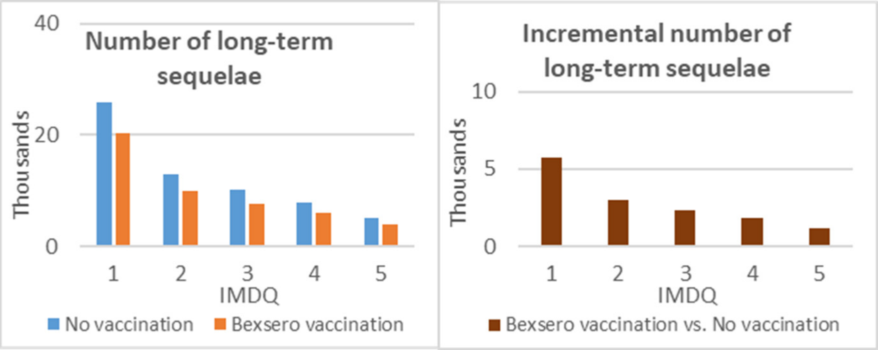

Figure S8. Reference case results - Distribution: Number of long-term sequelae per stratum (QAF = 3 and QAF = 1).

Number of deaths

Table S25 and Figure S9 below provide the number of deaths for no vaccination and 4CMenB arm for each IMDQ, the total number of deaths and SII.

Table S25. Reference case results - Distributions: Number of deaths (QAF = 3 and QAF = 1).

	IMDQ1	IMDQ2	IMDQ3	IMDQ4	IMDQ5	Total population	SII
**Per stratum/population**							
No vaccination	3,621	1,812	1,411	1,111	712	8,667	NA
4CMenB vaccination	3,110	1,538	1,193	937	599	7,377	NA
Incremental	511	274	219	174	113	1,290	NA
**Per individual**							
No vaccination	1.1E-04	6.3E-05	5.7E-05	4.9E-05	3.4E-05	6.6E-05*	8.3E-05
4CMenB vaccination	9.2E-05	5.4E-05	4.9E-05	4.2E-05	2.8E-05	5.6E-05*	7.2E-05
Incremental	1.5E-05	9.5E-06	8.9E-06	7.7E-06	5.3E-06	9.9E-06	-1.1E-05

NA, Not available
* Weighted average, given proportions of population by IMDQ

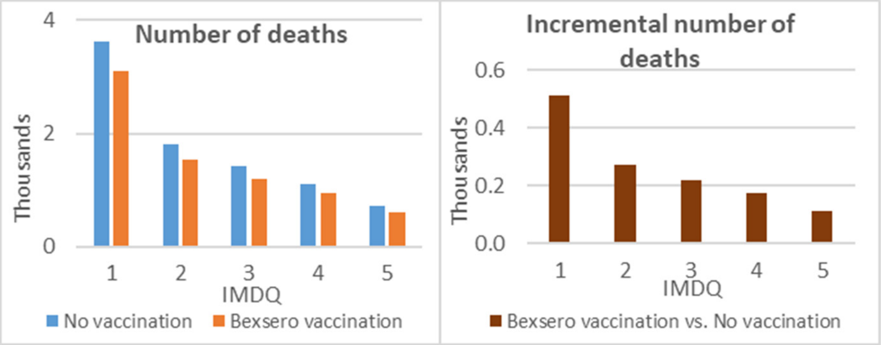

Figure S9. Reference case results - Distribution: Number of deaths per stratum (QAF = 3 and QAF = 1).

QALY loss

Table S26 and Table S27 below provide QALY loss for no vaccination and 4CMenB arm for each IMDQ, results for SII and NHB, for QAF=3 and QAF=1 respectively. Graphical representation of the obtained data is provided in Figure S10 and Figure S11.

The highest QALY loss was expected for IMDQ1, which is in line with the results for incidence, although the assumed differences in life expectancy and utility also had an impact on results. Notably, incremental QALYs and NHB indicate that vaccination is also the most beneficial for the most deprived quintile. For QAF=3 and payer perspective, NHB considering equal distribution of health opportunity costs is negative for less deprived IMDQ 2-5; in societal perspective NHB remains positive for IMDQ 1-3. For QAF=1, NHB is negative for all IMDQ in payer perspective, and positive only for IMDQ 1 in societal perspective.

In line with the results on the disease incidence, SII was lower in 4CMenB arm, indicating that vaccination decreases inequity in population, and the impact is higher for QAF=3 (QAF > 1 resulting in overall higher QALY losses due to MenB).

The obtained results are in line with the data for total QALYs.

Table S26. Reference case results (baseline) - Distributions: QALY loss (QAF = 3).

	IMDQ1	IMDQ2	IMDQ3	IMDQ4	IMDQ5	Total population	SII
**Per stratum/population**							
No vaccination	628,702	333,514	263,197	208,077	136,910	1,570,400	NA
4CMenB vaccination	488,495	255,938	201,150	158,812	104,226	1,208,621	NA
Incremental	140,207	77,576	62,047	49,265	32,684	361,779	NA
NHB, Payer perspective	40,804	-6,667	-10,068	-16,673	-29,721	-22,325	NA
NHB, Societal perspective	65,144	13,961	7,590	-527	-14,440	71,727	NA
**Per individual**							
No vaccination	0.019	0.012	0.011	0.009	0.006	0.012*	0.014

4CMenB vaccination	0.014	0.009	0.008	0.007	0.005	0.009*	0.011
Incremental	0.004	0.003	0.003	0.002	0.002	0.003	-0.003

NA, Not applicable
* Weighted average, given proportions of population by IMDQ

Table S27. Reference case results - Distributions: QALY loss (QAF = 1).

	IMDQ1	IMDQ2	IMDQ3	IMDQ4	IMDQ5	Total population	SII
**Per stratum/population**							
No vaccination	366,111	194,179	152,793	120,410	79,408	912,901	NA
4CMenB vaccination	291,026	152,471	119,532	94,104	61,880	719,013	NA
Incremental	75,085	41,709	33,260	26,306	17,528	193,888	NA
NHB, Payer perspective	-24,318	-42,534	-38,854	-39,632	-4,877	-190,215	NA
NHB, Societal perspective	22	-21,906	-21,196	-23,486	-29,596	-96,163	NA
**Per individual**							
No vaccination	0.011	0.007	0.006	0.005	0.004	0.007*	0.008
4CMenB vaccination	0.009	0.005	0.005	0.004	0.003	0.005*	0.006
Incremental	0.002	0.001	0.001	0.001	0.001	0.001	-0.002

NA, Not applicable
* Weighted average, given proportions of population by IMDQ

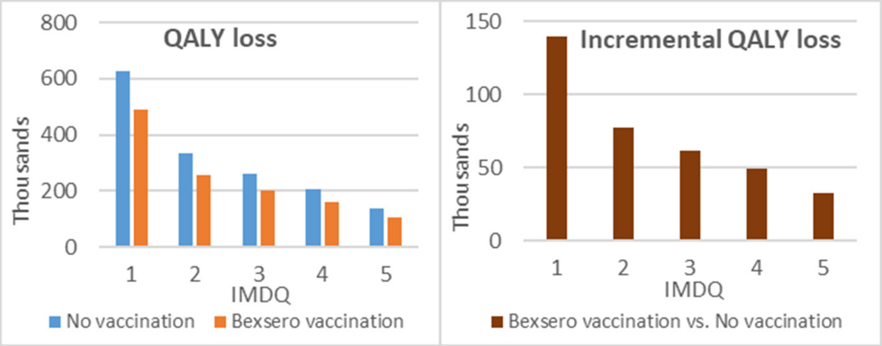

Figure S10. Reference case results (baseline) - Distribution: QALY loss (QAF = 3).

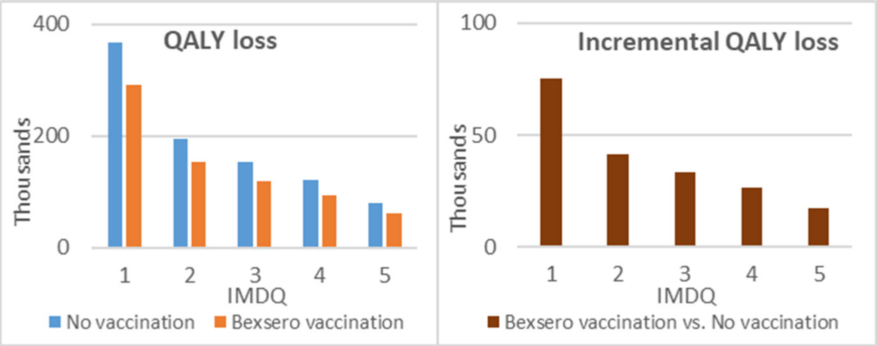

Figure S11. Reference case results - Distribution: QALY loss (QAF = 1).

Total QALYs

Total QALYs were introduced for this model adaptation to informing health equity analysis, in line with the published literature on DCEA [31-33,42] and expert opinion.

In Table S28 and Table S29 below total QALYs for no vaccination and 4CMenB arm for each IMDQ, total results and slope index are presented for QAF=1 and QAF=3 respectively. Graphical representation of the obtained data is provided in Figure S12 and Figure S13.

Table S28. Reference case results (baseline) - Distributions: Total QALYs (QAF = 3).

	IMDQ1	IMDQ2	IMDQ3	IMDQ4	IMDQ5	Total population	SII
**Per stratum/population**							
No vaccination	770,654,617	694,825,898	606,837,931	560,812,046	543,384,559	3,176,515,051	NA
4CMenB vaccination	770,794,824	694,903,474	606,899,978	560,861,311	543,417,243	3,176,876,830	NA
Incremental	140,207	77,576	62,047	49,265	32,684	361,779	NA
NHB, Payer perspective	40,804	-6,667	-10,068	-16,673	-29,721	-22,325	NA
NHB, Societal perspective	65,144	13,961	7,590	-527	-14,440	71,727	NA
**Per individual**							
No vaccination	22.764	24.218	24.708	24.973	25.567	24.282*	3.293
4CMenB vaccination	22.768	24.220	24.710	24.975	25.568	24.285*	3.290
Incremental	0.004	0.003	0.003	0.002	0.002	0.003	-0.003

NA, Not applicable
* Weighted average, given proportions of population by IMDQ

Table S29. Reference case results - Distributions: Total QALYs (QAF = 1).

	IMDQ1	IMDQ2	IMDQ3	IMDQ4	IMDQ5	Total population	SII
**Per stratum/population**							
No vaccination	770,917,208	694,965,233	606,948,336	560,899,712	543,442,061	3,177,172,550	NA
4CMenB vaccination	770,992,293	695,006,941	606,981,596	560,926,018	543,459,589	3,177,366,438	NA

Incremental	75,085	41,709	33,260	26,306	17,528	193,888	NA
NHB, Payer perspective	-24,318	-42,534	-38,854	-39,632	-44,877	-190,215	NA
NHB, Societal perspective	22	-21,906	-21,196	-23,486	-29,596	-96,163	NA
**Per individual**							
No vaccination	22.772	24.223	24.712	24.977	25.569	24.287*	3.287
4CMenB vaccination	22.774	24.224	24.714	24.978	25.570	24.289*	3.285
Incremental	0.002	0.001	0.001	0.001	0.001	0.001	-0.002

NA, Not applicable
* Weighted average, given proportions of population by IMDQ

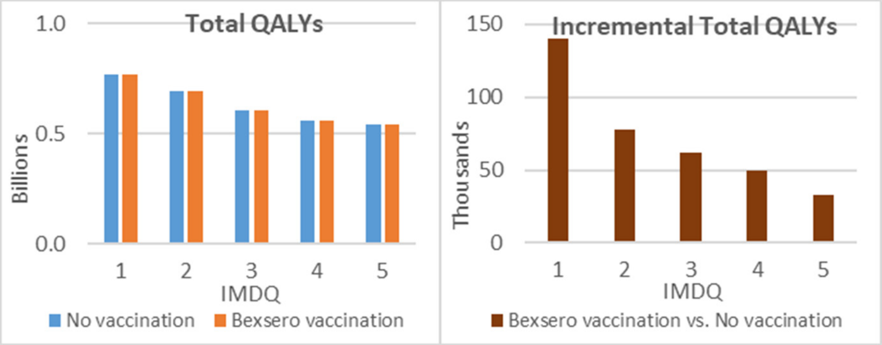

Figure S12. Reference case results (baseline)- Distribution: Total QALYs (QAF = 3).

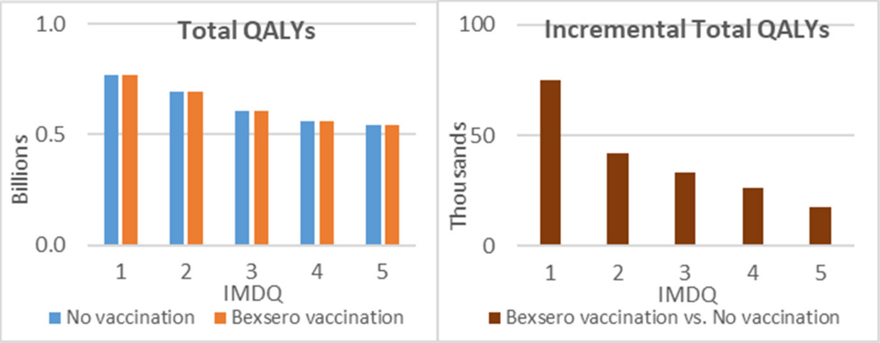

Figure S13. Reference case results - Distribution: Total QALYs (QAF = 1).

Costs

Table S30 and Table S31 below provide costs for no vaccination and 4CMenB arm for each IMDQ, total costs and SII, for societal and payer perspectives respectively.

Similarly to the health outcomes, SII computed for costs was lower in 4CMenB arm, indicating that vaccination decreases inequity in population. For both payer and societal perspective, the highest costs were expected for IMDQ 1. For the payer perspective there was a linear relationship between costs, which were declining from IMDQ 1 to IMDQ 5. For the societal perspective, costs in IMDQ 1 remained the highest, and decreased for IMDQ 2 to IMDQ 4. However, costs increased for IMDQ 5 comparing to preceding two quintiles, indicating non-linear relationship for the costs estimated from the societal perspective. Non-

linear relationship was also observed for incremental costs, which was due to the decreasing proportion of individuals (and disease cases) from IMDQ 1 to IMDQ 5, but increasing trend for the income.

Graphical representation of the obtained data is provided in Figure S14 and Figure S15 below.

Table S30. Reference case results - Distributions: Costs (QAF=3 and QAF=1), Payer perspective.

	IMDQ1	IMDQ2	IMDQ3	IMDQ4	IMDQ5	Total population	SII
**Per stratum/population**							
No vaccination	2,000,326,523	1,012,570,464	793,636,575	628,005,359	405,138,912	4,839,677,832	NA
4CMenB vaccination	3,761,515,466	2,706,981,560	2,272,367,499	2,009,882,239	1,771,005,729	12,521,752,493	NA
Incremental	1,761,188,943	1,694,411,096	1,478,730,923	1,381,876,881	1,365,866,818	7,682,074,661	NA
NMB (QAF=3)	816,075,441	-133,339,137	-201,356,749	-333,458,829	-594,415,652	-446,494,926	NA
NMB (QAF=1)	-486,363,877	-850,680,099	-777,086,394	-792,641,162	-897,534,389	-3,804,305,922	NA
**Per individual**							
No vaccination	59	35	32	28	19	37*	45
4CMenB vaccination	111	94	93	89	83	96*	31
Incremental	-52	-59	-60	-62	-64	-59	-14

NA, Not applicable
* Weighted average, given proportions of population by IMDQ

Table S31. Reference case results - Distributions: Costs (QAF=3 and QAF=1), Societal perspective.

	IMDQ1	IMDQ2	IMDQ3	IMDQ4	IMDQ5	Total population	SII
**Per stratum/population**							
No vaccination	4,282,427,161	2,444,933,539	2,154,703,800	2,192,088,305	2,549,343,148	13,623,495,952	NA
4CMenB vaccination	5,588,425,628	3,836,791,727	3,336,551,549	3,228,767,506	3,433,993,934	19,424,530,343	NA
Incremental	1,305,998,467	1,391,858,188	1,181,847,749	1,036,679,201	884,650,786	5,801,034,391	NA
NMB (QAF=3)	1,302,874,323	279,215,534	151,805,183	-10,545,857	-288,803,838	1,434,545,344	NA
NMB (QAF=1)	435,005	-438,125,428	-423,924,463	-469,728,190	-591,922,576	-1,923,265,652	NA
**Per individual**							
No vaccination	126	85	88	98	120	104*	61

	IMDQ1	IMDQ2	IMDQ3	IMDQ4	IMDQ5	Total population	SII
4CMenB vaccination	165	134	136	144	162	148*	47
Incremental	-39	-49	-48	-46	-42	-44	-14

NA, Not applicable
* Weighted average, given proportions of population by IMDQ

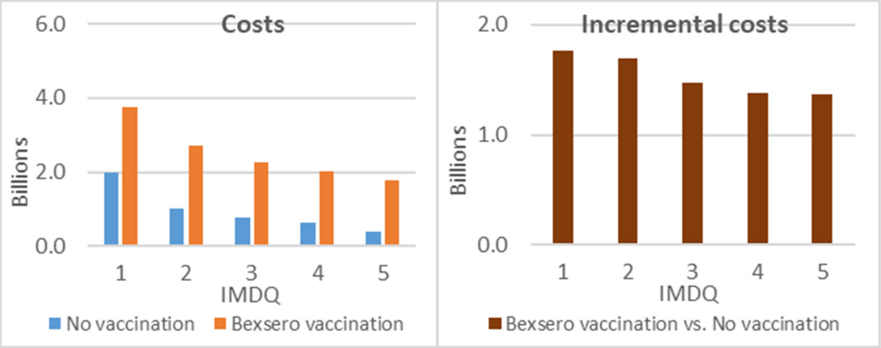

Figure S14. Reference case results - Distribution: Costs (QAF=3 and QAF=1), Payer perspective

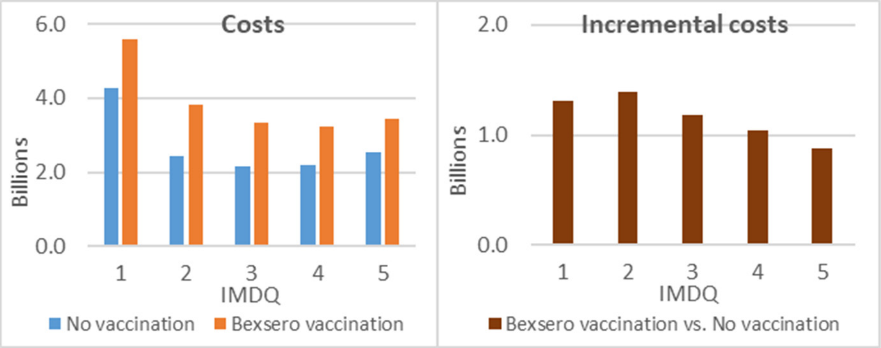

Figure S15. Reference case results - Distribution: Costs (QAF=3 and QAF=1), Societal perspective

7.2. Health equity impact

Inequality index

Net equity impact was calculated for total QALYs following the outline approach in Cookson et al. [31], the results are presented in Table S32 and Table S33. For the baseline reference case with QAF=3 the estimated equity impact was approximately two times higher comparing to the case with QAF=1, which reflected the important impact of the adjustment applied for the quality of life for the disease severity. The absolute measures of inequality (for Kolm-Pollak SWF) were numerically higher, however these should not be directly compared to the relative measures of inequality (from Atkinson SWF), due to the differences in the underlying approaches implied by SWFs.

It was shown that 4CMenB vaccination decreases inequity in a population, with positive net equity impact demonstrated for both reference cases, and for both estimated measures of inequality.

Equity-efficiency impact planes are presented in Figure S16 and Figure S17, for QAF=3 and QAF=1 respectively. 4CMenB vaccination could be located in the “win-win” quadrant for QAF=3 and societal perspective (i.e., cost-effective and improving equity); or “lose-win” quadrant for QAF=3 in payer perspective, and for QAF=1 in both perspectives (i.e., not cost-effective and improving equity).

Table S32. Reference case (baseline) results - Equity impact: Inequality index (QAF = 3).

	Atkinson SWF	Kolm-Pollak SWF
EDEH: No vaccination	24.05	24.21
EDEH: 4CMenB vaccination	24.05	24.21
Inequality index: No vaccination	0.00974	0.07529
Inequality index: 4CMenB vaccination	0.00972	0.07515
**Net equity impact**	**0.00002 (2.0E-0.5)**	**0.00014 (1.4E-0.5))**
**NHB, Payer perspective**	**-22,325**	
**NHB, Societal perspective**	**71,727**	

Values in brackets provide net equity impact in scientific number format.

Table S33. Reference case results - Equity impact: Inequality index (QAF = 1).

	Atkinson SWF	Kolm-Pollak SWF
EDEH No vaccination	24.05	24.21
EDEH 4CMenB vaccination	24.05	24.21
Inequality index No vaccination	0.00970	0.07502
Inequality index 4CMenB vaccination	0.00969	0.07495
**Net equity impact**	**0.00001 (1.1E-05)**	**0.00007 (7.3E-0.5)**
**NHB, Payer perspective**	**-190,215**	
**NHB, Societal perspective**	**-96,163**	

Values in brackets provide net equity impact in scientific number format.

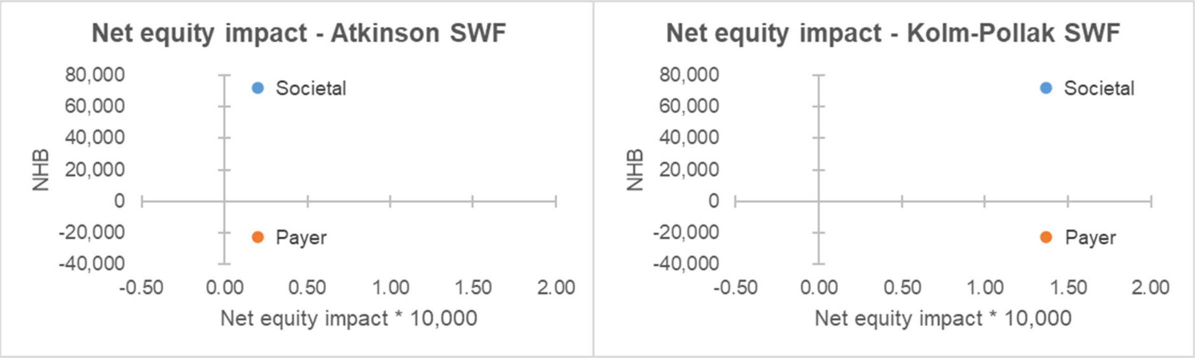

Figure S16. Reference case (baseline) results – Equity-efficiency impact plane (QAF=3)

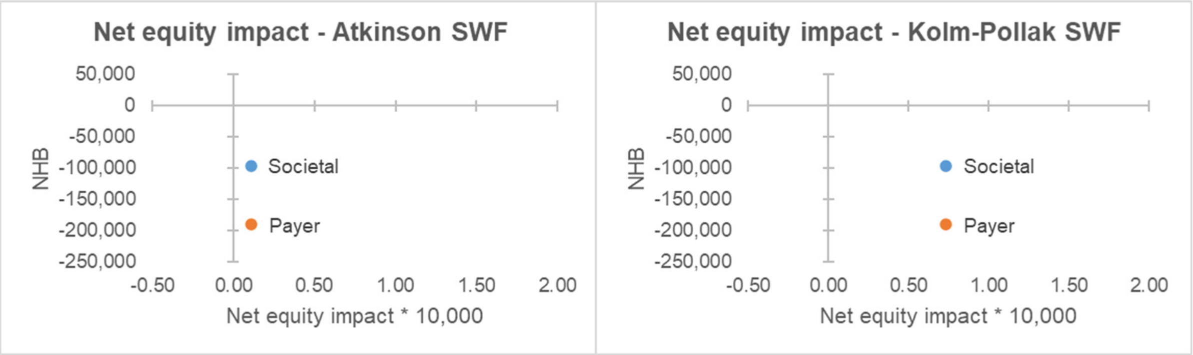

Figure S17. Reference case results - Equity-efficiency impact plane (QAF=1)

7.3. Full DCEA

CEA summary

The summary of aggregated costs and QALYs and cost-effectiveness estimates are provided in Table S34.

For the baseline reference case with QAF=3, ICER was lower comparing to the reference case with QAF=1, estimated at £16,035 and £29,919 per QALY gained respectively, for a societal perspective. In the original analysis the ICERs were equal to £14,034 and £26,247 per QALY gained for QAF=3 and QAF=1 respectively. The difference between an original and adapted analyses is related mostly to the stratification of the life expectancy, utility and annual income. The similar trend was observed for a payer perspective: the adapted model estimated ICERs at £21,234 and £39,621 per QALY gained, and the original model estimated those at £20,876 and £39,045 per QALY gained, for QAF=3 and QAF=1 respectively.

Table S34. Reference case (baseline) results - Full DCEA: CEA summary (QAF = 3).

	QAF=3	QAF=1
Incremental QALYs	361,779	193,888
**Payer perspective**		
Incremental costs	7,682,074,661	
ICER (unweighted)	**21,234**	**39,621**
NHB (unweighted)	-22,325	-190,215
**Societal perspective**		
Incremental costs	5,801,034,391	
**ICER (unweighted)**	**16,035**	**29,919**

	QAF=3	QAF=1
NHB (unweighted)	71,727	-96,163

Threshold weighting (WTP)

The obtained results suggest that 4CMenB vaccination decreased inequality in the population. The exploratory analysis showed, that WTP weights in the range of 2.5-15 would imply cost-effectiveness of the 4CMenB vaccination vs. no vaccination, regardless of QAF and perspective considered. Detailed data are shown in Table S35 and

Table S36.

Table S35. Reference case (baseline) results - Threshold WTP weighting (QAF = 3).

	WTP weight=2.5	WTP weight =5	WTP weight =10	WTP weight=15
Weighted WTP (£/QALY)	50,000	100,000	200,000	300,000
**Payer perspective**				
NHB (unweighted WTP)	-22,325			
NHB (weighted WTP)	208,137	284,958	323,369	336,172
**Societal perspective**				
NHB (unweighted WTP)	71,727			
NHB (weighted WTP)	245,758	303,769	332,774	342,442

Table S36. Reference case results - Threshold WTP weighting (QAF = 1).

	WTP weight=2.5	WTP weight =5	WTP weight =10	WTP weight=15
Weighted WTP (£/QALY)	50,000	100,000	200,000	300,000
**Payer perspective**				
NHB (unweighted WTP)	-190,215			
NHB (weighted WTP)	40,247	117,068	155,478	168,282
**Societal perspective**				
NHB (unweighted WTP)	-96,163			
NHB (weighted WTP)	77,868	135,878	164,883	174,552

QALY weighting (Total QALY)

The alternative approach incorporating equity into CEA framework allows to weight the health outcomes (e.g., total QALYs) according to their distribution among socioeconomic groups. It was shown, that ICER decreases considerably, across reference cases, perspectives and SWFs applied, which is in line with its positive equity impact. It should be noted that QALY weights are slightly lower for analyses with QAF=1 compared to analyses with QAF=3 (for Atkinson and Kolm-Pollak SWFs), as each QALY is "valued" higher when the quality adjustment is applied.

The estimated weighted ICERs were below the WTP threshold of £20,000 per QALY gained for all explored settings, apart from the reference case with QAF=1 when the weighting with Kolm-Pollak SWF was applied (for payer and societal perspectives). The detailed results are provided in Table S37, Table S38, Table S39 and Table S40.

Table S37. Reference case (baseline) results - QALY weighting, Total QALY: Atkinson SWF (QAF = 3).

Atkinson SWF	IMDQ1	IMDQ2	IMDQ3	IMDQ4	IMDQ5	Total population
**No vaccination**						
QALYs (unweighted)	770,654,617	694,825,898	606,837,931	560,812,046	543,384,559	3,176,515,051
**QALY weights**	**3.566**	**1.810**	**1.454**	**1.293**	**1.000**	**NA**
QALYs (weighted)	2,748,114,384	1,257,963,961	882,170,071	725,379,899	543,384,559	6,157,012,874
**4CMenB vaccination**						
QALYs (unweighted)	770,794,824	694,903,474	606,899,978	560,861,311	543,417,243	3,176,876,830
QALYs (weighted)	2,748,614,355	1,258,104,409	882,260,270	725,443,621	543,417,243	6,157,839,898
**4CMenB vaccination vs No vaccination**						
Incremental QALYs (unweighted)	140,207	77,576	62,047	49,265	32,684	361,779
IncrementalQALYs (weighted)	499,970	140,449	90,199	63,722	32,684	827,024
**Payer perspective**						
NHB (unweighted QALYs)	40,804	-6,667	-10,068	-16,673	-29,721	-22,325
NHB (weighted QALYs)	400,567	56,206	18,084	-2,216	-29,721	442,920
**ICER (weighted QALYs)**						**9,289**
**Societal perspective**						
NHB (unweighted QALYs)	65,144	13,961	7,590	-527	-14,440	71,727
NHB (weighted QALYs)	424,907	76,834	35,742	13,929	-14,440	536,972
**ICER (weighted QALYs)**						**7,014**
Incremental equity benefit						465,245*

NA, Not applicable
*Incremental equity benefit is the same for the payer and societal perspectives, reflecting the difference in weighted vs. unweighted QALYs

Table S38. Reference case results - QALY weighting, Total QALYs: Kolm-Pollak SWF (QAF = 3).

Kolm-Pollak SWF	IMDQ1	IMDQ2	IMDQ3	IMDQ4	IMDQ5	Total population
**No vaccination**						
QALYs (unweighted)	770,654,617	694,825,898	606,837,931	560,812,046	543,384,559	3,176,515,051
**QALY weights**	**1.523**	**1.224**	**1.137**	**1.093**	**1.000**	**NA**
QALYs (weighted)	1,173,389,930	850,665,389	690,266,251	613,053,717	543,384,559	3,870,759,847
**4CMenB vaccination**						
QALYs (unweighted)	770,794,824	694,903,474	606,899,978	560,861,311	543,417,243	3,176,876,830
QALYs (weighted)	1,173,603,408	850,760,363	690,336,829	613,107,572	543,417,243	3,871,225,415
**4CMenB vaccination vs No vaccination**						
Incremental QALYs (unweighted)	140,207	77,576	62,047	49,265	32,684	361,779
Incremental QALYs (weighted)	213,477	94,975	70,577	53,854	32,684	465,568
**Payer perspective**						
NHB (unweighted QALYs)	40,804	-6,667	-10,068	-16,673	-29,721	-22,325

Kolm-Pollak SWF	IMDQ1	IMDQ2	IMDQ3	IMDQ4	IMDQ5	Total population
NHB (weighted QALYs)	114,074	10,732	-1,538	-12,084	-29,721	81,464
**ICER (weighted QALYs)**						**16,500**
**Societal perspective**						
NHB (unweighted QALYs)	65,144	13,961	7,590	-527	-14,440	71,727
NHB (weighted QALYs)	138,414	31,360	16,120	4,062	-14,440	175,516
**ICER (weighted QALYs)**						**12,460**
Incremental equity benefit						103,789*

NA, Not applicable.
*Incremental equity benefit is the same for the payer and societal perspectives, reflecting the difference in weighted vs. unweighted QALYs.

Table S39. Reference case results – QALY weighting, Total QALY: Atkinson SWF (QAF = 1).

Atkinson SWF	IMDQ1	IMDQ2	IMDQ3	IMDQ4	IMDQ5	Total population
**No vaccination**						
QALYs (unweighted)	770,917,208	694,965,233	606,948,336	560,899,712	543,442,061	3,177,172,550
**QALY weights**	**3.557**	**1.809**	**1.453**	**1.293**	**1.000**	**NA**
QALYs (weighted)	2,741,989,973	1,256,912,231	881,595,616	725,092,281	543,442,061	6,149,032,161
**4CmenB vaccination**						
QALYs (unweighted)	770,992,293	695,006,941	606,981,596	560,926,018	543,459,589	3,177,366,438
QALYs (weighted)	2,742,257,034	1,256,987,665	881,643,927	725,126,288	543,459,589	6,149,474,502
**4CmenB vaccination vs No vaccination**						
Incremental QALYs (unweighted)	75,085	41,709	33,260	26,306	17,528	193,888
Incremental QALYs (weighted)	267,061	75,434	48,311	34,007	17,528	442,341
**Payer perspective**						
NHB (unweighted QALYs)	-24,318	-42,534	-38,854	-39,632	-44,877	-190,215
NHB (weighted QALYs)	167,658	-8,809	-23,804	-31,931	-44,877	58,238
**ICER (weighted QALYs)**						**17,367**
**Societal perspective**						
NHB (unweighted QALYs)	22	-21,906	-21,196	-23,486	-29,596	-96,163
NHB (weighted QALYs)	191,998	11,819	-6,146	-15,786	-29,596	152,290
**ICER (weighted QALYs)**						**13,114**
Incremental equity benefit						248,453*

NA, Not applicable
*Incremental equity benefit is the same for the payer and societal perspectives, reflecting the difference in weighted vs. unweighted QALYs.

Table S40. Reference case results - QALY weighting, Total QALY: Kolm-Pollak SWF (QAF = 1).

Kolm-Pollak SWF	IMDQ1	IMDQ2	IMDQ3	IMDQ4	IMDQ5	Total population
**No vaccination**						

QALYs (unweighted)	770,917,208	694,965,233	606,948,336	560,899,712	543,442,061	3,177,172,550
**QALY weights**	**1.521**	**1.224**	**1.137**	**1.093**	**1.000**	**NA**
QALYs (weighted)	1,172,900,759	850,561,509	690,206,519	613,039,352	543,442,061	3,870,150,199
**4CMenB vaccination**						
QALYs (unweighted)	770,992,293	695,006,941	606,981,596	560,926,018	543,459,589	3,177,366,438
QALYs (weighted)	1,173,014,996	850,612,555	690,244,342	613,068,103	543,459,589	3,870,399,586
**4CMenB vaccination vs No vaccination**						
Incremental QALYs (unweighted)	75,085	41,709	33,260	26,306	17,528	193,888
Incremental QALYs (weighted)	114,237	51,047	37,823	28,751	17,528	249,386
**Payer perspective**						
NHB (unweighted QALYs)	-24,318	-42,534	-38,854	-39,632	-44,877	-190,215
NHB (weighted QALYs)	14,834	-33,196	-34,292	-37,187	-44,877	-134,717
**ICER (weighted QALYs)**						**30,804**
**Societal perspective**						
NHB (unweighted QALYs)	22	-21,906	-21,196	-23,486	-29,596	-96,163
NHB (weighted QALYs)	39,174	-12,568	-16,634	-21,041	-29,596	-40,665
**ICER (weighted QALYs)**						**23,261**
Incremental equity benefit						55,498*

NA, Not applicable
*Incremental equity benefit is the same for the payer and societal perspectives, reflecting the difference in weighted vs. unweighted QALYs.

7.4. Sensitivity analysis results

DSA results are presented for: unweighted ICER, weighted ICER for Atkinson and weighted ICER for Kolm-Pollak, for both QAF=3 and QAF=1.

PSA results are presented for: unweighted ICER, weighted ICER for Atkinson and for Kolm-Pollak, equity efficiency impact planes for Atkinson and Kolm-Pollak. The PSA was performed for QAF=3, with 250 simulations.

DSA and PSA results - unweighted ICER

Figure S18 and Figure S19 summarise the results of the DSA for the unweighted ICER in the form of Tornado charts. Key drivers of this analysis were discount rate for outcomes, MenB incidence multiplier, 4CMenB vaccine effectiveness in infants and QAF. The same key drivers were identified in the original model report [35]. None of the equity-related inputs had a significant impact on the results.

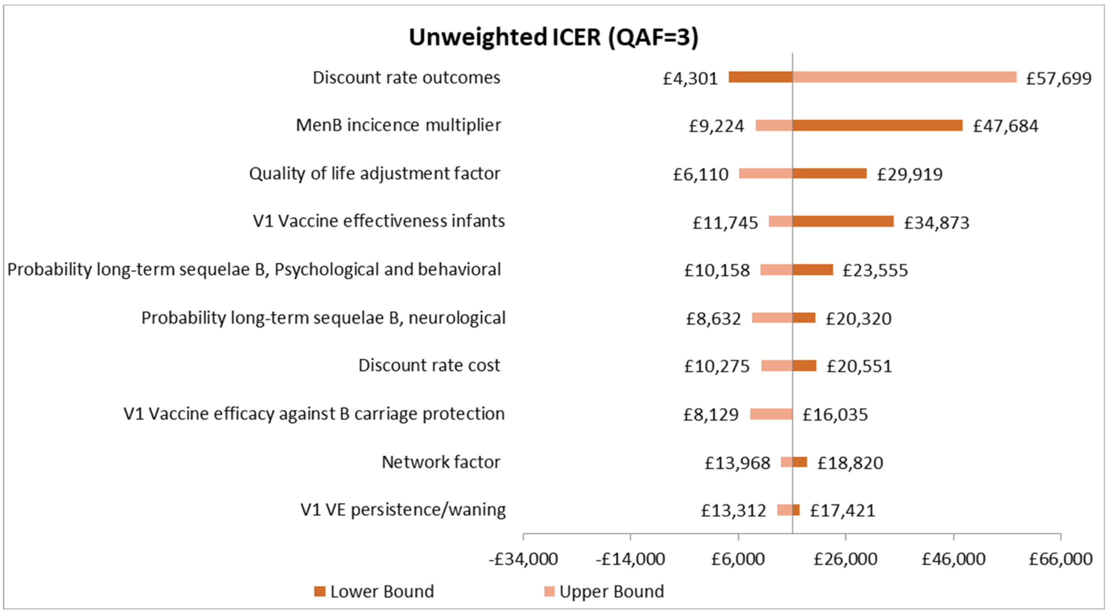

Figure S18. DSA results – Unweighted ICER (QAF = 3); reference case (baseline) unweighted ICER: £16,035 per QALY gained.

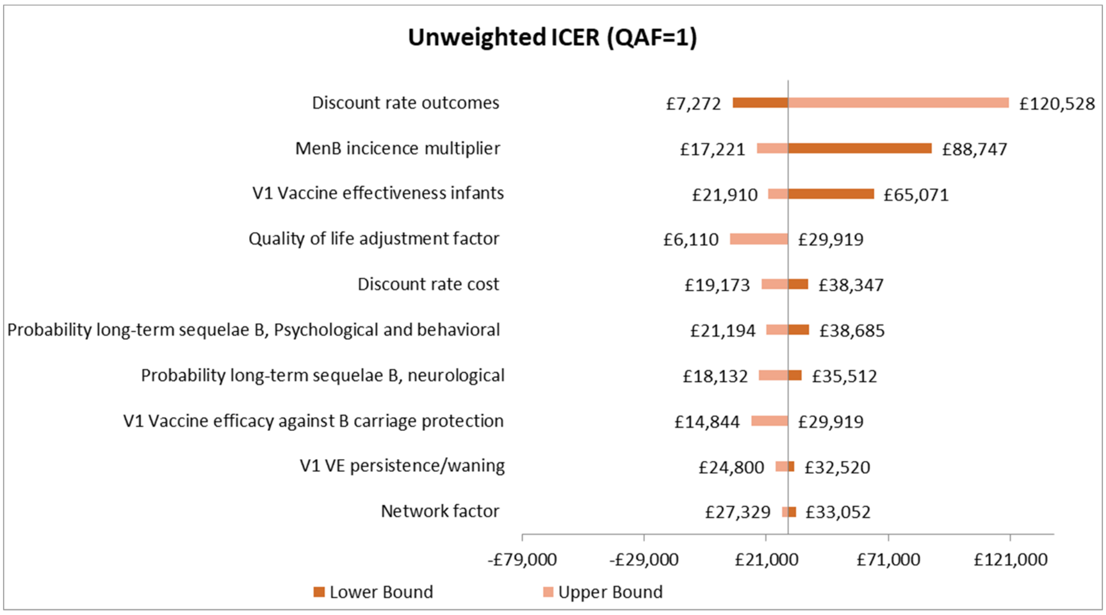

Figure S19. DSA results - Unweighted ICER (QAF = 1); reference case unweighted ICER: £29,919 per QALY gained.

For the unweighted ICER, the incremental cost-effectiveness plane presented on Figure S20 showed that all simulations were in the upper-right quadrant, which indicated that 4CMenB vaccination had a high probability to be more costly but also more effective comparing to no vaccination.

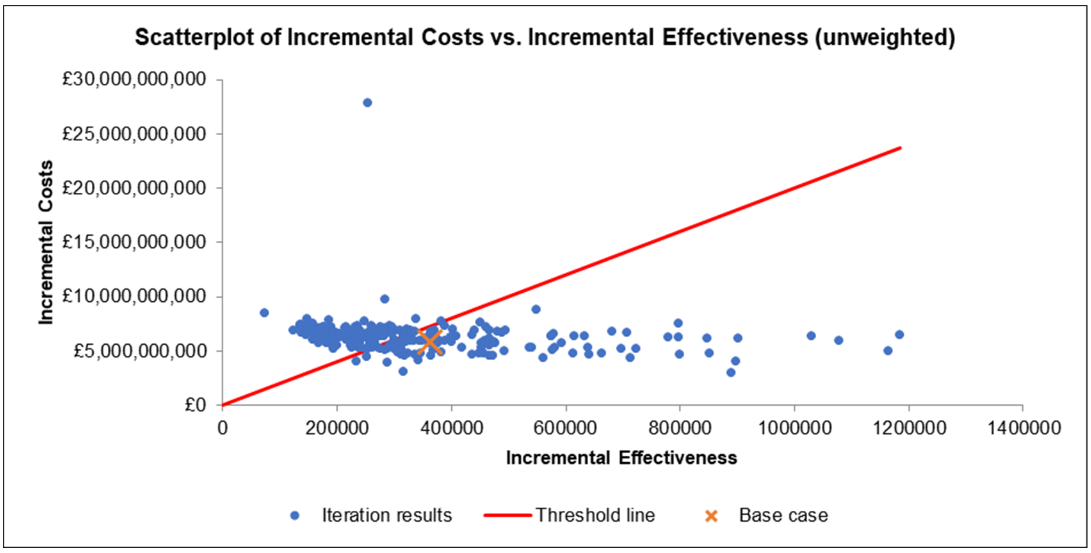

Figure S20. PSA results (QAF = 3)—Scatterplot of Incremental Costs vs. Incremental Effectiveness, unweighted ICER.

DSA and PSA results - Weighted for Atkinson SWF

The Figure S21 and Figure S22 below summarise the results of the DSA for the weighted ICER with the Atkinson SWF in the form of Tornado charts. Key drivers of this analysis were the same as for the unweighted ICER: discount rate for outcomes, MenB incidence multiplier, 4CMenB vaccine effectiveness in infants and QAF. Among equity related inputs, the inequality aversion parameter for the Atkinson SWF had a significant impact on the results.

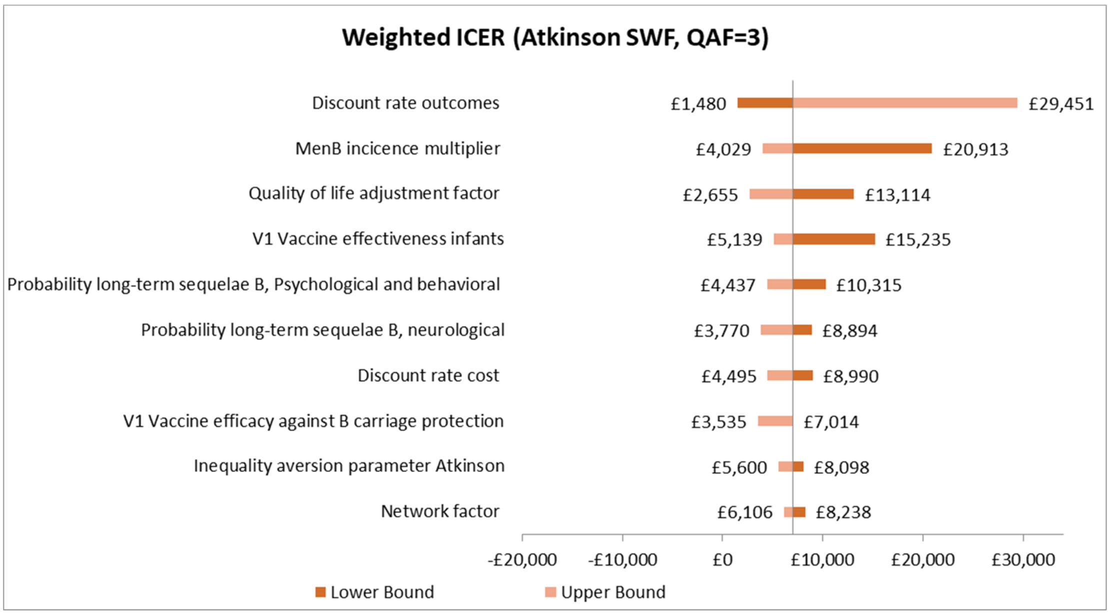

Figure S21. DSA results - Weighted ICER, Atkinson SWF (QAF = 3); reference case (baseline) weighted ICER: £7,014 per QALY gained.

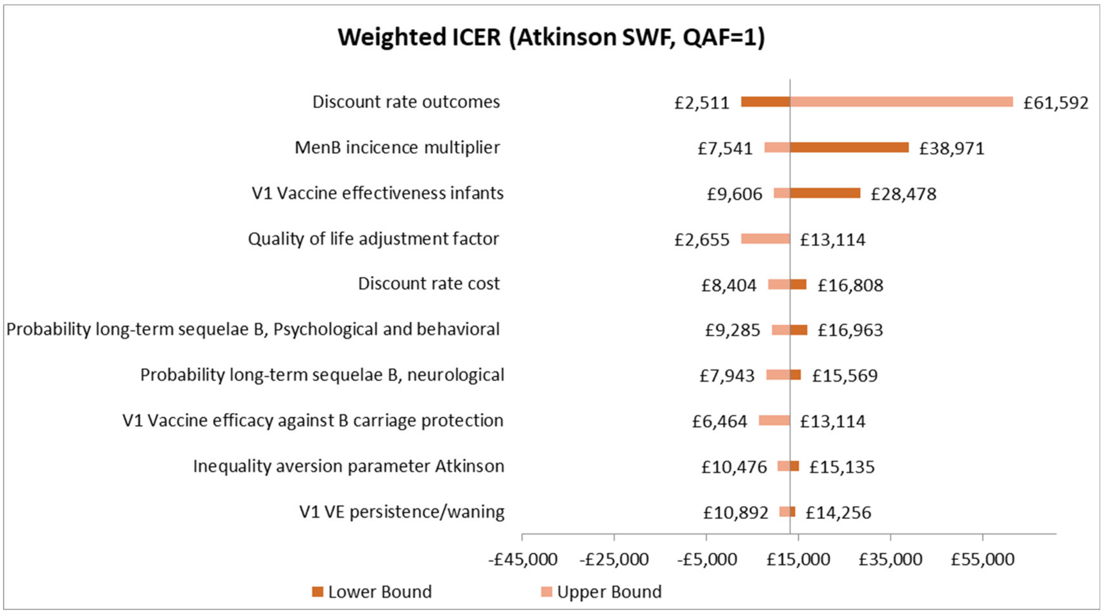

Figure S22. DSA results - Weighted ICER, Atkinson SWF (QAF = 1); reference case weighted ICER: £13,114 per QALY gained.

For the weighted ICER with the Atkinson SWF, the incremental cost-effectiveness plane (Figure S23) showed that all simulations were in the upper-right quadrant, which indicated that 4CMenB vaccination had a high probability to be more costly but also more effective comparing to no vaccination. Almost all simulations were located below the threshold line.

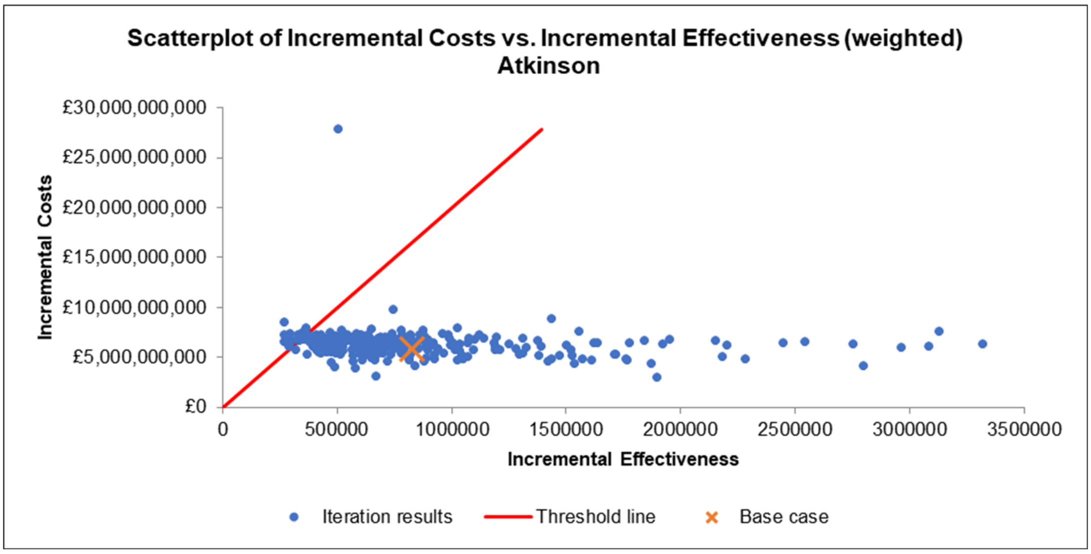

Figure S23. PSA results - Scatterplot of Incremental Costs vs. Incremental Effectiveness, weighted ICER, Atkinson SWF.

Equity impact: Most of the simulations indicated a positive equity impact, while many of them showed that 4CMenB would not be cost-effective (with a negative NHB), as shown on Figure S24.

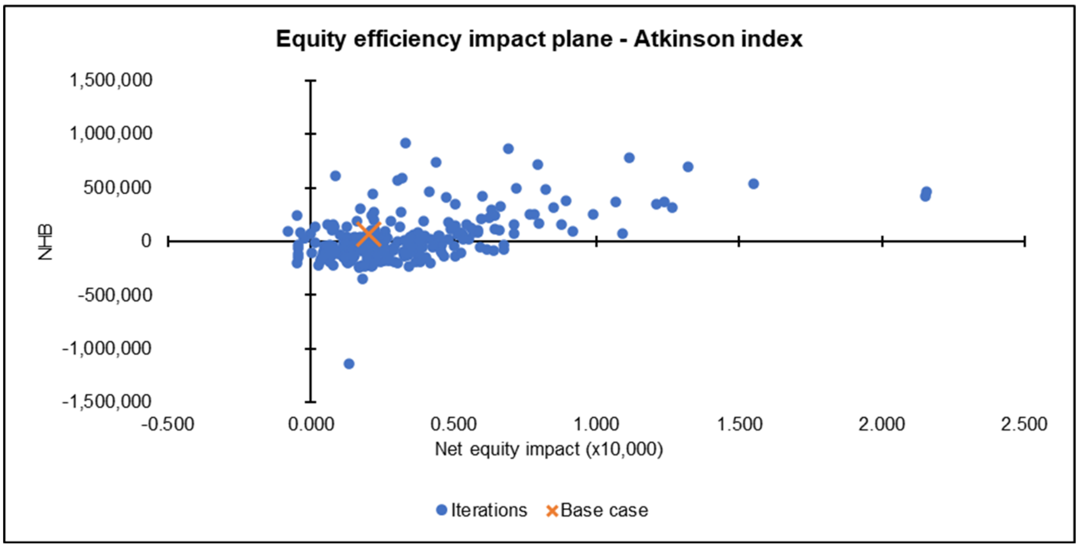

Figure S24. PSA results - Equity efficiency impact plane, Atkinson SWF.

DSA and PSA results - Weighted for Kolm-Pollak SWF

The Figure S25 and Figure S26 below summarise the results of the DSA for the weighted ICER with the Kolm-Pollak SWF in the form of Tornado charts. Key drivers of this analysis were the same as for the unweighted ICER: B incidence multiplier, discount rate for outcomes, 4CMenB effectiveness in infants and quality of life adjustment factor. None of the equity-related inputs had a significant impact on the results.

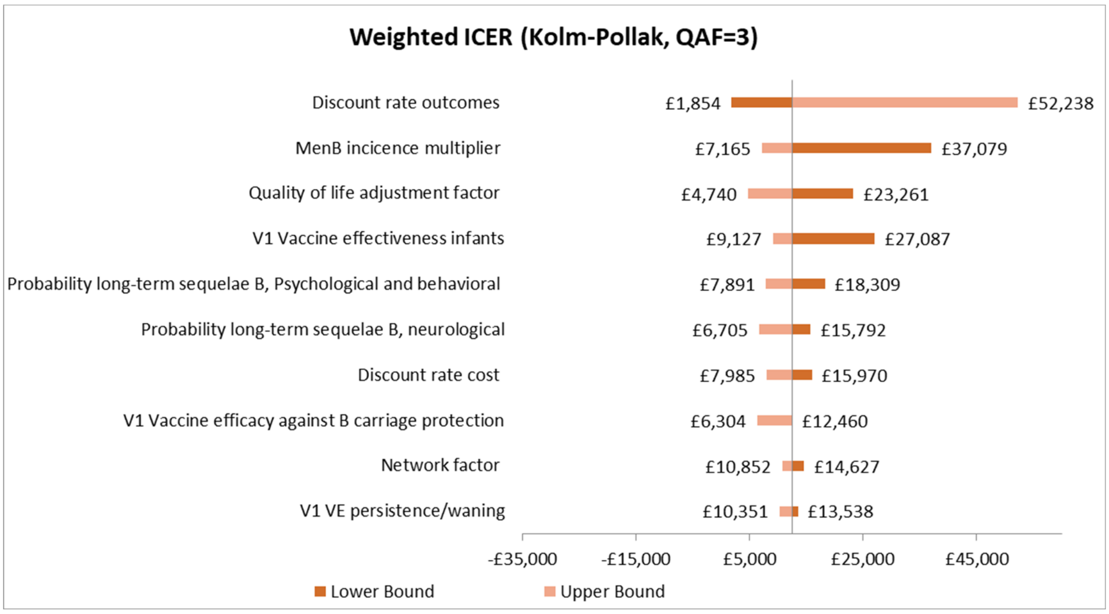

Figure S25. DSA results - Weighted ICER, Kolm-Pollak SWF (QAF = 3); reference case (baseline) weighted ICER: £12,460/QALY gained.

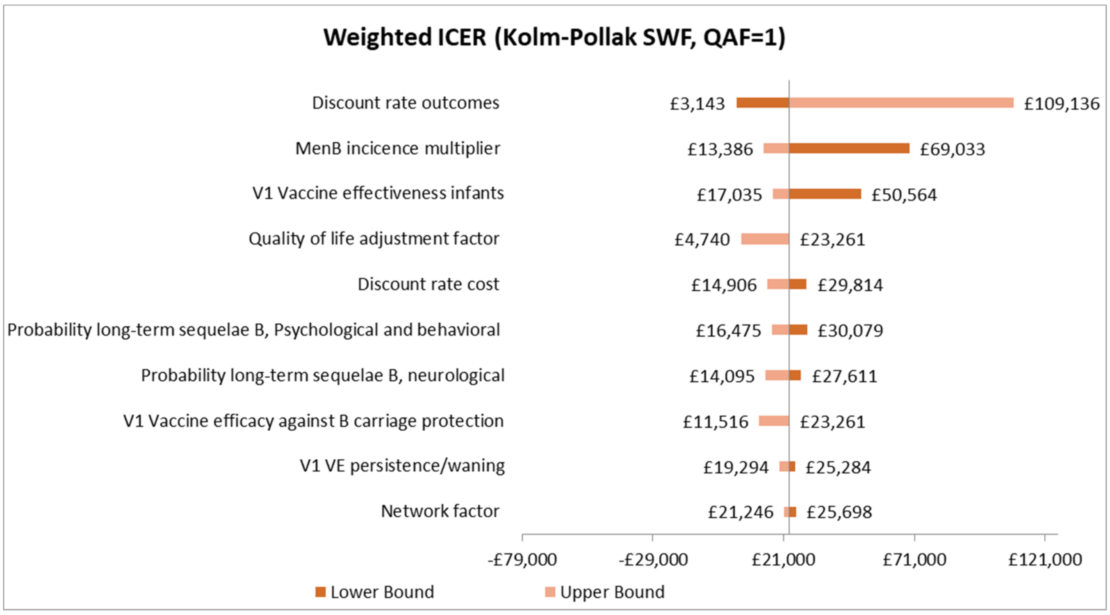

Figure S26. DSA results - Weighted ICER, Kolm-Pollak SWF (QAF = 1); reference case weighted ICER: £23,261/QALY gained.

For the weighted ICER with the Kolm-Pollak SWF, the incremental cost-effectiveness plane (Figure S27) showed that all simulations were in the upper-right quadrant, which indicated

that 4CMenB vaccination had a high probability to be more costly but also more effective comparing to no vaccination.

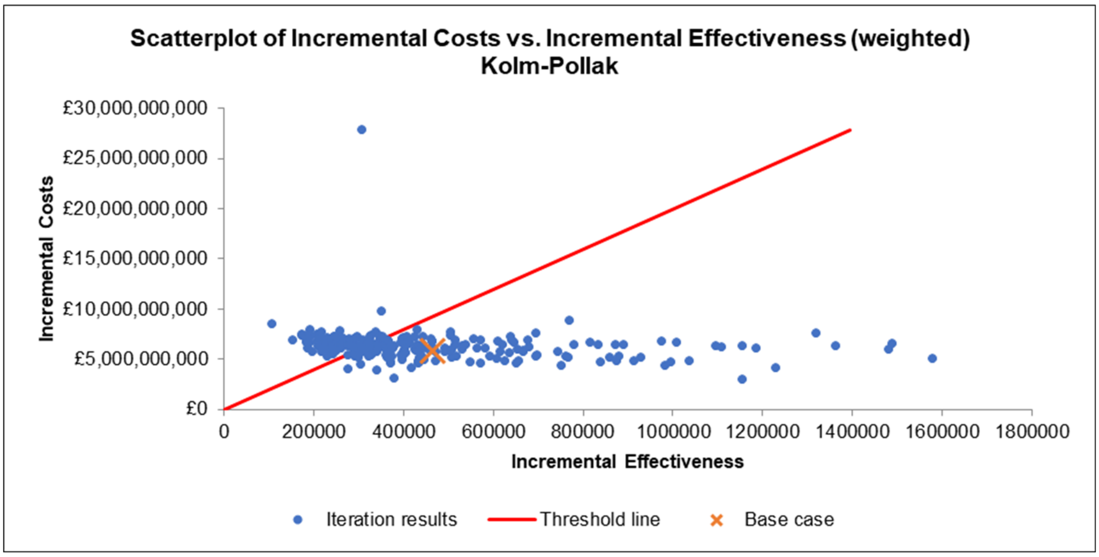

Figure S27. PSA results - Scatterplot of Incremental Costs vs. Incremental Effectiveness, weighted ICER, Kolm-Pollak SWF.

Equity impact: most of the simulations indicated a positive equity impact, while many simulations showed that 4CMenB would not be cost-effective (with a negative NHB), as shown on Figure S28.

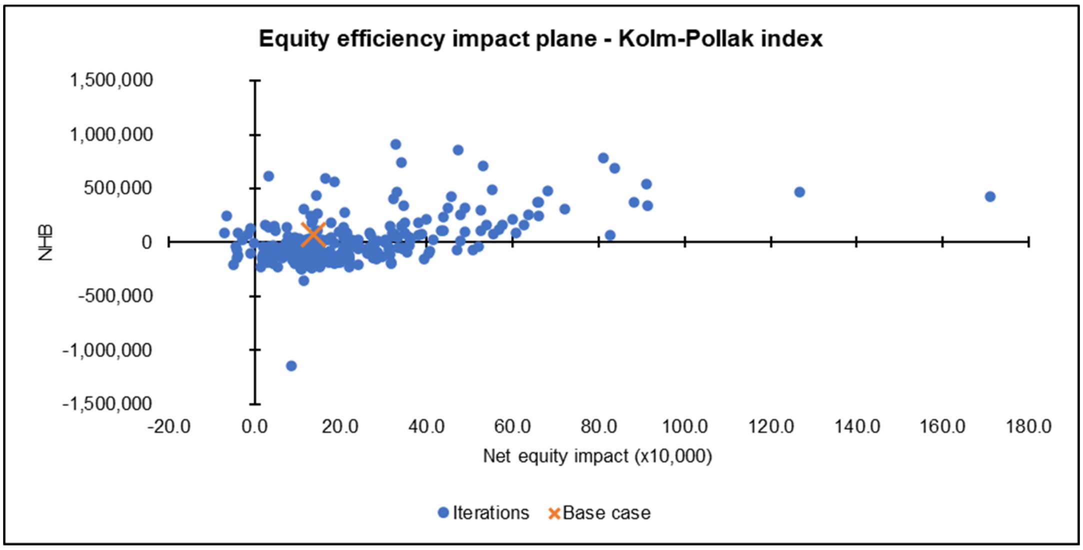

Figure S28. PSA results - Equity efficiency impact plane, Kolm-Pollak SWF.

8. Discussion

Methods: stratification

- For this pilot study, the DTM was not modified. The stratification was applied to the outputs of the DTM (cases of invasive meningococcal disease) and impacted only the cost effectiveness decision tree. Thus, all strata were analysed separately, and *N. meningitidis* transmission between strata was not taken into account. This assumption is likely conservative, as it underestimates vaccination benefits for eradicating pathogens within strata with higher carriage, thus reducing the risk of dissemination and epidemics in the general population. Whereas equity should be considered in the design of any new model from the beginning, in case of 4CMenB infant vaccination this 'adaptation' approach can be justified as no robust evidence on the impact of 4CMenB vaccination on transmission of meningococcal carriage is available to date.
- The analysis considered stratification of the target population by socioeconomic status, using IMDQ. This index does not reflect the characteristics of an individual, however, it captures social deprivation at the level of small areas, incorporating geographical differences within the general population. Stratification into five subgroups in future adaptations could be applied to other strata such as race/ethnicity or other health equity strata.
- Stratification was applied to the number of cases using a specific estimated case proportion for each stratum. These proportions were estimated using the carriage prevalence, strata size and average vaccine effective coverage. The average vaccine effective coverage was derived in a simplified way, assuming that each subsequent dose of the vaccine equals to or supersedes effectiveness of the previous one.
- The stratification considers only direct protection and direct cross-protection against invasive meningococcal disease, as the carriage effect (and potential herd immunity) was not included in the original 4CMenB analysis. Also, the calculated estimated case proportion assumes the same risk of developing disease in carriers, regardless of socioeconomic factors. Though applied in an adapted fashion, this approach follows in-principle the approach of the DTM of the underlying model to estimate invasive meningococcal disease cases given acquisition of meningococcal carriage.
- The presented approach, applying the estimated case proportion, should be considered as a proxy, developed for the preliminary assessment prior to the comprehensive DCEA. Particularly, stratum-specific epidemiologic and vaccination-related inputs, contacts between individuals from the different strata, movement of individuals between strata over time, herd effect and protection against carriage transmission could be captured in future adaptations, which would require modification of the DTM. However, comparing the ICERs of the adapted, equity-stratified approach with the ICERs of the original underlying model for both QAF=3 and QAF=1, the ICERs were very similar suggesting that the equity stratified approach for estimating MenB cases per strata is a reasonable approximation.

Methods: equity impact

- The further implementation of the DCEA framework into the formal HTA process could include the definition of criteria for the interpretation of equity measures, e.g., the standardization of the net equity impact threshold to apply WTP weighting, similar to the criteria for end-of-life treatment for which a modified threshold should be applied. This would also facilitate the understanding and interpretation of net equity impact outcomes, as current estimates are challenging to interpret, besides inferring whether the net equity impact is significant or not.

- The exploratory analyses have shown that Atkinson SWF is not applicable for negative outcomes, like QALY loss, and certain adaptation could be needed for approaches based on Kolm-Pollak SWF. In health economic modelling for vaccines, traditionally QALY loss per vaccination strategy/arm is computed rather than total QALYs, as such the underlying model estimated QALY loss. Thus, the requirement of the current DCEA methodology had to be addressed in the present adaptation by an estimation of the total QALYs in population.
- For some health outcomes which are routinely used in CEA (e.g., number of cases, deaths, etc.) a dedicated framework for interpretation should be developed. For instance, the definition of a threshold to compute NHB or another applicable measure aggregating costs and outcomes, needed for the full DCEA, would be required.
- It is not clear whether inequity measures computed for different populations or disease areas are comparable, which could be a potential obstacle for decision-makers when there is a need to allocate limited resources and choose between several interventions with positive equity impact. Within this context it may be helpful to further specify criteria and interpretation supporting decision making in different situations, particularly, whether to apply as a reference case either a relative inequality index (e.g., Atkinson) or an absolute one (e.g., Kolm-Pollak).
- The implications of the population-level and cohort-level models, and the time horizon considered are yet to be defined, as the modelling framework applied defines the target health outcome (e.g., average total QALYs in population over time horizon vs. total QALYs over an individual's lifetime).
- This study focused on the health-related benefits. Explicit modelling of the distribution of financial benefits of vaccination, including the statistical relationship between health outcomes and health expenditures, as well as out-of-pocket payments, financial risk protection and other monetary measures could be considered for further analyses. For instance, it should be noted that invasive meningococcal disease is a devastating condition which could lead to catastrophic consequences, thus financial risk protection (including the risk of falling into poverty), could be an important element of vaccination value. By contrast, out-of-pocket payments could be of lower significance, as the UK setting applies universal healthcare, i.e., healthcare services and vaccination are provided for free (no copay required). The uptake of non-NIP vaccination is very low in the UK, suggesting that private payments for vaccination in a studied population are not common.
- This study assumed that the health opportunity cost (payer and societal perspectives) is distributed evenly across strata, i.e., the same threshold and cost for each individual within each IMDQ was applied, which was in line with expert insights from the latest unpublished research and previous DCEA [32,33]. For direct and indirect costs, an assumption on the equal distribution of the health opportunity cost across IMDQ was applied. For direct costs (i.e., payer perspective), this approach seems realistic, although it is not evidence-based [33] and implies the existing equality in access to healthcare, as well as reallocation of vaccination budget elsewhere in healthcare. The latter could be challenged, as vaccination budgets are often separate from healthcare budgets for treatments; therefore, additional expenditures or savings related to vaccination could be not easily transferrable to other sectors in healthcare.
- For the societal perspective, further studies would be needed to define the framework allowing to consider uneven distribution of the income and lost productivity across strata, as well as potential benefits stemming from savings in indirect cost achieved

elsewhere in a population (presumably, also including the value related to financial risk protection and other monetary outcomes). It is important to consider comparability of the framework for the assessment of vaccination and non-vaccination interventions

- This study did not include a sensitivity analysis for the distribution of health opportunity costs, and it could be advantageous to include it in further analyses.

Inputs and results

- This analysis aimed to stratify an existing CEA of 4CMenB, keeping the analysis settings as close as possible to the original ones, while providing a plausible stratification. Thus, all inputs were standardized to the original model whenever possible. Such standardization appears justifiable for the illustrative case study, however it could lead to a certain bias. Ideally, DCEA should be planned early in the process of modelling, with the aim to facilitate evidence generation, providing comprehensive data on the drivers of inequity in the population studied, and capture the relevant disease and societal processes with the applied modelling approach.
- In the context of this study, concern was raised for stratification of life expectancy and utility given the multiplicative approach to estimate QALY losses in MenB cases, as it could result in disproportionally higher disutility for some strata, especially with the multiplicative approach for the estimation of disutilities. This could be not in line with the general equity concept and could contribute to biased results. However, it could also be argued that a disutility due to a certain health condition is not necessarily the same for individuals with different baseline utilities. It has been previously shown that the perceived reduction of quality of life varies in patients from different socioeconomic groups [55,56]. Thus, a comprehensive DCEA should be informed by dedicated research, capturing the association between socioeconomic status, severity of disease, perceived quality of life, valuation of different outcomes and other relevant dimensions for decision-makers. Additionally, the optimal methodology for the estimation of QALY loss is yet to be defined (e.g., multiplicative vs. additive approach). For this pilot study, no significant bias was expected due to the applied multiplicative approach and stratification of life expectancy and utility, as the reference case results were reasonably close to the results of dedicated analyses which did not consider stratification for one or both groups of the parameters.
- The estimated net equity impact measures suggest that 4CMenB vaccination improves equity in the population, although the magnitude of this outcome appears to be relatively small, which may be due to the low incidence of the invasive meningococcal disease. However, no threshold exists yet to judge the significance of the equity impact.
- Inclusion of modifiers accounting for severity (e.g., QAF) and equity (e.g., inequality aversion parameter) in the same analysis could be criticized, as it could lead to the overestimation of the equity impact. However, to the best of our knowledge at present, we are not aware of any published conclusive data or studies evaluating societal preferences for prevention of severe disease versus preventing disease in the most deprived, or a 'joint' preference for the prevention of severe disease in the most deprived. The comparison of the explored reference cases with QAF=3 and QAF=1 showed that this modifier has an important impact on the DCEA results.
- This model adaptation considered discounting for the health and cost outcomes for the reference case, in line with the standard CEA methodology and previous 4CMenB analysis [29,47] , while discounting is not used routinely in analyses on health equity [31,33]. The DSA, showed that discounting parameter is one of the key drivers of the

model results, thus a consensus on the appropriate use of discounting in DCEA has to be reached.

- This study suggested that 4CMenB vaccination has a positive equity impact compared to no vaccination in both reference cases, whereas the conclusion on its cost-effectiveness is sensitive to the considered QAF and the analysis perspective (i.e., payer vs. societal).

9. References

1. Martin, A.; Batty, A.; Roberts, J.A.; Standaert, B. Cost-effectiveness of infant vaccination with RIX4414 (Rotarix) in the UK. *Vaccine* **2009**, *27*, 4520-4528, doi:https://doi.org/10.1016/j.vaccine.2009.05.006.
2. Sandmann, F.G.; Robotham, J.V.; Deeny, S.R.; Edmunds, W.J.; Jit, M. Estimating the opportunity costs of bed-days. *Health economics* **2018**, *27*, 592-605, doi:https://doi.org/10.1002/hec.3613.
3. Sandmann, F.G.; Shallcross, L.; Adams, N.; Allen, D.J.; Coen, P.G.; Jeanes, A.; Kozlakidis, Z.; Larkin, L.; Wurie, F.; Robotham, J.V.; et al. Estimating the hospital burden of norovirus-associated gastroenteritis in England and its opportunity costs for nonadmitted patients. *Clinical infectious diseases : an official publication of the Infectious Diseases Society of America* **2018**, *67*, 693-700, doi:https://doi.org/10.1093/cid/ciy167.
4. Velázquez, F.R.; Matson, D.O.; Calva, J.J.; Guerrero, L.; Morrow, A.L.; Carter-Campbell, S.; Glass, R.I.; Estes, M.K.; Pickering, L.K.; Ruiz-Palacios, G.M. Rotavirus infection in infants as protection against subsequent infections. *The New England journal of medicine* **1996**, *335*, 1022-1028, doi:https://doi.org/10.1056/nejm199610033351404.
5. UK government. Rotavirus immunisation programme for infants. UK Health Security Agency. Available online: https://www.gov.uk/government/collections/rotavirus-vaccination-progarmme-for-infants (accessed on 09-03-2023).
6. Hungerford, D.; Read, J.M.; Cooke, R.P.; Vivancos, R.; Iturriza-Gómara, M.; Allen, D.J.; French, N.; Cunliffe, N. Early impact of rotavirus vaccination in a large paediatric hospital in the UK. *The Journal of hospital infection* **2016**, *93*, 117-120, doi:https://doi.org/10.1016/j.jhin.2015.12.010.
7. Heinsbroek, E.; Hungerford, D.; Cooke, R.P.D.; Chowdhury, M.; Cargill, J.S.; Bar-Zeev, N.; French, N.; Theodorou, E.; Standaert, B.; Cunliffe, N.A. Do hospital pressures change following rotavirus vaccine introduction? A retrospective database analysis in a large paediatric hospital in the UK. *BMJ open* **2019**, *9*, e027739, doi:https://doi.org/10.1136/bmjopen-2018-027739.
8. Keeble, E.; Kossarova, L.; Foundation., T.H.; Trust., N. Focus on: Emergency hospital care for children and young people. 2017. Available online: https://www.nuffieldtrust.org.uk/research/focus-on-emergency-hospital-care-for-children-and-young-people (accessed on 07-02-2023).
9. Scobie, S. Snowed under: understanding the effects of winter on the NHS. Available online: https://www.nuffieldtrust.org.uk/resource/snowed-under-understanding-the-effects-of-winter-on-the-nhs (accessed on 01.08.2022).

10. NHS England. Consultant-led referral to treatment waiting times data 2018-19. Available online: https://www.england.nhs.uk/statistics/statistical-work-areas/rtt-waiting-times/rtt-data-2018-19/ (accessed on 06-02-2023).

11. Royal College of Paediatrics and Child Health. National guidance for the restoration and recovery of elective surgery in children. Available online: https://www.rcpch.ac.uk/resources/national-guidance-restoration-recovery-elective-surgery-children (accessed on 06-02-2023).

12. Royal College of Paediatrics and Child Health. Recovery of elective surgery in children - Nov 2021 guidance, appendix and posters. Available online: https://www.rcpch.ac.uk/resources/national-guidance-restoration-recovery-elective-surgery-children (accessed on 09-03-2023).

13. Thomas, S.L.; Walker, J.L.; Fenty, J.; Atkins, K.E.; Elliot, A.J.; Hughes, H.E.; Stowe, J.; Ladhani, S.; Andrews, N.J. Impact of the national rotavirus vaccination programme on acute gastroenteritis in England and associated costs averted. *Vaccine* **2017**, *35*, 680-686, doi:https://doi.org/10.1016/j.vaccine.2016.11.057.

14. UK NHS. Rotavirus immunisation programme for infants. UK Health Security Agency. Service specification No.5 Available online: https://assets.publishing.service.gov.uk/government/uploads/system/uploads/attachment_data/file/386238/No05_Rotavirus_Immunisation.pdf (accessed on 01-02-2022).

15. Hungerford, D.; Vivancos, R.; Read, J.M.; Pitzer, V.E.; Cunliffe, N.; French, N.; Iturriza-Gómara, M. In-season and out-of-season variation of rotavirus genotype distribution and age of infection across 12 European countries before the introduction of routine vaccination, 2007/08 to 2012/13. *Euro surveillance : bulletin Europeen sur les maladies transmissibles = European communicable disease bulletin* **2016**, *21*, doi:https://doi.org/10.2807/1560-7917.es.2016.21.2.30106.

16. Public Health England. Laboratory reports of rotavirus infections in England and Wales, by month of report. Available online: https://www.gov.uk/government/publications/rotavirus-laboratory-confirmed-cases-of-rotavirus-infections-in-england-and-wales/rotavirus-laboratory-confirmed-cases-of-rotavirus-infections-in-england-and-wales-2000-to-2013 (accessed on 09-03-2023).

17. Paediatric Intensive Care Audit Network (PICANet). Annual Report 2019. Available online: https://www.picanet.org.uk/wp-content/uploads/sites/25/2019/12/PICANet-2019-Annual-Report-Summary_v1.0.pdf (accessed on

18. UK Department of Health. Implementation of modified admission MRSA screening guidance for NHS (2014). Available online: https://assets.publishing.service.gov.uk/government/uploads/system/uploads/attachment_data/file/345144/Implementation_of_modified_admission_MRSA_screening_guidance_for_NHS.pdf (accessed on 09-03-2023).

19. Dixon, P.; Hollingworth, W.; Benger, J.; Calvert, J.; Chalder, M.; King, A.; MacNeill, S.; Morton, K.; Sanderson, E.; Purdy, S. Observational Cost-Effectiveness Analysis Using Routine Data: Admission and Discharge Care Bundles for Patients with Chronic Obstructive Pulmonary Disease. *PharmacoEconomics - Open* **2020**, *4*, 657-667, doi:https://doi.org/10.1007/s41669-020-00207-w.

20. Robotham, J.V.; Deeny, S.R.; Fuller, C.; Hopkins, S.; Cookson, B.; Stone, S. Cost-effectiveness of national mandatory screening of all admissions to English National Health Service hospitals for meticillin-resistant Staphylococcus aureus: a

mathematical modelling study. *The Lancet Infectious Diseases* **2016**, *16*, 348-356, doi:https://doi.org/10.1016/S1473-3099(15)00417-X.

21. NHS England. Consultant-led referral to treatment waiting times data 2021-22. Available online: https://www.england.nhs.uk/statistics/statistical-work-areas/rtt-waiting-times/rtt-data-2021-22/ (accessed on 06-02-2023).

22. Suhonen, R.; Virtanen, H.; Heikkinen, K.; Johansson, K.; Kaljonen, A.; Leppänen, T.; Salanterä, S.; Leino-Kilpi, H. Health-related quality of life of day-case surgery patients: a pre/posttest survey using the EuroQoL-5D. *Quality of Life Research* **2008**, *17*, 169-177, doi:https://doi.org/10.1007/s11136-007-9292-3.

23. Kwok, C.S.; Gordon, A.C. General paediatric surgery for patients aged under 5 years: a 5-year experience at a district general hospital. *Annals of the Royal College of Surgeons of England* **2016**, *98*, 479-482, doi:https://doi.org/10.1308/rcsann.2016.0175.

24. Gov.UK. NHS reference costs 2009-2010. Available online: https://www.gov.uk/government/publications/nhs-reference-costs-2009-2010 (accessed on 06-02-2023).

25. Tanner, H.; Boxall, E.; Osman, H. Respiratory viral infections during the 2009-2010 winter season in Central England, UK: incidence and patterns of multiple virus co-infections. *Eur J Clin Microbiol Infect Dis* **2012**, *31*, 3001-3006, doi:https://doi.org/10.1007/s10096-012-1653-3.

26. Cromer, D.; van Hoek, A.J.; Jit, M.; Edmunds, W.J.; Fleming, D.; Miller, E. The burden of influenza in England by age and clinical risk group: A statistical analysis to inform vaccine policy. *Journal of Infection* **2014**, *68*, 363-371, doi:https://doi.org/10.1016/j.jinf.2013.11.013.

27. UK NHS. Hospital Admitted Patient Care Activity, 2015-16: Healthcare Resource Groups. Available online: https://digital.nhs.uk/data-and-information/publications/statistical/hospital-admitted-patient-care-activity/2015-16#:~:text=16.3%20million%20Finished%20Admission%20Episodes,to%2049%20in%202005%2D06. (accessed on 13-07-2022).

28. National Institute for Health and Clinical Excellence (NICE). *Guidelines, in Respiratory Tract Infections - Antibiotic Prescribing: Prescribing of Antibiotics for Self-Limiting Respiratory Tract Infections in Adults and Children in Primary Care.*; 2008.

29. Beck, E.; Klint, J.; Neine, M.; Garcia, S.; Meszaros, K. Cost-Effectiveness of 4CMenB infant vaccination in england: A comprehensive valuation considering the broad impact of serogroup b invasive meningococcal disease. *Value Health* **2021**, *24*, 91-104, doi:https://doi.org/10.1016/j.jval.2020.09.004.

30. Taha, M.-K.; Martinon-Torres, F.; Köllges, R.; Bonanni, P.; Safadi, M.A.P.; Booy, R.; Smith, V.; Garcia, S.; Bekkat-Berkani, R.; Abitbol, V. Equity in vaccination policies to overcome social deprivation as a risk factor for invasive meningococcal disease. *Expert review of vaccines* **2022**, *21*, 659-674, doi:https://doi.org/10.1080/14760584.2022.2052048.

31. Cookson, R.; Griffin, S.; Culyer, A.J.; Norheim, O.F. *Distributional Cost-Effectiveness Analysis: Quantifying Health Equity Impacts and Trade-Offs*; Oxford University Press: 2020.

32. Cookson, R.; Mirelman, A.J.; Griffin, S.; Asaria, M.; Dawkins, B.; Norheim, O.F.; Verguet, S.; A, J.C. Using cost-effectiveness analysis to address health equity concerns. *Value Health* **2017**, *20*, 206-212, doi:https://doi.org/10.1016/j.jval.2016.11.027.

33. Asaria, M.; Griffin, S.; Cookson, R. Distributional Cost-Effectiveness Analysis: A Tutorial. *Med Decis Making* **2016**, *36*, 8-19, doi:https://doi.org/10.1177/0272989x15583266.

34. UK Government. National statistics: English indices of deprivation 2019. Available online: https://www.gov.uk/government/statistics/english-indices-of-deprivation-2019 (accessed on

35. *Technical Model Specifications Report: Public Health Impact and Cost-effectiveness Analysis of Bexsero (4CMenB) used for the Active Immunisation of Infants Against Invasive Meningococcal Serogroup B Disease in England*; GlaxoSmithKline Pharmaceuticals SA: 2021.

36. Beck, E.; Klint, J.; Garcia, S.; Abbing, V.; Abitbol, V.; Akerborg, O.; Argante, L.; Bekkat-Berkani, R.; Hogea, C.; Neine, M.; et al. Modelling the impact of 4CMenB and MenACWY meningococcal combined vaccination strategies including potential 4CMenB cross-protection: An application to England. *Vaccine* **2020**, *38*, 7558-7568, doi:https://doi.org/10.1016/j.vaccine.2020.08.007.

37. Marshall, H.S.; McMillan, M.; Koehler, A.P.; Lawrence, A.; Sullivan, T.R.; MacLennan, J.M.; Maiden, M.C.J.; Ladhani, S.N.; Ramsay, M.E.; Trotter, C.; et al. Meningococcal B Vaccine and Meningococcal Carriage in Adolescents in Australia. *New England Journal of Medicine* **2020**, *382*, 318-327, doi:https://doi.org/10.1056/NEJMoa1900236.

38. Collins, B. Results from a Well-Being Survey in the North West of England: Inequalities in EQ-5D 2013;Derived Quality-Adjusted Life Expectancy Are Mainly Driven by Pain and Mental Health. *Value in Health* **2017**, *20*, 174-177, doi:https://doi.org/10.1016/j.jval.2016.08.004.

39. Cleary, P.R.; Calvert, N.; Gee, S.; Graham, C.; Gray, S.; Kaczmarski, E.; Morphet, J.; Murphy, L.; Verlander, N.; Wood, T.; Borrow, R. Variations in Neisseria meningitidis carriage by socioeconomic status: a cross-sectional study. *Journal of Public Health* **2015**, *38*, 61-70, doi:https://doi.org/10.1093/pubmed/fdv015.

40. Read, R.C.; Baxter, D.; Chadwick, D.R.; Faust, S.N.; Finn, A.; Gordon, S.B.; Heath, P.T.; Lewis, D.J.; Pollard, A.J.; Turner, D.P.; et al. Effect of a quadrivalent meningococcal ACWY glycoconjugate or a serogroup B meningococcal vaccine on meningococcal carriage: an observer-blind, phase 3 randomised clinical trial. *Lancet (London, England)* **2014**, *384*, 2123-2131, doi:https://doi.org/10.1016/s0140-6736(14)60842-4.

41. Cookson, R.; Griffin, S.; Norheim, O.F.; Culyer, A.J. Distributional cost-effectiveness analysis: Quantifying health equity impacts and trade-offs. **2020**, doi:https://doi.org/10.1093/med/9780198838197.001.0001.

42. Yang, F.; Angus, C.; Duarte, A.; Gillespie, D.; Walker, S.; Griffin, S. Impact of Socioeconomic Differences on Distributional Cost-effectiveness Analysis. *Med Decis Making* **2020**, *40*, 606-618, doi:https://doi.org/10.1177/0272989x20935883.

43. Robson, M.; Asaria, M.; Cookson, R.; Tsuchiya, A.; Ali, S. Eliciting the level of health inequality aversion in England. *Health economics* **2017**, *26*, 1328-1334, doi:https://doi.org/10.1002/hec.3430.

44. National Institute for Health and Care Excellence (NICE). CHTE methods review: Modifiers. Task and finish group report. **2020**.

45. National Institute for Health and Care Excellence (NICE). Changes to NICE drug appraisals: what you need to know. Available online: https://www.nice.org.uk/news/feature/changes-to-nice-drug-appraisals-what-you-need-to-know (accessed on

46. National Institute for Health and Care Excellence (NICE). Consultation Paper. Value Based Assessment of Health Technologies. Available online: https://www.nice.org.uk/Media/Default/About/what-we-do/NICE-guidance/NICE-technology-appraisals/VBA-TA-Methods-Guide-for-Consultation.pdf (accessed on

47. National Institute for Health and Care Excellence (NICE). Guide to the methods of technology appraisal Available online: https://www.nice.org.uk/process/pmg9/chapter/foreword (accessed on 15-07-2021).

48. Office for National Statistics (ONS). Populations by Index of Multiple Deprivation, England, 2001 to 2019. Available online: https://www.ons.gov.uk/peoplepopulationandcommunity/populationandmigration/populationestimates/adhocs/12386populationbyindexofmultipledeprivationimdengland2001to2019 (accessed on 15/04/2022).

49. Bauwens, J.; de Lusignan, S.; Sherlock, J.; Ferreira, F.; Künzli, N.; Bonhoeffer, J. Adherence to the paediatric immunisation schedule in England. *Vaccine: X* **2021**, *9*, 100125, doi:https://doi.org/10.1016/j.jvacx.2021.100125.

50. Office for National Statistics (ONS). Health state life expectancies by Index of Multiple Deprivation (IMD 2015 and IMD 2019): England, all ages. Available online: https://www.ons.gov.uk/peoplepopulationandcommunity/healthandsocialcare/healthinequalities/datasets/healthstatelifeexpectanciesbyindexofmultipledeprivationimdenglandallages (accessed on 09-03-2023).

51. Ministry of Housing, Communities & Local Government,. Domains of deprivation. Available online: https://assets.publishing.service.gov.uk/government/uploads/system/uploads/attachment_data/file/467765/File_2_ID_2015_Domains_of_deprivation.xlsx (accessed on 15/04/2022).

52. HM Revenue & Customs. Percentile points from 1 to 99 for total income before and after tax. Available online: https://assets.publishing.service.gov.uk/government/uploads/system/uploads/attachment_data/file/1059906/Table_3.1a_1920.ods (accessed on 15/04/2022).

53. Joint Committee on Vaccination and Immunisation (JCVI). Minute of the meeting on Tuesday 11 and Wednesday 12 February 2014. Available online: https://app.box.com/s/iddfb4ppwkmtjusir2tc/file/229171703722 (accessed on June 11, 2018).

54. Lasseter, G.; Al-Janabi, H.; Trotter, C.L.; Carroll, F.E.; Christensen, H. The views of the general public on prioritising vaccination programmes against childhood diseases:

A qualitative study. *PloS one* **2018**, *13*, e0197374, doi:https://doi.org/10.1371/journal.pone.0197374.

55. Mielck, A.; Vogelmann, M.; Leidl, R. Health-related quality of life and socioeconomic status: inequalities among adults with a chronic disease. *Health and Quality of Life Outcomes* **2014**, *12*, 58, doi:https://doi.org/10.1186/1477-7525-12-58.

56. Pérez-Ardanaz, B.; Peláez-Cantero, M.J.; Morales-Asencio, J.M.; Vellido-González, C.; Gómez-González, A.; León-Campos, Á.; Gutiérrez-Rodríguez, L. Socioeconomic Factors and Quality of Life Perceived by Parents and Children with Complex Chronic Conditions in Spain. *Children* **2021**, *8*, 931, doi:https://doi.org/10.3390/children8100931.
